# The Tumor‐to‐Endothelial Transfer of FTO Promotes Vascular Remodeling and Metastasis in Nasopharyngeal Carcinoma

**DOI:** 10.1002/advs.202509524

**Published:** 2025-11-28

**Authors:** Chun Wu, Xuefei Liu, Liwen Gu, Jingru Lian, Yuting Wang, Yixin Cheng, Lianhui Duan, Guanyin Huang, Siqi Chen, Boxi Zhao, Sailan Liu, Yufan Yang, Shuqian Zheng, Zijian Lu, Wanping Guo, Jianyang Hu, Wenjing Wang, Zhixiang Zuo, Haiqiang Mai, LinQuan Tang, Songfa Zhang, Feiqiu Wen, Xin Hong, Ling Guo

**Affiliations:** ^1^ Department of Nasopharyngeal Carcinoma Sun Yat‐sen University Cancer Center State Key Laboratory of Oncology in South China Guangzhou 510060 China; ^2^ Department of Biochemistry SUSTech Homeostatic Medicine Institute School of Medicine Southern University of Science and Technology Shenzhen Guangdong 518055 China; ^3^ Department of Hematology and Oncology Shenzhen Children's Hospital and School of Medicine Southern University of Science and Technology Shenzhen 518026 China; ^4^ Shenzhen Children's Hospital of China Medical University Shenzhen 518026 China; ^5^ Department of Radiation Oncology Sun Yat‐sen University Cancer Center State Key Laboratory of Oncology in South China Collaborative Innovation Center of Cancer Medicine Guangzhou 510060 China; ^6^ Department of pathology Sun Yat‐sen University Cancer Center State Key Laboratory of Oncology in South China Guangzhou 510060 China; ^7^ School of Medicine Sun Yat‐sen University Shenzhen Guangdong 518107 China; ^8^ State Key Laboratory of Oncology in South China and Guangdong Provincial Clinical Research Center for Cancer Sun Yat‐sen University Cancer Center Guangzhou 510060 China; ^9^ Zhejiang Provincial Key Laboratory of Precision Diagnosis and Therapy for Major Gynecological Diseases & Department of Gynecologic Oncology Women's Hospital Zhejiang University School of Medicine Hangzhou 310011 China; ^10^ Key University Laboratory of Metabolism and Health of Guangdong Southern University of Science and Technology Shenzhen Guangdong 518055 China; ^11^ Guangdong Provincial Key Laboratory of Cell Microenvironment and Disease Research Southern University of Science and Technology Shenzhen Guangdong 518055 China

**Keywords:** exosome, FTO, nasopharyngeal carcinoma (NPC), NOTCH1, SPARC, vascular remodeling

## Abstract

Cancer stem‐like cells (CSCs) in nasopharyngeal carcinoma (NPC) exhibit heightened stemness, invasiveness, and resistance to therapy, posing significant challenges for diagnosis and treatment. How CSCs interact with the endothelium to drive vascular remodeling and metastasis remains unclear. Using spatially resolved multi‐omic profiling of clinical NPC samples and experimental validation, a tumor‐endothelial crosstalk mechanism is identified, driven by the NOTCH1–FTO–SPARC axis. Omics analysis reveals a distinct NOTCH1⁺ CSC subpopulation with enhanced tumorigenic potential. FTO, a direct transcriptional target and key effector of NOTCH1, promotes vascular remodeling and metastasis. Notably, NOTCH1⁺ tumors secreted FTO via exosomes, which are readily taken up by endothelial cells. Within recipient endothelial cells, accumulated FTO disrupts endothelial tight junctions by suppressing YTHDF2‐mediated m6A modification, stabilizing SPARC mRNA, and increasing SPARC protein levels. Inhibition of FTO with DAC51 significantly suppresses metastasis in NPC mouse models. Clinically, metastatic NPC patients show elevated levels of NOTCH1⁺ CSC‐CTCs (EPCAM⁺ EBNA1⁺ NOTCH1⁺ CD45^−^) and plasma FTO, suggesting their potential as blood‐based biomarkers for monitoring disease progression and treatment response. Overall, this study uncovers a non‐cell‐autonomous role of FTO in driving metastasis, with potential diagnostic and therapeutic utilities for NPC.

## Graphical abstract

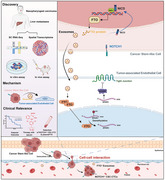

## 1 Introduction

Nasopharyngeal carcinoma (NPC) is a prevalent malignant tumor in southern China, originating from nasopharyngeal epithelial cells and often associated with the Epstein‐Barr virus (EBV) infection.^[^ ^1^ ^]^ Recent technological advances have improved the five‐year survival rate for locally advanced cases to over 80%, but this rate falls sharply down to 48% when distant metastasis occurs.^[^ ^2^, ^3^ ^]^ Metastatic lesions are usually multifocal, involving the skeleton, lymph nodes, lungs, and liver.^[^ ^4^ ^]^ Up to date, how NPC cells interact with the endothelium, remodel the vasculature, and metastasize to distant organs remains poorly understood.

Exosomes, a subtype of extracellular vehicles (EVs), play a vital role in mediating intercellular communication.^[^ ^5^ ^]^ Recent studies have revealed that certain exosome‐derived lncRNAs and miRNAs, such as Linc‐ROR, miR‐205‐5p and MiR‐144 are critical regulators of tumor cells and participate in vascular remodeling and tumor progression in NPC.^[^ ^6^, ^7^, ^8^ ^]^ Another study has reported that NPC exosomes, carrying two cell adhesion proteins, ICAM‐1 and CD44v5, affected vascular homeostatic state through enhancing endothelial cells migration, invasion.^[^ ^9^ ^]^ Although these studies have indicated the influence of exosomes on the tumor microenvironment, it remains unclear what key factors within the NPC exosomes play a metastasis‐promoting role.

FTO (Fat Mass and Obesity‐Associated Protein) is widely recognized as the first identified m6A demethylase, playing a central role in regulating N6‐methyladenosine (m6A) levels in mRNA.^[^ ^10^ ^]^ As a dynamic and reversible RNA epigenetic modification, m6A is deeply involved in critical biological processes such as gene expression regulation, RNA metabolism, and cell fate determination.^[^ ^11^ ^]^ In cancer, FTO modulates m6A modification levels on specific mRNAs, thereby influencing cancer cell aggressiveness, metastasis, and therapeutic resistance.^[^ ^12^, ^13^ ^]^ Recent studies have shown that FTO is frequently overexpressed in various cancers, including nasopharyngeal carcinoma (NPC), where its elevated expression is closely linked to tumor progression, poor prognosis, and reduced patient survival rates.^[^ ^14^ ^]^ How FTO regulates the tumor microenvironment (TME) has attracted significant attention.^[^ ^15^ ^]^ Furthermore, whether FTO possesses non‐cell autonomous function via exosome secretion to facilitate tumor‐microenvironment interactions remains largely unexplored.

In this study, we employed scRNA‐seq and spatial transcriptomics on matched primary tumors and liver metastases to investigate the molecular drivers of NPC metastasis. We identified a NOTCH1⁺ stem‐like subclone in primary tumors that likely seeded liver metastases via circulating CSC‐CTCs. The NOTCH1 activation in this subpopulation promoted the secretion of FTO protein via exosomes. The uptake of FTO‐containing exosomes by tumor‐associated endothelial cells triggered m⁶A demethylation and stabilization of SPARC mRNA in a YTHDF2‐dependent manner, resulting in the dysregulation of endothelial tight junctions. This unveils a non‐cell‐autonomous role of FTO in promoting vascular remodeling and metastasis. Therapeutically, the FTO inhibitor DAC51 showed strong anti‐tumor effects in NPC metastasis models. Clinically, elevated levels of NOTCH1⁺ CSC‐CTCs and plasma FTO were detected in metastatic patients, highlighting their potential as liquid biopsy biomarkers for NPC diagnosis and monitoring.

## 2 Results

### 2.1 Single‐Cell Transcriptomic Landscape of NPC

We performed scRNA‐seq on 37 samples from 26 NPC patients and 8 non‐cancerous nasopharyngeal patients, including 25 primary NPC, 2 liver metastases, 8 normal tissues, and 2 adjacent normal tissue (Table S1, Supporting Information). Meanwhile, we performed spatial transcriptomic RNA‐Seq to acquire in situ gene expression profiles of two patients with NPC. All NPC biopsies were histologically examined by H&E (Figure S1A, Supporting Information).

After quality control and batch correction, a total of 261777 single cells were included for the analysis. Using the classical features of different cell clusters, 8 cell types were identified, including B cells (56118; *CD19* and *MS4A1*), endothelial cells (5672; *PECAM1* and *VWF*), epithelial cells (21179; *KRT8* and *KRT17*), fibroblast cells (2950; *COL1A1* and *COL6A1*), mast cells (1714; *CPA3* and *MS4A2*), myeloid cells (24026; *CD14* and *FCGR3A*), plasma cells (36925; *TNFRSF17* and *IGHG1*), and T cells (113193, *CD3D* and *CD3E*) (Figure S1B,C, Supporting Information). The proportions of these 8 cell types in different tissue types, are shown in Figure S1D (Supporting Information). In normal tissues or primary tumors, the proportions of immune cells were comparatively elevated; however, in liver metastases, there was a predominance of epithelial cells, suggesting reduced immune cell infiltration. We also performed mIHC analysis on paired primary and liver metastatic NPC tissues. Quantitative analysis demonstrated a significant increase in EPCAM^+^ cells and a concomitant decrease in CD3^+^ T cells in liver metastases, aligning with scRNA‐seq clustering analysis showing diminished T subcluster (Figure S1E, Supporting Information).

### 2.2 NOTCH1^+^ CSCs Exhibit Enhanced Malignancy and Metastatic Potential

**Figure 1:**
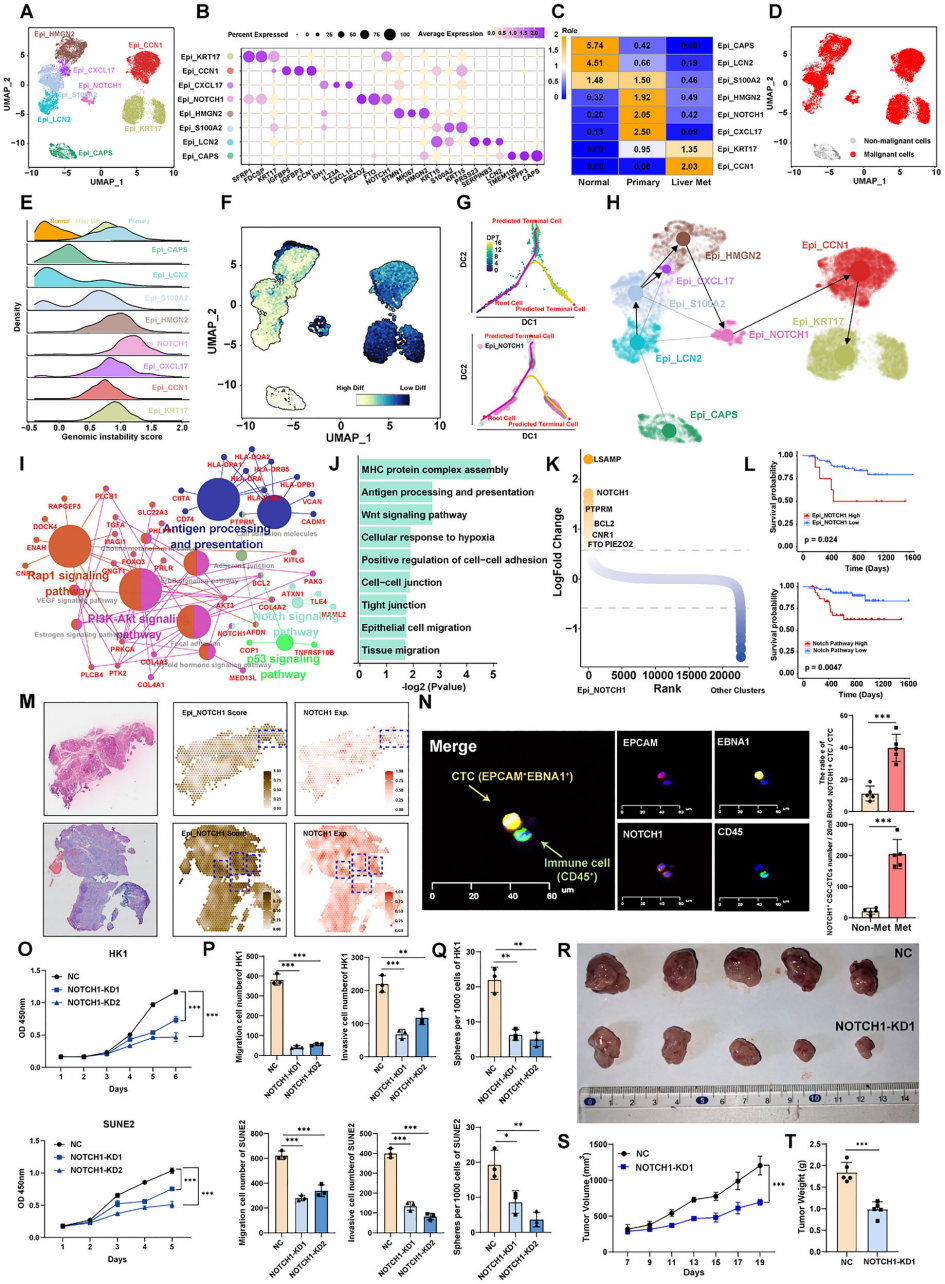
NOTCH1^+^ cancer stem‐like cells exhibit enhanced tumorigenicity and metastatic potential. A). UMAP plot showing the subtypes of epithelial cell, each dot indicates a single cell. Color‐coded for the cell type. B). Dot plot showing the selected markers for each subtype of epithelial cell. Dot size indicates the fraction of expressing cells, and the colors represent normalized gene expression levels. C). Tissue prevalence estimated by Ro/e score in epithelial cells. D). UMAP plot showing malignant and non‐malignant cells, as defined by inferCNV. E). Ridgeline plot showing the genomic instability score of eight malignant cell subtypes from three different tissues. F). UMAP plot showing the CytoTRACE scores of all epithelial cell subpopulations. CytoTRACE scores ranged from 0 to 1 with high scores indicating low differentiation status and low score indicating high differentiation. G). Diffusion pseudotime (DPT) trajectory analysis showing cellular transitions from the root cell to predicted terminal cells. The top plot shows the pseudotime, while the bottom plot shows the cells from Epi_NOTCH1 subpopulation. H). UMAP showing the RNA velocity and PAGA of 8 epithelial cell subtypes. I). Network diagram showing enriched KEGG pathways and their associated genes in Epi_NOTCH1 subpopulation. Larger nodes represent enriched pathways. J). Bar graph showing the pathway enriched in the Epi_NOTCH1 subpopulation. The x‐axis indicates the ‐log2 P value. K). Differential expressed genes between NOTCH1^+^ cancer stem‐like cells and the other cell subtypes. *Y*‐axis indicates the log2foldchange, genes ordered by log2foldchange along the *x*‐axis. L). Kaplan–Meier estimation of progression‐free survival (PFS) time in NPC patients by Epi_NOTCH1 subpopulation signature score (top) and Notch pathway activity score (bottom). M). H&E‐stained tissue (left panel), Epi_NOTCH1 subpopulation signature score (middle panel) and the expression levels of NOTCH1 (right panel) based on spatial transcriptomic analysis of primary NPC sample from patients. N). Multi‐immunofluorescence (mIF) staining of circulating tumor cells (CTCs) showing DAPI (nuclei), EPCAM (epithelial marker), NOTCH1, and CD45 (leukocyte marker) (left panel). EPCAM^+^EBNA1^+^ cells identified as CTCs. CD45^+^ cells are identified as immune cells. Barplot showing the percentage and number of NOTCH1^+^ CSC‐CTCs between non‐metastasis and metastasis NPC patients (right panel). O). CCK‐8 assay showing the effect of NOTCH1 knockdown on the proliferation of HK1 and SUNE2 cells, demonstrating reduced cell growth upon NOTCH1 silencing. P). Transwell migration and invasion assays assessing the impact of NOTCH1 knockdown on the migration and invasion abilities of HK1 and SUNE2 cells, with significant inhibition observed in both cell lines. Q). The effect of NOTCH1 expression change (NOTCH1‐KD) on NPC cell sphere‐propagating capacity. R–T). 5 × 10^6^ WT HK1 or NOTCH1‐KD HK1 cells were hypodermic injected into 8 weeks old nude mice, *n* = 5 per group. Photograph R), Tumors growth curves S), Quantitative analysis T). Data in (O–Q) are mean ± SD from 3 independent experiments, *p < 0.05, **p < 0.01, and ***p < 0.001 were determined by two‐way ANOVA with Bonferroni's post test.

The epithelial cells expressing *EPCAM*, *KRT8*, and *KRT18* (Figure S2A, Supporting Information) were further subclassified, and the 8 cell subtypes were found in all patients (**Figure** **1**A). Each cell subtype had its specific transcriptional signatures (Figure 1B). Ciliated epithelial cells highly expressing *CAPS*, *TPP3*, and *TMEM190* were identified in normal nasopharyngeal tissues (Figure 1C; Figure S2A,B, Supporting Information). LCN2^+^ epithelial cells and S100A2^+^ epithelial cells were also enriched in normal nasopharyngeal tissues. HMGN2^+^ epithelial cells, NOTCH1^+^ epithelial cells, and CXCL17^+^ epithelial cells were enriched in primary NPC samples. Furthermore, KRT17^+^ epithelial cells and CCN1^+^ epithelial cells were enriched in liver metastasis (Figure 1C; Figure S2B, Supporting Information). Copy‐number variation (CNV) analysis from single‐cell transcriptomes was employed to define malignant and non‐malignant epithelial cells (Figure 1D; Figure S2C, Supporting Information). In patient P3, concurrent whole‐exome sequencing was conducted to assess the genomic copy number variations. Our results revealed the genomic alterations in chromosomes 2, 4, 11, 14, 16, and 22, which were well‐aligned with the inferences drawn from single‐cell analyses (Figure S2C,D, Supporting Information). Meanwhile, we also determined that NOTCH1^+^ malignant cells had significantly higher genomic instability than other malignant cells (Figure 1E). We then applied CytoTRACE^[^ ^16^ ^]^ to predict the differentiation states of all epithelial cells. Strikingly, the primary tumor‐specific NOTCH1^+^ malignant subclone exhibited a much less differentiated state compared to other subclones within the primary tumor. And the differentiation state of NOTCH+ subclone was highly similar to the liver met‐specific subclones (KRT17^+^ and CCN1^+^ subpopulations) (Figure 1F; Figure S2E, Supporting Information). The landscape single cell entropy analysis suggested that NOTCH1^+^ malignant cells served as the progenitor cells for these stem‐like subclones (Figure 1G). Consistently, the RNA velocity analysis indicated that the flow directions were from the NOTCH1^+^ cancer stem‐like subclone to KRT17^+^ and CCN1^+^ malignant subpopulations (Figure 1H). Pathway enrichment analysis identified that NOTCH1^+^ cancer stem‐like cells exhibited upregulations of NOTCH pathway, Wnt pathway, cell‐cell junction pathway (Figure 1I,J), with *NOTCH1* as one of the top elevated genes (Figure 1K; Figure S3A, Supporting Information). Patients with a higher NOTCH1^+^ stemness signature score or a higher NOTCH pathway score had significantly shorter progression‐free survival time in a GEO RNA‐seq dataset (Figure 1L). The spatial transcriptomic analysis also showed a strong co‐localization between *NOTCH1* expression and NOTCH1^+^ stemness signature score (Figure 1M). These data collectively suggested that the cancer stem‐like NOTCH1^+^ subclone found in the primary tumor was likely the pre‐metastatic population giving rise to liver metastasis. This result encouraged us to assess if NOTCH1^+^ CSCs cells can be found in the blood circulation as circulating tumor cells (CTCs) while they were metastasizing to the liver. CTCs were isolated from metastatic and non‐metastatic patient blood samples, and NOTCH1 expression was assessed by multiplexed immunofluorescence (mIF). Tumor‐specific markers EPCAM and EBNA1 (the Epstein‐Barr virus‐encoded nuclear antigen) were used to identify CTCs, while CD45 was used to mark immune cells. Remarkably, EPCAM^+^ EBNA1^+^ CD45^−^ CTCs were detected from NPC blood samples, with significantly higher counts observed in metastatic patients as compared to non‐metastatic group (Figure S3B, Supporting Information). Moreover, NOTCH1^+^ cancer stem‐like CTCs (CSC‐CTC), marked by EPCAM^+^ EBNA1^+^ NOTCH1^+^ CD45^−^, were also present in circulation. Furthermore, the total number and proportion of these NOTCH1^+^ CSC‐CTCs were markedly elevated in metastatic NPC patients compared to the non‐metastatic group (Figure 1N; Figure S3C, Supporting Information). Therefore, we have provided substantial evidence confirming the presence of NOTCH1^+^ CSC‐CTCs in NPC patients and identified their strong association with metastatic progression.

To test the functional significance of NOTCH1 in NPC progression, genetic manipulation experiments were carried out in two NPC cell lines. NOTCH1 depletion effectively reduced the expression of NOTCH1 and its downstream target genes HES1 and RBPJ, which served as notch pathway reporter genes^[^ ^17^ ^]^ (Figure S3D,E, Supporting Information). Stemness‐related genes such as CD44 and CD24 were also significantly downregulated (Figure S3D,E, Supporting Information). Functionally, NOTCH1 depleted cells exhibited impaired cell proliferation (Figure 1O), migration, invasion (Figure 1P; Figure S3F, Supporting Information) and tumor sphere formation (Figure 1Q; Figure S3F, Supporting Information). Remarkably, the tumorigenic potential of HK1 cells were also significantly dampened upon NOTCH1 depletion, yielding substantially smaller tumors compared to the control group (Figure 1R–T). Conversely, the NOTCH1 overexpression (NOTCH1‐OE) NPC cell lines activated NOTCH1 signaling pathway genes and stemness signatures, exhibiting enhanced cellular proliferation, migration, invasion, and sphere‐forming capabilities (Figure S3H–J, Supporting Information). Together, these results indicated that NOTCH1 activity marked a subclone of cancer stem‐like NPC cells linked to substantially increased stemness, invasiveness, tumorigenicity, and metastatic potential.

### 2.3 NOTCH1 Regulates FTO Expression and Activity

**Figure 2:**
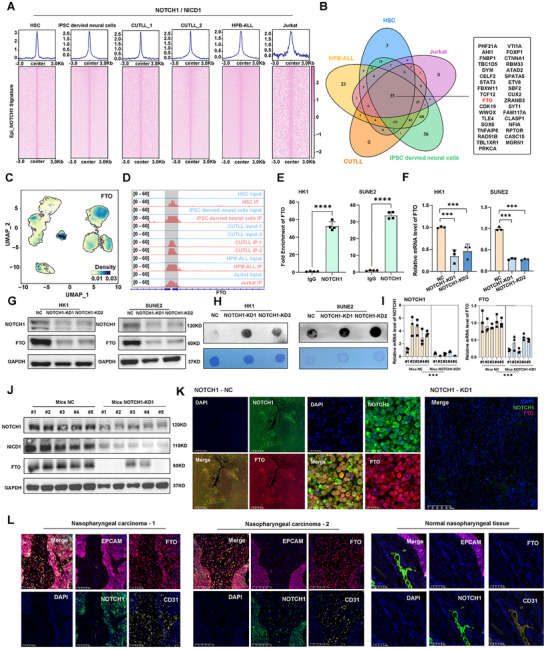
NOTCH1 regulates the expression of m6A mRNA demethylase FTO. A). Aggregation plots (top) and heatmaps (bottom) showing the NOTCH1 binding levels in HSC, iPSC‐derived neural cells, CUTLL1, CUTLL2, HPB‐ALL, and Jurkat cell lines. NOTCH1 Chip‐seq peaks within signatures from Epi_NOTCH1 were sorted in descending order based on the mean RPKM value per region. B). Venn diagram showing the overlap of NOTCH1 target genes based on NOTCH1 chip‐seq data across multiple cell lines, including HSC, HPB‐ALL, Jurkat, CUTLL, and iPSC‐derived neural cells. C). UMAP plot showing the density distribution of FTO expression across epithelial subpopulations. D). Binding of NOTCH1 to the FTO gene in HSC, HPB‐ALL, Jurkat, CUTLL, and iPSC‐derived neural cells. All peaks were called by MACS2 (*p* < 0.05). E). ChIP‐PCR assay was performed to detect the extent of NOTCH1 binding to the FTO gene in HK1 and SUNE2 cells. F). RT‐qPCR analysis showing the mRNA expression levels of *FTO* in HK1 and SUNE2 cells following NOTCH1 knockdown. G). Western blot analysis displaying the NOTCH1 and FTO expression in HK1 and SUNE2 following NOTCH1 knockdown. H). Dot blot analysis showing increased m6A modification levels in both HK1 and SUNE2 cells after NOTCH1 knockdown. I). RT‐qPCR analysis showing relative mRNA levels of *NOTCH1* and *FTO* in xenograft tumors. J). Western blot analysis of NOTCH1, NICD1 (the intracellular domain of NOTCH1), and FTO in xenograft tumors with NOTCH1 knockdown. K). Multiplex immunohistochemical (mIHC) images showing the expression of NOTCH1 and FTO in xenograft tumors. In the NOTCH1 knockdown group, FTO expression is substantially diminished, as visualized by DAPI staining and merge panels. L). mIHC showed the expression of FTO, EPCAM, NOTCH1, and CD31 in NPC tissues versus normal nasopharyngeal tissues. Data in (E,F and I) are mean ± SD from independent experiments, ***p < 0.001 and ****p < 0.0001 were determined by two‐way ANOVA with Bonferroni's post test.

The activation of the NOTCH signaling pathway is contingent upon the transcriptional modulation mediated by the Notch intracellular domain (NICD) and its co‐factor RBPJ.^[^ ^18^ ^]^ To further elucidate the direct targets of NOTCH1/NICD1, we conducted chromatin immunoprecipitation (ChIP)‐sequencing analysis on six samples from five cell lines using publicly available datasets. NICD1 chromatin binding level from these cell lines was substantially increased in NOTCH1^+^ cancer stem‐like subclones (**Figure** **2**A). By overlapping analysis of all ChIP‐sequencing data, 35 candidate targets were selected (Figure 2B). To our surprise, a classic m6A demethylase (“Eraser”), Fat Mass and Obesity‐associated protein (FTO), was one of the top enriched genes (Figure 2B). Furthermore, FTO was also selectively elevated in NOTCH1^+^ cancer stem‐like cell subpopulation (Figure 2C). The binding of NICD on FTO promoter was confirmed by ChIP‐sequencing and ChIP‐qPCR in HK1 and SUNE2 cell lines (Figure 2D,E). Consistently, knockdown of NOTCH1 in the two NPC cell lines resulted in significant downregulation of FTO expression at both mRNA and protein levels (Figure 2F,G). As an eraser enzyme, FTO negatively impacts m6A modifications.^[^ ^19^ ^]^ As expected, NOTCH1‐KD cells exhibited substantially enhanced m6A modifications as shown by the dot plot assay (Figure 2H). Interestingly, the knockdown of NOTCH1 in HUVEC did not affect FTO expression at protein levels, showing cell type‐specific regulation of FTO by NOTCH1 activity (Figure S4A, Supporting Information). A significant reduction in both FTO RNA and protein levels in NOTCH1‐knockdown mice tumors was also evident compared to control tumors (Figure 2I,J). Additionally, hematoxylin‐eosin staining (H&E) and multiplexed‐immunohistochemistry (mIHC) were performed on mice tumor tissue sections, confirming that NOTCH1‐KD mice tumors exhibited a strong reduction in NOTCH1 and FTO proteins as compared to the control group (Figure 2K; Figure S4B,C, Supporting Information). Finally, we performed mIHC analysis on NPC patients and normal nasopharyngeal tissues. Interestingly, FTO protein was detected abundantly in both EPCAM^+^ NOTCH1^+^ malignant cells as well as in tumor‐associated CD31^+^ endothelial cells, but barely detectable in normal nasopharyngeal tissue (Figure 2L). Intriguingly, the expression of stemness‐related genes such as CD44 and CD24 and sphere‐forming capacity were not significantly changed between control cells and the FTO overexpression (FTO‐OE) NPC cells, suggesting FTO was not involved in NPC CSCs maintenance (Figure S5A,B, Supporting Information). Collectively, these results suggested that NOTCH1/NICD transcriptionally promoted FTO expression and modulated the overall abundance of m6A modifications in NPC cells.

### 2.4 The Tumor‐to‐Endothelial Transfer of FTO Protein Impacts Endothelial Integrity

**Figure 3:**
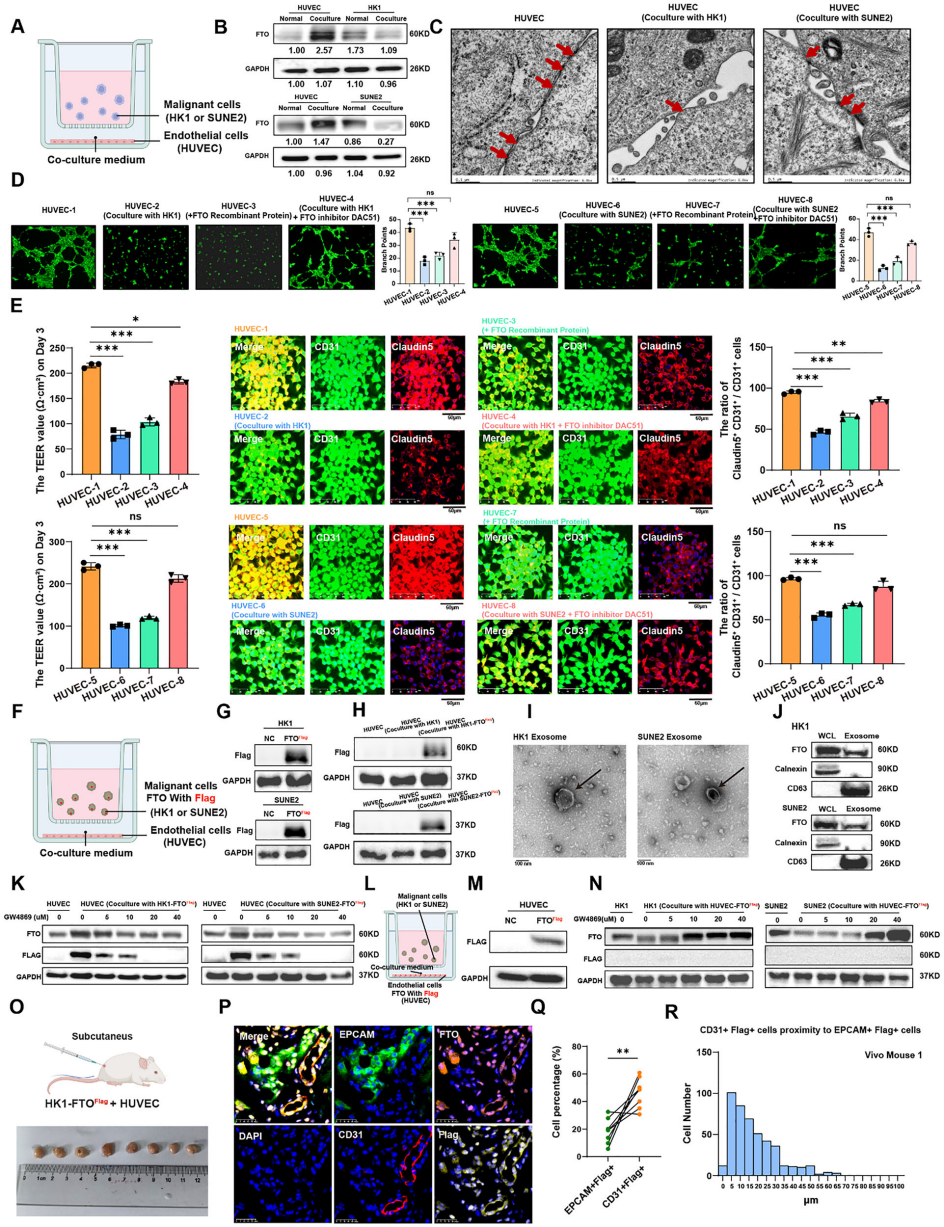
Tumor‐derived FTO proteins are secreted via exosomes and taken up by endothelial cells to modulate vascular permeability. A). Schematic of the indirect co‐culture system used to study interactions between malignant cells (HK1/SUNE2) and endothelial cells (HUVECs). B). Western blot analysis of FTO expression in HUVECs, HK1, and SUNE2 cells, under cultured alone (normal) and co‐culture conditions. C). Electron microscopy showing the ultrastructure of HUVECs cell junctions under cultured alone or co‐culture conditions. D). The tube formation assay showing tube‐forming ability of HUVECs under cultured alone, co‐cultured with malignant cells, added FTO recombinant protein, and co‐cultured with malignant cells treated with FTO inhibitor DAC51. The statistical results are shown on the right. E). The values of trans‐endothelial electrical resistance (TEER) showing permeability of HUVECs under cultured alone, co‐cultured with malignant cells, added FTO recombinant protein, and co‐cultured with malignant cells treated with FTO inhibitor DAC51 in day 3 (left panel). mIHC showing the tight junction markers Claudin‐5 expression in endothelial cells (middle panel) and bar plot showing comparison of the proportion of Claudin‐5^+^CD31^+^ /CD31^+^ cells in different groups (right panel). F). Schematic of indirect co‐culture system using Flag‐FTO malignant cells (HK1/SUNE2) to trace FTO transfer between malignant and endothelial cells. G). Western blot showing Flag‐tagged FTO HK1 and SUNE2 cells were successfully constructed and verified. H). Western blot showing after co‐culture with Flag‐FTO HK1 or SUNE2 cells, HUVEC cells showed high expression of Flag protein. I). Negative‐staining electron microscopy showing exosomes collected from conditioned medium. J). Western blot showing the exosome positive (CD63) and negative markers (Calnexin) confirming exosome isolation from the co‐culture system and the protein expression level of FTO in exosomes from HK1 and SUNE2 cells. K). Western blot showing FTO and Flag protein expression levels in HUVECs after the addition of the exosome inhibitor GW4869 to the co‐culture system of HUVEC cells with Flag‐FTO HK1 cells or Flag‐FTO SUNE2 cells. L). Schematic of indirect co‐culture system using Flag‐FTO endothelial cells (HUVEC) to trace FTO transfer between malignant and endothelial cells. M). Western blot showing Flag‐FTO HUVECs were successfully constructed and verified. N). Western blot showing FTO and Flag protein expression levels in HK1 and SUNE2 cells after the addition of the exosome inhibitor GW4869 to the co‐culture system of Flag‐FTO HUVEC cells with HK1 cells or SUNE2 cells. O). Schematic of subcutaneous tumor models in nude mice. The diagram illustrates the injection of HK1 with Flag‐FTO (1×10^6^) and HUVEC (1×10^6^) cells into the subcutaneous space of the mouse. The photograph below shows the resulting tumor masses, aligned next to a ruler to indicate tumor size. P). mIHC showing staining of tumor tissue sections from subcutaneous tumor models with DAPI (blue) for nuclei, CD31 (red) for endothelial cells, EPCAM (green) for epithelial cells, FTO (orange) for target protein, and Flag (yellow) for target protein. The merged image illustrates the localization of these markers. Q). Paired dot plot compares the percentage of EPCAM^+^ Flag^+^ cells and CD31^+^ Flag^+^ cells in tumor tissue. Each line connects paired samples, highlighting the increase in cell percentage from EPCAM^+^ Flag^+^ to CD31^+^ Flag^+^ cells. R). Bar chart showing the proximity of CD31^+^ Flag+ cells to EPCAM^+^ Flag+ cells in tumor tissue from Vivo Mouse1. Data in (D and E) are mean ± SD from 3 independent experiments, *p < 0.05 and ***p < 0.001 were determined by two‐way ANOVA with Bonferroni's post test.

In NPC patients, we observed not only elevated levels of FTO protein in malignant cells but also a prominent expression of FTO in CD31^+^ endothelial cells that were in close proximity to the tumor area (Figure 2L). We speculated that NOTCH1+ tumor cells may crosstalk with the neighboring endothelial cells and induce concurrent expression of FTO. To explore the potential molecular crosstalk mechanisms, an indirect co‐culture system incorporating nasopharyngeal carcinoma (NPC) tumor cell lines (HK1 and SUNE2) and a human umbilical vein endothelial cell line (HUVEC) was established (**Figure** **3**A). Surprisingly, endothelial cells within the serum‐free co‐culture system showed a significant upregulation of FTO protein expression at the expense of reduced FTO protein abundance within the co‐cultured tumor cells. This phenomenon was consistently observed when either HK1 or SUNE2 tumor cells were co‐cultured with HUVEC (Figure 3B). This suggested a potential cell‐to‐cell transfer of FTO protein might take place between tumor and endothelial cells in our experimental system. To assess the functional impact of tumor cells on co‐cultured endothelial cell structure and function, transmission electron microscopy (TEM) was applied for the analysis. Our results suggested a strong disruption of inter‐endothelial cell‐cell junctions (Figure 3C). To further test if the exchange of FTO proteins between tumor cells and endothelial cells in the co‐culturing system was functionally important for endothelial integrity and function, the tube‐forming ability of endothelial cells, and trans‐endothelial electrical resistance (TEER) were assessed in four experimental groups, including HUVEC alone, HUVEC co‐cultured with malignant cells, HUVEC co‐cultured with FTO recombinant protein, and HUVEC co‐cultured with malignant cells treated with DAC51, a small‐molecule inhibitor of FTO.^[^ ^20^ ^]^ Remarkably, our data showed that tumor cells significantly disrupted the structural and functional integrity of the co‐cultured endothelial sheets in a manner dependent on the presence of FTO proteins (Figure 3D,E; Figure S6A, Supporting Information). This is because 1) the supplementation of FTO recombinant protein in HUVEC cells phenocopied endothelial disruption phenotype as shown in tumor‐HUVEC cocultures, and 2) blocking FTO protein function by using its inhibitor DAC51 partially rescued the disrupted endothelial tubes and the restoration of TEER (Figure 3D,E; Figure S6A, Supporting Information). These data collectively suggested FTO was a critical mediator of tumor cell‐induced endothelial disruption. To assess whether the accumulation of FTO within HUVEC cells was derived from co‐cultured tumor cells, the upstream regulator of FTO, NOTCH1, was depleted in HK1 or SUNE2 cells (NOTCH1‐KD), which were co‐cultured with HUVEC. Interestingly, there was a substantial reduction of FTO in HUVEC cells co‐cultured with NOTCH1‐KD tumor cells as compared to the HUVECs co‐cultured with control tumor cells (Figure S6B, Supporting Information). Furthermore, a significant relief in the degree of endothelial tube disruption, the defects in TEER, and the partial rescue of impaired endothelial Claudin5 expression, were consistently observed in HUVEC co‐cultured with NOTCH1‐KD tumor cells compared to the control group (Figure S6C,D, Supporting Information). These data implicated that tumor‐specific NOTCH1 activity induced FTO expression and protein secretion, which was taken up by the co‐cultured HUVEC cells, and consequently, the accumulated FTO protein HUVEC induced endothelial junctional dysfunction.

To further characterize the detailed biological process of tumor‐endothelial transfer of FTO protein, two tumor cell lines expressing Flag‐tagged FTO protein were established for co‐culturing with HUVEC cells (Figure 3F,G). Consistent with our hypothesis, the presence of the Flag‐FTO protein in HUVEC cells was confirmed by western blotting analysis, indicating a direct transfer of Flag‐FTO from tumor to endothelial cells (Figure 3H). We further speculated that tumor‐derived FTO protein might be transferred to the endothelial cells through the secretion of exosomes. Indeed, FTO protein was reliably detected in exosomes isolated from the two tumor cell lines (Figure 3I,J; Figure S7A, Supporting Information). To test if exosomes mediate cell‐to‐cell FTO transfer, the co‐culturing system was treated with GW4869, which blocks exosome biogenesis and secretion. Interestingly, the accumulation of Flag‐FTO in HUVEC cells was significantly impaired upon GW4869 treatment, suggesting Flag‐FTO was transferred from tumor cell‐derived exosomes, which were subsequently taken up by HUVEC cells (Figure 3K). Importantly, the cell‐autonomous expression of native FTO in HUVEC cells was not strongly affected by GW4869 treatment (Figure S7B, Supporting Information). To monitor if such cell‐to‐cell transfer of FTO was also operational from HUVEC‐to‐tumor cells, Flag‐FTO transgenic HUVEC cells were established and co‐cultured with wild‐type HK1 or SNUE2 tumor cells. Strikingly, upon GW4869 treatment, HUVEC‐derived transgenic Flag‐FTO protein was not detected in tumor cells, despite the native tumor cell‐specific FTO protein accumulated, presumably due to the blocked secretion of FTO‐containing exosomes within these tumor cells by GW4869 (Figure 3L,N). Collectively, these data indicated FTO protein was preferentially transferred from the tumor to the endothelial cells, but not vice versa. This process was likely mediated by tumor‐derived FTO‐containing exosome secretion and the uptake of exosomal FTO by endothelial cells. Such non‐cell autonomous tumor‐to‐endothelial transfer of FTO was critical for regulating endothelial integrity and function, as evidenced by the impaired tight junction gene expression, tube forming capability and trans‐endothelial electrical resistance in recipient endothelial cells with abundant accumulation of FTO proteins.

Given the extensive heterogeneity inherent to malignant cells, we used flow cytometry to isolate NOTCH1^+^ and NOTCH1^−^ subpopulations from HK1 and SUNE2 cell lines, followed by knockdown of NOTCH1 in the NOTCH1^+^ malignant subpopulation (Figure S8A,B, Supporting Information). Compared to NOTCH1^−^ subpopulation and NOTCH1^+^ group with NOTCH1 knockdown, NOTCH1^+^ subpopulation exhibited elevated expression of NOTCH1 and stemness‐related genes such as CD44 and CD24 (Figure S8C, Supporting Information) and showed enhanced sphere‐forming capabilities (Figure S8D, Supporting Information). The NOTCH1^+^ subpopulation also showed stronger disruption of the endothelial tube formation assay, TEER, and the endothelial Claudin5 expression (Figure S8E–G, Supporting Information). Analysis of exosomes from the three subpopulations suggested that FTO protein was predominantly enriched in the NOTCH1^+^ subpopulation (Figure S8H, Supporting Information).

To test if the tumor‐to‐endothelial transfer of FTO occurs in vivo, transgenic HK1 expressing Flag‐FTO was co‐cultured with HUVEC cells and implanted into nude mice subcutaneously. The tumor and endothelial localization of Flag‐FTO was assessed by mIHC assay (Figure 3O; Figure S9A, Supporting Information). Strikingly, there was a significant co‐localization of FTO, Flag, and CD31 proteins in these tumor sections. Although Flag‐FTO was tumor‐derived, there was much more abundant Flag‐FTO detected in CD31^+^ endothelial cells than EPCAM^+^ tumor cells nearby, suggesting the transfer of Flag‐FTO from tumor to endothelial cells indeed occurred during in vivo tumor growth (Figure 3P,Q). Consistently, the spatial distribution analysis demonstrated abundant EPCAM^+^ Flag^+^ tumor cells localized to the vicinity of the CD31^+^ Flag^+^ endothelial cells (Figure 3R; Figure S9B, Supporting Information). Together, these results suggested that NPC tumor cells are capable of transferring FTO protein to endothelial cells via exosome secretion, and the uptake of tumor‐derived FTO protein by endothelial cell‐cell junction disruptions and vascular permeability.

### 2.5 FTO Promotes Metastatic Colonization and the FTO Inhibitor DAC51 Demonstrates In Vivo Anti‐Tumor Effects

**Figure 4:**
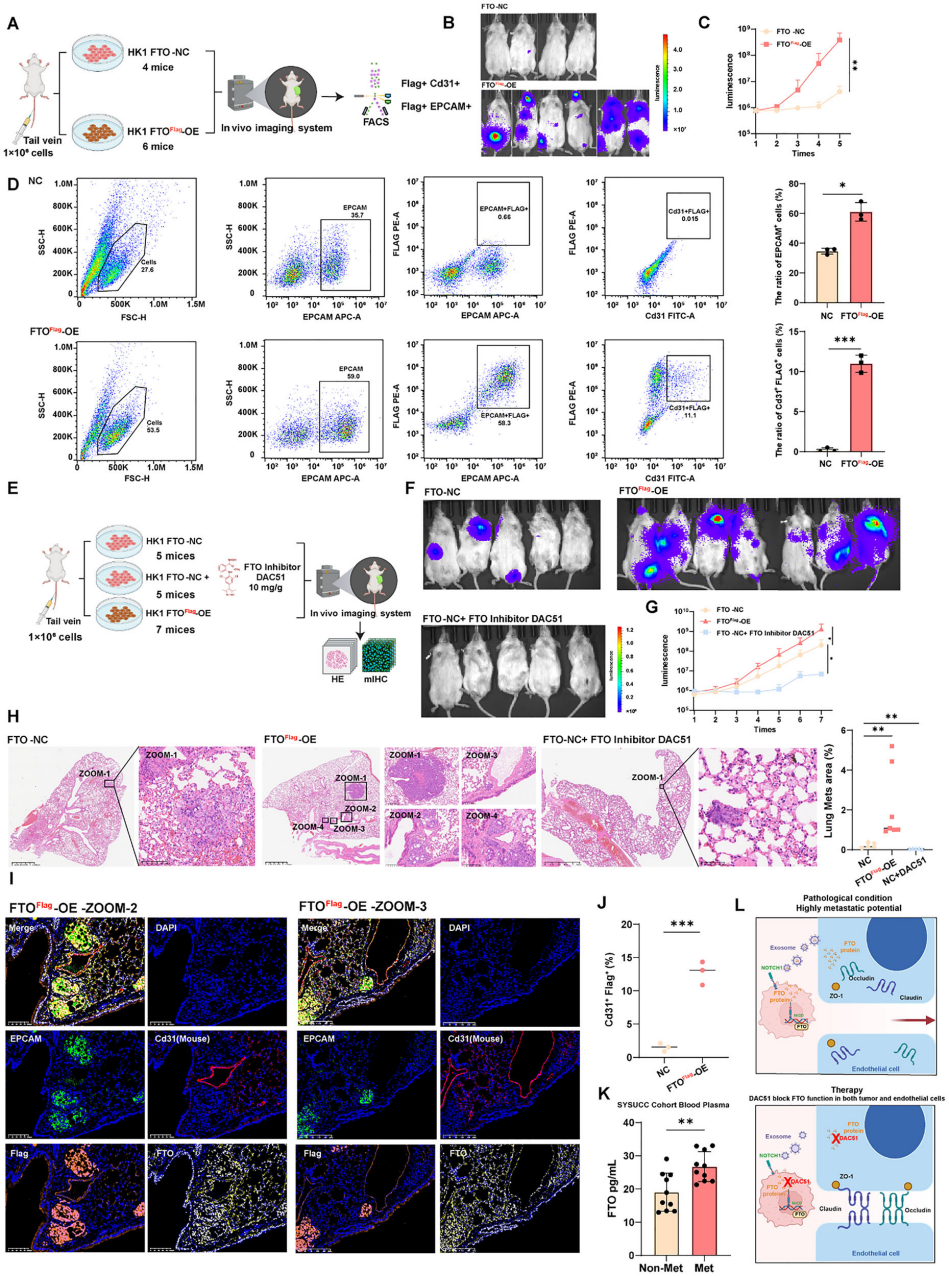
FTO regulates NPC metastatic colonization, and the FTO inhibitor DAC51 possesses anti‐tumor effects in vivo. A). Flow chart depicting in vivo experiment of tail vein injection of HK1 to immunocompromised NCG mice. Control group N = 4 mice, FTO overexpression group N = 6 mice. B). Tumor metastasis experiment established by injecting HK1 NPC cells expressing luciferase‐GFP to NCG mice through the tail vein. Picture shows the IVIS images captured at the experiment endpoint. C). Metastases growth curve indicated by luciferase signal of whole mouse detected by IVIS system for control and FTO overexpression group. D). The number of EPCAM^+^ cells and Cd31^+^ Flag^+^ doublet positive cells from lung tissue in control or FTO overexpression group mice by flow cytometry (left panel). The ≈35% EPCAM+ cells reflected the high burden of these single disseminated tumor cells/micro‐metastases that are present but not easily scorable by conventional H&E histology. Summary data of cell number (right panel) are presented as mean ± SD (*n* = 3), *p < 0.05, ***p < 0.001; E). Flow chart depicted in vivo experiment of tail vein injection of HK1 to immunocompromised NCG mice. Control group N = 5 mice, FTO inhibitor DAC51 treatment group N = 5 mice, FTO overexpression group N = 7 mice. F). Tumor metastasis experiment established by injecting HK1 NPC cells expressing luciferase‐GFP to NCG mice through the tail vein. Picture shows the IVIS images captured at the experiment endpoint. G). Metastases growth curve indicated by luciferase signal of whole mouse detected by IVIS system for control, DAC51 treatment and FTO overexpression group. H). H&E staining of lungs dissected from the metastasis mice model of HK1 NPC cells with or without FTO overexpression or DAC51 treatment. I). mIHC images showing the expression of EPCAM (green), FTO (yellow), Cd31 (red) and Flag (orange) in xenograft tumors in different groups. J). Bar plot showing the percentage of Cd31^+^ Flag^+^ cells in xenograft tumors from different experimental groups. K). Bar plot showing FTO protein levels in blood plasma by ELISA in the SYSUCC cohort. Patients with distant metastases N = 10, Patients without distant metastases N = 10. L). Schematic illustration showing FTO‐mediated endothelial disruption and therapeutic strategies involving FTO inhibitor DAC51.

To test if Flag‐FTO overexpression (Flag‐FTO OE) affects the metastatic potential in NPC, we established in vivo mice models of metastasis using tail vein injections (**Figure** **4**A). Flag‐FTO OE HK1 cells showed substantially enhanced metastatic outgrowth at 5 weeks post‐injection in immunocompromised NCG mice (Figure 4B,C; Figure S10A, Supporting Information). H&E staining also confirmed the formation of multiple metastases in the lung or liver in FTO‐OE group (Figure S10B,C, Supporting Information). Meanwhile, the H&E analysis also showed the presence of metastases in other organs, such as the heart and skin, in the FTO‐OE group (Figure S10D, Supporting Information). Interestingly, the ratio of EPCAM^+^ cells and Cd31^+^ Flag^+^ double positive cells was also increased in the group of Flag‐FTO OE cells compared with the control group using flow cytometric analysis (Figure 4D). Next, we assessed the anti‐tumor effect of FTO inhibitor DAC51 in this mouse model (Figure 4E; Figure S10E, Supporting Information). Strikingly, HK1 tumor cells with DAC51 treatment exhibited a dramatic reduction of metastatic burden in the lung and liver compared to the control group (Figure 4F,G; Figure S10F, Supporting Information), which was confirmed by H&E staining and quantifications (Figure 4H; Figure S10G, Supporting Information). H&E staining also confirmed the formation of multiple metastases in the lung or liver, in close proximity to the vasculature in Flag‐FTO OE mouse group (Figure 4H; Figure S10G, Supporting Information). mIHC assay was applied to confirm the existence of large areas of EPCAM^+^ Flag^+^ FTO^+^ malignant cells in the Flag‐FTO OE mouse group, which was found to be in close spatial proximity to Flag^+^ Cd31^+^ endothelial vasculatures (Figure 4I; Figure S10H, Supporting Information). The Cd31+ Flag+ double positive cells were found to be significantly elevated in Flag‐FTO OE tumors (Figure 4J). Conversely, DAC51 treatment in NC mouse group resulted in substantial downregulation of the fraction of the Sparc^+^ Cd31^+^ cells and the upregulation of the fraction of Cldn5^+^ Cd31^+^ cells among total Cd31^+^ cells as compared the untreated NC group (Figure S10I, Supporting Information). These data collectively suggested a causal role of FTO in regulating endothelial SPARC expression, junctional integrity, and metastatic progression in vivo.

Finally, in clinical samples from the SYSUCC plasma cohort, we quantified FTO protein levels by enzyme‐linked immunosorbent assay (ELISA). Notably, FTO protein expression was significantly increased in patients with distant metastases compared to the non‐metastatic group (Figure 4K). Thus, the tumor‐to‐endothelial transfer of FTO also occurred in vivo in our mouse metastasis assay. FTO overexpression in tumor cells is capable of directly promoting metastatic progression, and the FTO inhibitor DAC51 was shown to be highly effective in suppressing metastasis in vivo (Figure 4L). The elevated levels of secreted FTO detected in plasma samples of metastatic NPC patients compared to the non‐metastatic group suggested a strong clinical relevance to our findings.

### 2.6 FTO Promotes Endothelial Dysfunction by Regulating m6A Modification of SPARC

**Figure 5:**
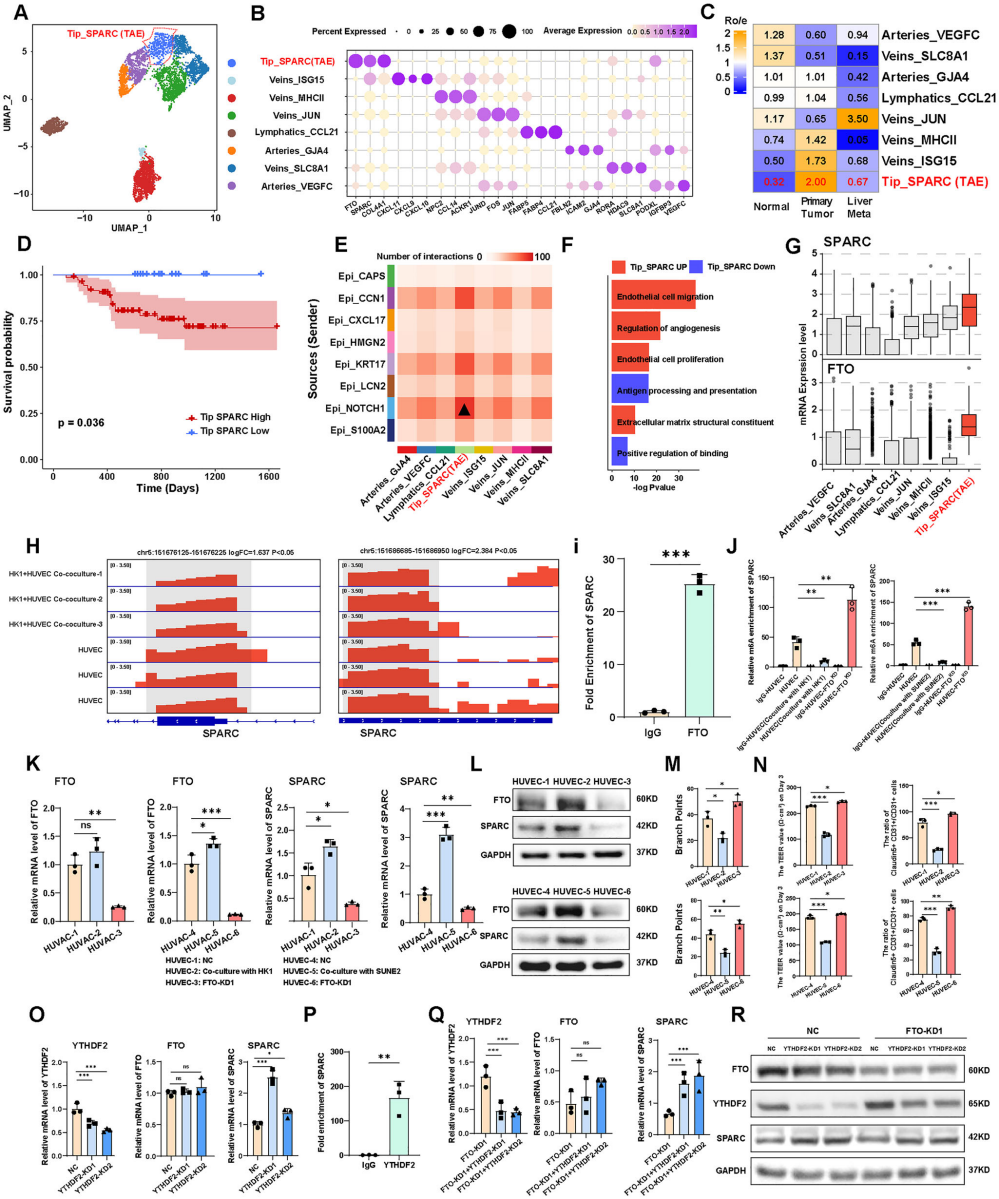
FTO disrupts endothelial integrity by promoting SPARC release in an m6A‐YTHDF2‐dependent manner. A). UMAP plot showing the subtypes of endothelial cell, each dot indicated a single cell. Color‐coded for the cell type. B). Dot plot showing the selected markers for each subtype of endothelial cell. Dot size indicates the fraction of expressing cells and the colors represent normalized gene expression levels. C). Tissue prevalence estimated by Ro/e score in endothelial cells. D). Kaplan–Meier estimation of PFS time in NPC patients by Tip_SPARC subpopulation signature score. E). Crosstalk numbers between 8 malignant epithelial subtypes and 8 endothelial subtypes. F). Bar chart showing functional pathway enrichment analysis in Tip_SPARC subpopulation. G). Boxplot showing *SPARC* and *FTO* mRNA expression in each subpopulation of endothelial cells. H). MeRIP‐seq in HUVECs co‐cultured with malignant cells (HK1) showing the level of m6A enrichment in SPARC mRNA regions. I). RIP‐qPCR showing the direct interaction between FTO protein and SPARC mRNA. J). MeRIP‐qPCR assay showing relative m6A enrichment of SPARC in HUVEC cells in IgG control, HUVEC cultured alone, HUVEC cocultured with HK1 or SUNE2, and HUVEC with FTO knockdown (KD) cultured alone. K). RT‐qPCR showing relative mRNA levels for *FTO* and *SPARC* in HUVEC cells under HUVEC cultured alone (HUVEC1, HUEVC4), HUVEC cocultured with HK1 or SUNE2 (HUVEC2, HUEVC5) and HUVEC with FTO knockdown cultured alone (HUVEC3, HUEVC6). L). Western blot showing FTO and SPARC protein levels in HUVEC cells under various experimental groupings. The groupings are identical to that in (I). M). The tube formation assay showing tube‐forming ability of HUVECs under various experimental groupings. The groupings are identical to that in (I). N). TEER showing permeability of HUVECs under various experimental groupings. The groupings are identical to that in (I) (left panel). Bar plot showing comparison of the proportion of Claudin‐5^+^CD31^+^ /CD31^+^ cells in different groups (right panel). O). RT‐qPCR showing the effect of YTHDF2 knockdown on the mRNA expression level of *YTHDF2*, *FTO* and *SPARC*. P). RIP‐qPCR showing the direct interaction between YTHDF2 protein and SPARC mRNA. Q). RT‐qPCR showing the effect of FTO knockdown, combined FTO and YTHDF2 knockdown conditions on the mRNA expression level of *YTHDF2*, *FTO* and *SPARC*. R). Western blot showing the effect of FTO knockdown, combined FTO and YTHDF2 knockdown conditions on the protein expression level of YTHDF2, FTO and SPARC. Data in (J–L and N–R) are mean ± SD from 3 independent experiments, *p < 0.05, **p < 0.01 and ***p < 0.001 were determined by two‐way ANOVA with Bonferroni's post test.

We next explored the molecular mechanisms underlying FTO‐mediated endothelial disruption that may facilitate metastatic progression. Single‐cell omic analysis identified 8 endothelial subtypes in all samples, including tip cells, vein cells, lymphatic endothelial cells, and artery cells (**Figure** **5**A,B), and among these subtypes, 3 of them were enriched in primary NPC samples (Figure 5c; Figure S11A, Supporting Information). Notably, the Tip_SPARC subpopulation was characterized by its pronounced infiltration within the primary lesion but barely detectable in normal tissues (Figure 5C). Furthermore, patient samples with high Tip_SPARC signature expression were significantly correlated with shorter overall survival than those with low Tip_SPARC signature expression (Figure 5D; Table S3, Supporting Information). Cell to cell interaction analysis also showed a strong communication index between the Tip_SPARC subpopulation and NOTCH1^+^ cancer stem‐like cells (Figure 5E). Differential gene expression analysis identified SPARC, a gene encoding a cysteine‐rich acidic matrix‐associated protein, as one of the highly upregulated genes in tip cells (Figure S11B, Supporting Information). In contrast, CLDN5, a gene involved in tight junction formation, was significantly downregulated in these cells, suggesting a potentially reduced cell‐cell junction formation^[^ ^21^ ^]^ (Figure S11C, Supporting Information). SPARC^+^ tip cells exhibited upregulated pathway signatures involved in endothelial cell migration and proliferation, suggesting these cells may have reduced cell‐cell junctions accompanied by increased migratory capabilities (Figure 5F). Remarkably, the expression levels of *SPARC* and *FTO* were both upregulated in Tip_SPARC subpopulation by scRNA‐Seq analysis, pointing to the potential functional interactions between FTO and SPARC (Figure 5G). To test if SPARC might be a downstream target of FTO, MeRIP‐seq analysis was performed in endothelial cells co‐cultured with HK1 tumor cells. The result showed that there was a lowered m6A modification on SPARC mRNA 3′UTR in HUVEC cells co‐cultured with HK1 tumor cells as compared to HUVEC alone (Figure 5H; Figure S11D, Supporting Information). The SRAMP (sequence‐based m6A modification site predictor) algorithm was applied to identify m6A sites with high confidence in the 3′ UTR of *SPARC* mRNA (Figure S11E, Supporting Information). Importantly, RIP assays using anti‐FTO antibody in HUVEC validated that *SPARC* mRNA was significantly associated with FTO protein (Figure 5I; Figure S11F, Supporting Information). Furthermore, MeRIP‐qPCR was performed to detect the enrichment of m6A of *SPARC* in HUVEC under various conditions. Remarkably, the results demonstrated that the m6A modification of *SPARC* mRNA was significantly reduced in HUVEC co‐cultured with tumor cells, given the uptake of tumor‐derived FTO protein by HUVEC. (Figure 5J). Conversely, a significant elevation of m6A modifications of *SPARC* was observed in HUVEC cells with FTO knockdown, confirming *SPARC* was a direct target of FTO in HUVEC (Figure 5J). The effect of FTO on *SPARC* m6A modifications appeared to affect *SPARC* mRNA since the elevation of FTO in co‐cultured HUVEC cells with HK1 tumor cell line led to a notable upregulation of SPARC expression (Figure 5K,L). Conversely, the knockdown of FTO in HUVEC resulted in a significant downregulation of SPARC expression at both mRNA and protein levels (Figure 5K,L). Functionally, while co‐culturing HUVEC with HK1 tumor cells resulted in a significant reduction in the tube formation capacity, impaired TEER, and the decreased CLDN5 expression in endothelial cells, knockdown of FTO markedly rescued endothelial dysfunction compared to the NC group, emphasizing the critical role of FTO in disrupting endothelial integrity and function (Figure 5M,N; Figure S12A,B, Supporting Information).

m6A “readers” were fundamentally involved in the recognition of methylated target mRNA.^[^ ^11^ ^]^ YTHDF2 (YTH domain family 2) is the most effective m^[^ ^6^ ^]^A reader that weakens mRNA stability by recognizing and distributing m^[^ ^6^ ^]^A‐containing mRNAs to processing bodies.^[^ ^22^ ^]^ Given the positive correlation between the “eraser” FTO and SPARC expression, we hypothesized that YTHDF2 may function as a “reader” that negatively regulates translational efficiency and the degradation of m6A modified SPARC. The knockdown of FTO did not influence the expression levels of YTHDF2 in HUVEC by qPCR and immunoblotting (Figure S13A,B, Supporting Information). However, *YTHDF2* knockdown substantially increased *SPARC* expression but not *FTO* expression in mRNA level (Figure 5O). The interaction between YTHDF2 protein and *SPARC* mRNA was also verified by RIP assays in HUVEC (Figure 5P; Figure S13C, Supporting Information). Importantly, the knockdown of YTHDF2 could counteract the degradation of SPARC caused by FTO knockdown in mRNA and protein level (Figure 5Q,R). We also performed mIF staining to assess FTO, YTHDF2, and SPARC expression in the control group, FTO‐KD group, YTHDF2‐KD group, and FTO/YTHDF2 double knockdown group. SPARC protein was significantly decreased in cells with FTO depletion, which was restored in cells with FTO/YTHDF2 double knockdown (Figure S13D, Supporting Information). SPARC ELISA analysis of culture medium demonstrated similar results, suggesting the regulation of FTO on SPRAC mRNA m6A modification was YTHDF2‐dependent (Figure S13E, Supporting Information). Taken together, these results suggested that SPARC was a functionally important m6A target of FTO/YTHDF2.

### 2.7 SPARC Promotes Disruption of Inter‐Endothelial Cell‐Cell Junctions

**Figure 6:**
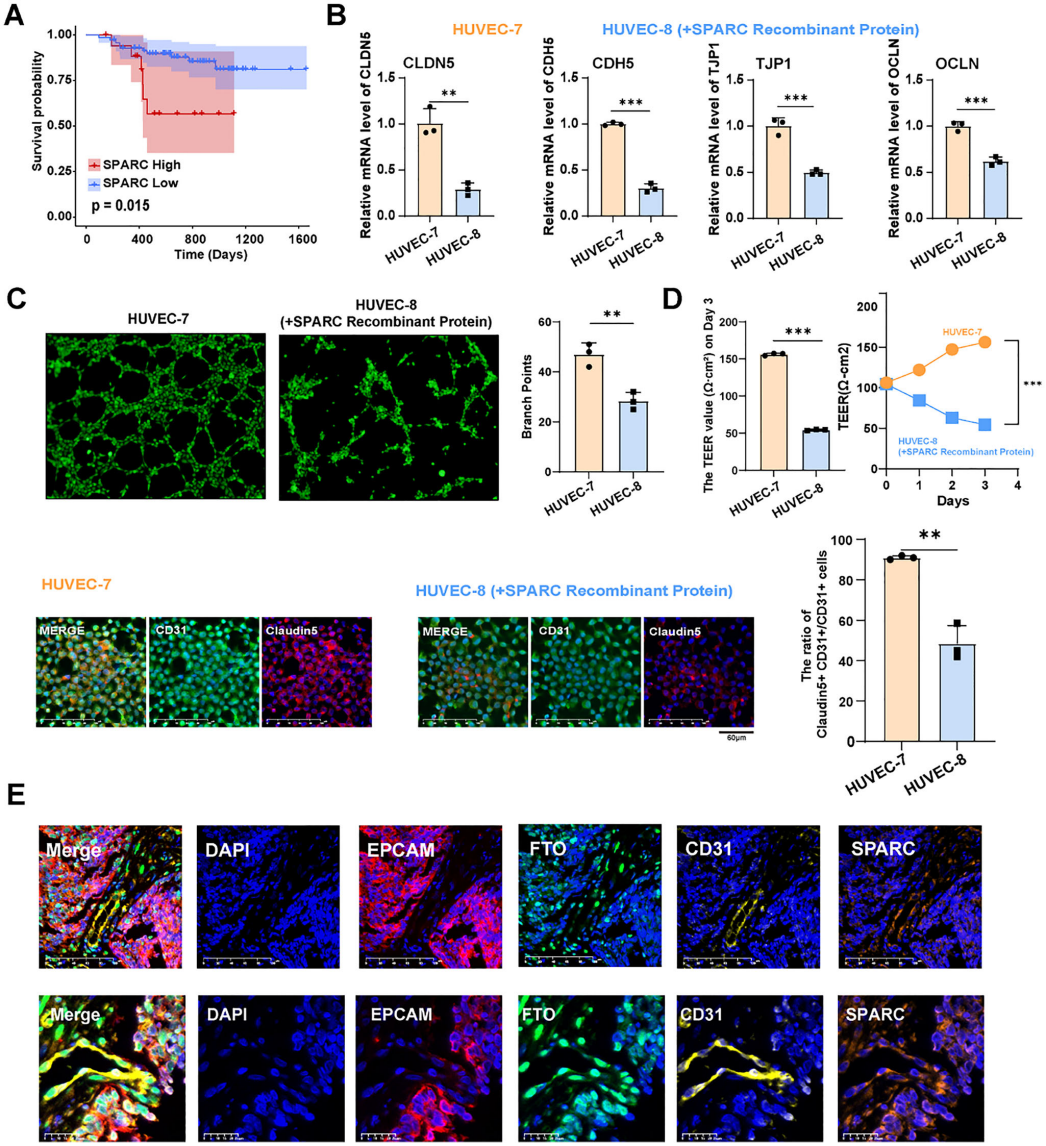
SPARC protein promotes the disruption of endothelial barriers. A). Kaplan‐Meier curve demonstrates that patients with high SPARC expression have a poorer prognosis. B). RT‐qPCR showing the effect of SPARC recombinant protein treatment on the mRNA expression level of *CLDN5*, *CDH5*, *TJP1* and *OCLN* in HUVEC. C). The tube formation assay showing tube‐forming ability of HUVECs under HUVEC (HUVEC7) and HUVEC treated with the SPARC recombinant protein (HUVEC8). D). The values of trans‐endothelial electrical resistance (TEER) showing permeability of HUVECs under HUVEC (HUVEC7) and HUVEC treated with the SPARC recombinant protein (HUVEC8). The statistical results of TEER are shown on the top. Immunofluorescence showing CD31 and Claudin‐5 expression in HUVEC‐7 and HUVEC‐8 cells (bottom panel). Bar plot comparing the proportion of Claudin‐5^+^CD31^+^ /CD31^+^ cells in different groups (bottom panel). E). mIHC showing the expression of FTO (green), EPCAM (red), CD31 (yellow), and SPARC (orange) in NPC tissues. Data in (B–D) are mean ± SD from 3 independent experiments, *p < 0.05, **p < 0.01 and ***p < 0.001 were determined by two‐way ANOVA with Bonferroni's post test.

SPARC has been reported to promote disruption of cell‐cell junctions, in both HUVEC and human cerebral microvascular endothelial cells (hCMEC/D3).^[^ ^23^, ^24^, ^25^ ^]^ High SPARC expression was significantly associated with poor survival in NPC patients (**Figure** **6**A). Interestingly, the supplementation of SPARC recombinant protein to the HUVEC cells significantly downregulated the expression of tight junction genes, including *CLDN5*, *CDH5*, *TJP1*, and *OCLN*, and impaired the endothelial cell tube‐forming ability, TEER, and CLDN5 expression in endothelial cells (Figure 6B–D). Importantly, the knockdown of SPARC partially rescued endothelial dysfunction induced by FTO overexpression in HUVEC, as assessed by the endothelial cell tube‐forming ability, TEER, and CLDN5 positivity (Figure S14A–D, Supporting Information). Finally, mIHC assay confirmed the presence of FTO^+^ EPCAM^+^ malignant cells in close proximity to FTO^+^ CD31^+^ SPARC^+^ endothelial cells in clinical NPC patient samples (Figure 6E; Figure S14E, Supporting Information). Taken together, these results suggested that SPARC protein served as a downstream effector of FTO and promoted the degradation of tight junction proteins, resulting in endothelial disruption and vascular abnormality.

To assess if endothelial SPARC mediates the effects of junctional disruption induced by FTO‐containing exosomes, we performed SPARC knockdown (KD) experiment in HUVECs incubated with FTO‐containing exosomes from HK1 and SUNE2 tumor cell lines (Figure S15A,B, Supporting Information). Remarkably, knockdown of SPARC in HUVECs significantly rescued the endothelial disruption induced by FTO‐containing exosomes as compared to control HUVECs, as evidenced by the restoration of in vitro tube formation capability, TEER, and endothelial junction marker Claudin5 expression. These data suggested a causal role of endothelial SPARC on junctional disruption due to the exosomal uptake of FTO in the endothelial cells (Figure S15C–E, Supporting Information).

## 3 Discussion

Cancer stem‐like cells (CSCs) are considered a key subpopulation of heterogeneous tumor cells that are capable of self‐renewal, tumor initiation, metastatic dissemination, and are highly resistant to multiple therapies.^[^ ^26^, ^27^ ^]^ Given their central role in tumor progression, CSCs represent attractive targets for the development of novel anti‐tumor therapies.^[^ ^28^, ^29^ ^]^ It is reported NPC CSCs often express markers including CD44, CD133, ALDH1, and classical stemness transcription factors with the activation of stem cell‐related pathways such as NOTCH and Hedgehog.^[^ ^30^, ^31^, ^32^ ^]^ In one study, the authors showed that a subset of NPC cells co‐express elevated NOTCH1 pathway and classical stemness markers, including SOX2 and OCT4.^[^ ^33^ ^]^ Furthermore, cancer cells with NOTCH3 activation appear to be resistant to multiple drug treatments and are required for sphere formation and tumorigenicity in several cancers.^[^ ^34^, ^35^ ^]^ Another NOTCH family member, NOTCH4, has been shown to be enriched in CSCs, and its depletion affects stemness and tumorigenicity in NPC.^[^ ^31^ ^]^ NOTCH pathway activation has been associated with metastasis in various cancer types.^[^ ^36^, ^37^, ^38^ ^]^ Nevertheless, how NOTCH1 promotes CSC stemness and metastatic progression in NPC remains to be clarified. In the present study, we have applied an integrative analysis of scRNA‐seq and spatial omics to explore molecular mechanisms regulating tumor‐endothelial interactions. We revealed the presence of NOTCH1^+^ cancer stem‐like primary tumor subclones with substantially enhanced stemness, invasiveness, and tumorigenic potential. These NOTCH1^+^ CSCs are poorly differentiated and evolutionally similar to the patient‐matched liver metastatic clones, implicating that they may be the pre‐metastatic subclone giving rise to distant metastasis (Figure 1F–H). Consistent with our hypothesis, NOTCH1^+^ CSC‐CTCs, marked by EPCAM^+^ EBNA1^+^ NOTCH1^+^ CD45^−^, were detected in NPC patient blood samples (Figure 1N). Strikingly, both the number and proportion of these NOTCH1^+^ CSC‐CTCs were significantly elevated in the metastatic patient group compared to the non‐metastatic cases, suggesting a strong clinical relevance (Figure 1N).

The search for single CTCs with high stemness has been challenging, given the fact that most of the isolated CTCs are non‐proliferative and apoptotic due to various stress factors present in the blood stream.^[^ ^39^ ^]^ It has been clearly demonstrated that CTC clusters, rather than single CTCs, express high stemness signatures due to DNA hypomethylation on binding sites for OCT4, NANOG, SOX2, and SIN3A.^[^ ^40^ ^]^ This finding is consistent with the observations that CTC clusters often possess significantly higher proliferative and metastatic potential.^[^ ^41^, ^42^, ^43^ ^]^ While our CTC isolation platform was not designed for the specific enrichment of CTC clusters, it is plausible that NOTCH1^+^ CSC‐CTC clusters may be detected in NPC patients, which warrants further investigations. Nevertheless, the detection and characterization of these NOTCH1^+^ CSC‐CTCs may pave the way for mechanistic studies on NPC metastasis and could serve as noninvasive biomarkers for real‐time monitoring of disease progression and therapeutic response.

In recent decades, m6A methylation has emerged as one of the most prevalent RNA modifications regulating various fundamental biological processes, and FTO is recognized as a key m6A demethylase involved in this process.^[^ ^10^, ^44^, ^45^ ^]^ In NPC, FTO has been shown to play critical roles in disease progression and therapeutic resistance. A study has shown that FTO can enhance radio resistance by reducing the m6A modification of OTUB1.^[^ ^46^ ^]^ Another work found that FTO cooperates with ALKBH5 to attenuate ARHGAP35 mRNA and protein and promote malignant progression of NPC.^[^ ^14^ ^]^ These investigations indicate that the increased FTO activity and reduced m6A modification could be beneficial for NPC growth. Our study has identified FTO as a direct transcriptional target of NOTCH1. This is achieved by the nuclear translocation and the binding of NICD to the promoter region of FTO, which robustly induced FTO transcription (Figure 2). Knockdown of NOTCH1 significantly suppressed FTO expression and resulted in reduced tumor stemness, invasiveness, and tumorigenic potential (Figures 1O–Q and 2F–G), while FTO‐OE significantly accelerated metastatic progression (Figure 4). Therefore, FTO was likely a key downstream target of NOTCH1 involved in cancer metastasis.

Classically, FTO mediates m6A demethylation function within the nucleus of most cells, and it can also localize to the cytoplasm to promote both m6A and cap m6Am demethylation in polyadenylated RNA.^[^ ^47^ ^]^ Limited research has indicated that the FTO protein can be transferred between cells via exosomes. In one study, gefitinib‐resistant cells derived FTO‐containing exosomes could decrease gefitinib sensitivity of recipient cells through FTO/YTHDF2/ABCC10 axis in advanced or metastatic non–small cell lung cancer.^[^ ^48^ ^]^ Another study demonstrated that exosomal FTO derived from neural stem cells targets microglia and inhibits the m6A modification of NRF2, which alleviates nerve injury caused by microglial inflammatory response.^[^ ^49^ ^]^ To our knowledge, tumors secrete exosomal FTO to impact m6A modifications in surrounding tumor‐associated endothelial cells have not been reported. Hence, our work has uncovered for the first time that the NOTCH1^+^ tumors can secrete a large number of exosomes carrying FTO proteins, which were efficiently taken up by neighboring endothelial cells (Figure 3). Intriguingly, FTO transfer appeared to occur preferentially from tumor to endothelial cells. It is tempting to speculate that the direct release of FTO proteins to the microenvironment may represent an efficient and powerful means for cancer cells to educate the tumor microenvironment. Without much engagement of transcriptional and translational machinery to express FTO, which could be high energy and time‐consuming, the direct delivery of functional FTO proteins via exosomes can achieve rapid RNA modifications within the recipient cells without much delay. Such instant and efficient molecular crosstalk between tumors and endothelial cells may allow efficient vascular remodeling to take place for the successful completion of metastatic dissemination.

The intravasation and extravasation of tumor cells during metastatic progression often induce vascular remodeling.^[^ ^50^, ^51^, ^52^ ^]^ Endothelial cells within the vasculature function as a semi‐selective barrier between peripheral organs and the blood circulatory system. Cell‐cell interactions are frequently mediated by tight junctions, adhesive junctions, and gap junctions.^[^ ^53^, ^54^, ^55^ ^]^ The degradation of the vascular basement membrane and the endothelial barrier can substantially enhance vascular permeability, a mechanism often explored by the intravasating and extravasating cancer cells. This process represents a critical rate‐limiting step in tumor hematogenous spread.^[^ ^56^ ^]^ Our work highlighted a delicate mechanism mediated by exosomal FTO that contributes to the disruption of tight junctions in endothelial cells, leading to increased vascular permeability. Molecularly, SPARC was identified to be a major effector targeted by FTO and YTHDF2. The accumulation of FTO in endothelial cells reduced the degradation of the SPARC mRNA in an YTHDF2‐dependent manner (Figure 5). The elevated SPARC protein activity in endothelial cells induced degradation of tight junction genes and endothelial dysfunction (Figure 6). In recent years, SPARC has been identified as a key factor involved in the disruption of cell‐cell junctions and affects endothelial cell function.^[^ ^23^, ^24^, ^25^ ^]^ Therefore, to achieve efficient vascular remodeling and metastatic dissemination, NOTCH1^+^ CSCs may have developed an intricate mechanism to induce endothelial junction disruptions by direct tumor‐to‐endothelial transfer of FTO via exosomes. Due to the uptake of tumor‐derived FTO proteins in endothelial cells, SPARC m6A level can be rapidly reduced, resulting in its mRNA stabilization and enhanced SPARC protein activity. As a result, FTO served both as a messenger and executor during the communication between tumor and endothelial cells, enabling endothelial‐specific elevation of SPARC proteins that can be secreted locally with high concentration to degrade inter‐endothelial tight junction genes effectively.

We acknowledge several limitations in the present study. Due to the limited tissue availability in NPC biopsies, matched WES and Single‐cell RNA‐seq analysis could only be performed on one patient (P3). Studies with larger cohorts or optimized multi‐omics tissue allocation strategies are needed to validate these findings. While the tail vein injection model provided valuable insights into FTO's role in crosstalk between malignant cells and endothelial cells, this approach cannot fully recapitulate the complete metastatic cascade, especially the intravasation process. Future studies employing orthotopic implantation mouse models will be essential to precisely delineate FTO's function during tumor cell intravasation into the bloodstream. While our clinical analyses of NOTCH1^+^ CTCs and plasma FTO were statistically significant, future multi‐center studies with larger patient cohorts are necessary to strengthen the clinical utility.

**Figure 7:**
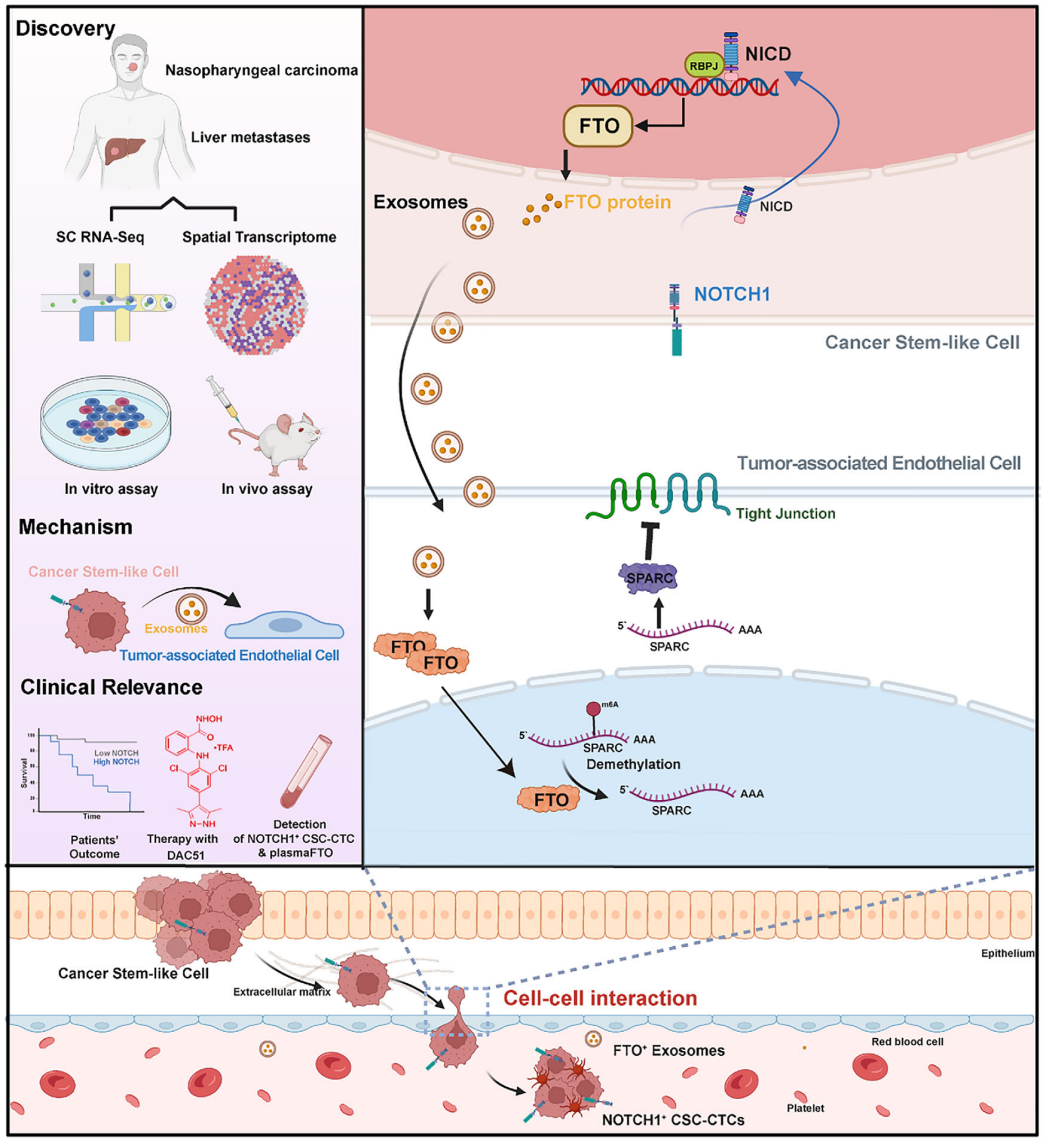
Schematic diagram of cross‐talks among cancer stem‐like cells and tumor‐assoicated endothelial cells in the TME of NPC. Integrated omics analysis of matched primary and liver metastatic NPC tumors reveals a unique NOTCH1^+^ CSC subpopulation exhibiting enhanced stemness properties and tumorigenic capacity. With in vitro and in vivo assays, exosomal transfer of tumor‐derived FTO from NOTCH1^+^ cells to the endothelium promotes vascular permeability and metastatic potential. The FTO‐YTHDF2‐SPARC axis in endothelial cells induces impairment of inter‐endothelial tight junctions due to reduced m6A modification that stabilizes SPARC mRNA and increases SPARC protein abundance. The FTO inhibitor DAC51, NOTCH1+ CSC‐CTCs and plasma FTO proteins show high therapeutic and diagnostic value for NPC distant metastasis patients. (Graph created with BioRender, https://www.biorender.com/).

In conclusion, comparative omic analyses of primary and metastatic NPC tumors revealed a distinct subset of NOTCH1^+^ CSCs characterized by heightened stemness, invasiveness, and tumorigenicity. NOTCH1^+^ tumor cells actively induced FTO mRNA expression and protein secretion via exosomes, which were efficiently taken up by the neighboring endothelial cells. This tumor‐to‐endothelial FTO transfer was crucial for vascular remodeling and metastasis, acting through the NOTCH1–FTO–SPARC axis to disrupt inter‐endothelial tight junctions. Importantly, using mouse models of metastasis, treatment with the FTO inhibitor DAC51 exhibited significant anti‐tumor effects. Moreover, both NOTCH1^+^ CSC‐CTCs and circulating plasma FTO protein were reliably detected and displayed markedly elevated levels in metastatic NPC patient blood samples as compared to non‐metastatic cases, providing potential liquid biopsy technologies for real‐time disease monitoring. Although larger cohorts of clinical samples are required to validate our findings, such unconventional, non‐cell‐autonomous function of FTO in driving vascular remodeling and facilitating metastatic progression may open new avenue for devising novel companion diagnostics and therapeutics for NPC diagnosis and treatment (**Figure** **7**).

## 4 Experimental Section

### Clinical Samples Collection

Fresh nasopharyngeal carcinoma specimens, along with paired adjacent non‐tumor tissues and fresh blood samples, were procured and consented from the Sun Yat‐sen University Cancer Center under item number B2024‐098. The classification of tumor‐node‐metastasis (TNM) was conducted according to the guidelines of the ninth edition of the TNM staging system. A cohort of 12 patients diagnosed with nasopharyngeal carcinoma provided written informed consent for participation in the study. The collection and utilization of these clinical specimens were sanctioned by the Sun Yat‐sen University Cancer Center's ethical review board. Relevant information regarding the samples is presented in Table S1 (Supporting Information).

Plasma samples were obtained from the Sun Yat‐sen University Cancer Center. The study was conducted in accordance with the Declaration of Helsinki and received approval from the Life Sciences Ethics Review Committee of Zhengzhou University. All participants provided written informed consent before the collection of whole blood. The samples (*n* = 20) were anonymized, with only gender, age, and pathological diagnosis recorded. Whole blood was collected in EDTA‐coated tubes, gently mixed, and subsequently centrifuged at 2000 × g for 10 min. Plasma was carefully extracted and stored at −80 °C until further analysis. Relevant information regarding the plasma samples is presented in Table S2 (Supporting Information).

### Cell Lines and Cell culture

Human umbilical vein endothelial cells (HUVECs) were purchased from Biospecies Co., Ltd. on November 25, 2023. Their authenticity was confirmed by short tandem repeat (STR) multiplex amplification, and the cell line was free from contamination. Additionally, one Epstein‐Barr virus (EBV)‐associated nasopharyngeal carcinoma (NPC) cell line (SUNE2) and one non‐EBV NPC cell line (HK1) were sourced from the Sun Yat‐sen University Cancer Center on September 2, 2022.Their authenticity was confirmed by short tandem repeat (STR) multiplex amplification, and the cell line was free from contamination. Specific information about cells is presented in Table S7 (Supporting Information). The SUNE2 and HK1 cell lines were cultured in RPMI‐1640 medium (Gibco, Canada) supplemented with 10% fetal bovine serum (Sigma, USA), while HUVECs were maintained in endothelial cell medium (ECM; ScienCell). All cell lines were incubated in a humidified atmosphere containing 5% CO2 at 37 °C. Indirect coculture assays were conducted using 0.4 µm cell culture inserts (Corning, NY, USA). List of cell sources in Table S4 (Supporting Information).

### CTCs Isolation

CTC were isolated from the peripheral blood of NPC patients using an inertial microfluidic approach based on a four‐stage spiral microfluidic device (Celutriate Chip 1).^[^ ^57^ ^]^ This antibody‐free enrichment system separates CTCs from whole blood by exploiting size‐dependent inertial and Dean drag forces, enabling the rapid depletion of hematopoietic cells and the efficient enrichment of larger target cells. Briefly, 10 mL of fresh peripheral blood was directly injected into the microfluidic device without any pre‐treatment steps, minimizing sample manipulation and preserving cell viability. The sample sequentially flowed through four spiral microchannel stages integrated with a lateral displacement design, which leverages hydrodynamic forces to guide cells based on their size. This configuration enabled efficient separation of larger CTCs from smaller blood components, including white and red blood cells. The entire enrichment process was completed within just 12 min, demonstrating high processing speed and throughput. According to the reported results, the system achieved a CTC recovery efficiency ranging from 52.3% to 65.8%, while simultaneously achieving over 99.95% depletion efficiency for white blood cells, thereby significantly improving the purity of the isolated CTC population.

Regarding the biomarkers for NPC‐derived circulating tumor cells, EBNA1 (Epstein‐Barr Virus Nuclear Antigen 1) is selected as a positive marker and CD45 as a negative marker in this study. EBNA1 (Epstein‐Barr Virus Nuclear Antigen 1) is one of the core proteins of Epstein‐Barr virus (EBV) that is expressed early during latent infection and remains persistently present. In NPC, particularly in non‐keratinizing undifferentiated carcinoma (WHO type II/III), nearly all tumor cells stably harbor the EBV genome and exist in a state of latent infection (Latency II).^[^ ^58^ ^]^ Under this latency program, EBNA1 is the only EBV nuclear antigen that is continuously expressed at high levels. Studies have shown that EBNA1 expression can be detected in almost all NPC tissues, whereas it is rarely expressed in normal nasopharyngeal epithelial cells, other types of tumors, or immune cells.^[^ ^59^ ^]^ Therefore, EBNA1 serves not only as a key marker of EBV infection but also as crucial molecular evidence for the origin of NPC cells. Given its high specificity and stable expression, EBNA1 was selected in this study as the representative biomarker for identifying CTCs derived from NPC. At the same time, to exclude contamination by white blood cells, CD45 was used as a pan‐leukocyte negative marker for identification.^[^ ^60^ ^]^

### Small Interfering (si) RNA and Plasmid Transfection

NOTCH1, FTO, SPARC, YTHDF2, and negative control (NC) siRNAs were synthesized by GenePharma (China) and the siRNAs sequences (5′ to 3′) are listed in Table S5 (Supporting Information). Knockdown plasmids of NOTCH1 and overexpression plasmids of FTO (Miaoling, China) were prepared by tagging the coding regions of NOTCH1 and FTO separately with Flag. For transient transfection, the indicated cells were transfected with siRNA or overexpression plasmids using Lipofectamine 3000 (Invitrogen, USA) according to the manufacturer's protocols and harvested 48 h after transfection.

### Construction of Stable Cell Lines

NOTCH1 knockdown stable NPC cells were established by infecting cells with lentivirus expressing the pLV3‐U6‐NOTCH1 (human)‐shRNA‐Fluc‐Puro. NOTCH1 overexpression stable NPC cells were established by infecting cells with lentivirus expressing the pLV3‐CMV‐NOTCH1 (human)‐EF1a‐Fluc‐Puro. FTO overexpression stable nasopharyngeal carcinoma cells were established by infecting cells with lentivirus expressing the pLV3‐CMV‐FTO (human)‐Fluc‐Puro. FTO knockdown stable NPC cells and HUVECs were established by infecting cells with lentivirus expressing the pLV3‐U6‐FTO (human)‐shRNA‐3×FLAG‐Fluc‐Puro. FTO overexpression stable NPC cells and HUVECs were established by infecting cells with lentivirus expressing the pLV3‐CMV‐FTO (human)‐3×FLAG‐Fluc–Puro.

### Indirect Coculture Systems

HUVECs (5 × 10^5^) per well in a 6‐well plate were cultured using ECM medium supplemented with 10% fetal bovine serum (FBS) and 1% penicillin/streptomycin. Concurrently, ≈5 × 10^5^ HK1 or SUNE2 cells were seeded into transwell inserts (pore size: 0.4 µm; diameter: 6.5 mm; Corning Incorporated, Corning, NY, USA) for a duration of 48 h. In this co‐culture system, HK1 or SUNE2 cells were introduced into the upper chamber, while HUVECs were cultured in the lower chamber.

### Western Blotting

RIPA lysis buffer (Beyotime, China) containing 1% protease and phosphatase inhibitors (Thermo Fisher Scientific, USA) was used to lyse cells. After sonication, protein concentration was measured using a BCA Protein Assay Kit (Beyotime, China). The samples were electrophoresed on 10% SDS‒PAGE gels and transferred onto PVDF membranes (0.45 µm; Merck‐Millipore, Germany). After blocking with QuickBlock Blocking Buffer (Beyotime, China) for 15 min at room temperature, the membranes were incubated at 4 °C overnight with primary antibodies, then incubated with HRP‐linked secondary antibodies for 1 h. Protein bands were detected by the enhanced chemiluminescence system (Millipore, MA, USA). Detailed information on relevant antibodies is provided in Table S6 (Supporting Information).

### Quantitative Real‐Time PCR

Quantitative real‐time PCR (qRT‐PCR) was used to determine the mRNA expression of key genes. Total RNA was extracted using the TRIzol reagent (Thermo Fisher, USA). qRT‐PCR was performed with the SYBR Green PCR kit (Thermo Fisher Scientific, USA) by CFX96 Touch Real‐Time PCR Detection System (Bio‐rad, USA), after cDNA was synthesized from total RNA using a PrimeScriptTM RT Kit (Takara, China). The relative gene expression was calculated using the 2‐ΔΔCT equation with GAPDH as an internal control. RT‐qPCR primer sequences are listed in Table S7 (Supporting Information).

### Cell Viability, Migration, and Invasion Assay

Cell viability was evaluated with Cell Counting Kit‐8 (CCK‐8; Beyotime, China). 1000 cells were seeded in a 96‐well plate, the absorbance was measured at a wavelength of 450 nm using a microplate reader (Bio‐Rad Laboratories, USA) at the indicated time.

For cell migration and invasion assay, 700 µL of culture medium with 10% FBS was added to a 24‐well plate, and then the 8µm pore transwell inserts were placed into each well. As for the invasion assay, the insert membranes were covered with 30% Matrigel (Corning, USA) in DMEM. Approximately 5 × 10^4^ cells in culture medium without FBS were added to the upper chamber and incubated. After incubation for 24 h, the medium was removed, and methanol was used to fix the cells. Crystal violet was then used to stain the cells. Images were acquired by an OLYMPUS microscope, and cells were counted using ImageJ software.

### Endothelial Barrier Permeability

The permeability of the endothelial barrier is assessed by quantifying trans endothelial electrical resistance (TEER). Human umbilical vein endothelial cells (HUVECs), seeded at a density of 5.0 × 10^4^ cells, were cultured on a Transwell polyester membrane cell culture insert, which was positioned within a 24‐well plate (pore size: 0.4 µm; diameter: 6.5 mm; Corning Incorporated, Corning, NY, USA). The TEER of the endothelial monolayer was measured using an electrical resistance meter (Millicell‐Electrical Resistance System, Millipore, USA). The formation of a stable endothelial monolayer was confirmed when the TEER values reached a consistent level. To examine changes in the endothelial cell monolayer, TEER values were subsequently monitored following various treatments.

### Multiplex Immunohistochemistry (mIHC) and Spatial Analysis

Multiplex Immunohistochemistry was performed using the PANO 7‐plex IHC kit (Panovue, Beijing, China). In brief, tissue slides were first incubated at 65 °C for 2 h to ensure proper adhesion, followed by deparaffinization with xylene and rehydration through a graded ethanol series (100%, 95%, 70%, 50%). The sections were then fixed in 10% neutral buffered formalin for 30 min. Antigen retrieval was carried out using EDTA buffer (pH = 9.0, ZSGB‐Bio, Beijing, China) in a microwave, after which the slides were blocked to minimize non‐specific binding. Primary antibodies were applied sequentially, followed by incubation with horseradish peroxidase (HRP)‐conjugated secondary antibodies. Tyramide signal amplification (TSA) was performed after each primary antibody application to enhance signal detection sensitivity. Subsequently, the sections were incubated with biotinylated rabbit polyclonal anti‐rabbit and rabbit anti‐mouse secondary antibodies. HRP‐conjugated streptavidin was then applied according to the manufacturer's instructions (Panovue, Beijing, China). For chromogenic reactions, biotinylated secondary antibodies were used in conjunction with streptavidin‐linked alkaline phosphatase, while streptavidin‐linked fluorophores were employed for immunofluorescence detection (excitation wavelengths: 480, 520, 570, 690, or 780 nm). Multispectral images were acquired using the Vectra Polaris Automated Quantitative Pathology Imaging System (Akoya Biosciences, Delaware, USA). Subsequent detection of positively stained cells and advanced spatial analysis were conducted using the HALO digital pathology platform (Indica Labs, Corrales, NM, USA). The multiplex immunohistochemistry (IHC) module facilitated the identification of FLAG‐tagged endothelial cells, characterized by the CD31 marker, and tumor epithelium cells, characterized by the EPCAM marker, through color deconvolution and nuclear segmentation of differentially stained cells. The results of the cell registration process were manually reviewed for all TMA cores, with false positive annotations and cores exhibiting technical artifacts being excluded from the analysis. The spatial analysis module was employed to assess the proximity between FLAG‐tagged endothelial cells and tumor epithelial cells, with measurements documented across a spectrum of distance intervals ranging from 0 to 100 µm. Following this, the mean number of FLAG‐tagged endothelial cells per tissue microarray (TMA) core located in the tumor center was calculated and utilized to construct a histogram.

### Matrigel Tube Formation Assay

For the in vitro tube formation assays, 48‐well culture plates were uniformly coated with 150 µL of Matrigel (Corning, NY, USA) per well, followed by a polymerization process lasting 30 min at 37 °C. Subsequently, 200 µL of HUVEC cells, at a concentration of 5 × 10^4^ cells, either alone or in combination with additional treatments, were seeded onto the polymerized Matrigel. Following an 8 h incubation period at 37 °C, each culture was imaged using a confocal microscope (Leica, Wetzlar, Germany), capturing four random fields per well.

### Transmission Electron Microscopy (TEM)

Following the vertexing of various HUVEC samples for 2 min, the samples were applied to a copper grid suitable for transmission electron microscopy (JEOL, Japan). Excess moisture was subsequently removed using filter paper. The samples were then imaged using the transmission electron microscope.

### Immunofluorescence Staining and Confocal Microscopy

Cells were fixed in 0.4% paraformaldehyde, permeabilized in 0.5% Triton X‐100, blocked in 1% BSA‐PBS, and incubated overnight at 4 °C with primary antibodies. Subsequently, the coverslips were incubated with HRP‐conjugated secondary antibodies for 30 min at room temperature, followed by tyramine signal amplification to stain the cells. The elution buffer was utilized to remove the antibodies for subsequent antibody incubation. Confocal dishes were sealed with Mounting Medium containing DAPI (abcam, USA) to prevent quenching, and fluorescence images were captured using an OLYMPUS FV1000 (OLYMPUS, Japan) confocal scanning microscope. The antibodies used for immunofluorescence are listed in Table S6 (Supporting Information).

### Methylated RIP Analysis

Total RNA was extracted from nasopharyngeal carcinoma cells using TRIzol. Subsequently, methylated RNA immunoprecipitation (RIP) was performed utilizing the Magna MeRIP m6A Kit (RiboBio, Guangzhou, China) in accordance with the manufacturer's protocol. Following this, an RNA purification kit (Zymo Research Corp., Irvine, CA, USA) was employed to isolate the enriched RNA. The enrichment of m6A‐containing RNA was assessed relative to the negative control (IgG) sample using quantitative reverse transcription polymerase chain reaction (qRT‐PCR). MeRIP‐PCR primer sequences are listed in Table S7 (Supporting Information).

### RNA Immunoprecipitation Analysis

RNA immunoprecipitation (RIP) was conducted using antibodies with specificity, employing the Magna RIP RNA‐Binding Protein Immunoprecipitation Kit (Merck‐Millipore, Darmstadt, Germany) in accordance with the manufacturer's instructions. Specifically, 4 × 10^7^ nasopharyngeal carcinoma cells were collected and lysed using RIP lysis buffer. Following centrifugation at 4 °C, the supernatant was incubated with specific antibodies and negative control IgG at room temperature. Subsequently, the bead‐antibody complex underwent washing and incubation with Proteinase K buffer. The input and immunoprecipitated RNAs were extracted using TRIzol reagent and reverse transcribed into complementary DNA (cDNA) utilizing the RevertAid First Strand cDNA Synthesis Kit (Thermo Fisher Scientific, MA, USA). Quantitative reverse transcription polymerase chain reaction (qRT‐PCR) was employed to assess the fold enrichment relative to the negative control (IgG) sample. RIP‐PCR primer sequences are listed in Table S7 (Supporting Information).

### m6A Quantification by Dot Blot

A total of 600 ng of RNA was employed for m6A quantification via dot blot assays. In summary, RNA samples were applied onto a nylon membrane using a dot blot apparatus and subjected to aspiration for 15 min, followed by UV fixation at 130 J cm^−^^2^ (254 nm). The membranes were subsequently stained with 0.04% methylene blue in conjunction with 0.5 m sodium acetate to visualize the total RNA. Before antibody incubation, the membranes were washed with TBS‐Tween 0.1% and blocked for 1 h at room temperature using 5% nonfat milk in 0.1% TBS‐Tween. An anti‐m6A primary antibody was employed to detect m6A methylation on the RNA during an overnight incubation at 4 °C (refer to Table S6, Supporting Information for the list of antibodies utilized in this study). Following membrane washing with 0.1% TBS‐Tween, secondary antibodies conjugated with horseradish peroxidase were applied. The m6A‐methylated RNA was visualized using an enhanced chemiluminescence Western blotting substrate (Pierce, Waltham, MA, USA). The labeling of m6A‐methylated RNA was normalized to the total RNA, as indicated by the methylene blue‐stained membrane.

### Chromatin Immunoprecipitation (ChIP)

Chromatin‐Immunoprecipitation (ChIP) assays were conducted in HK1 and SUNE2 cells using the SimpleChIP Enzymatic Chromatin IP Kit (CST, USA). The procedure followed the manufacturer's instructions, with NOTCH1, bound chromatin being immunoprecipitated using 4 µg of NOTCH1 and normal rabbit IgG antibody per 5 × 10^6^ cells. ChIP‐qPCR was then performed on inputs and recovered purified ChIP DNA to evaluate the expression of the FTO promoter, with primer sequences available in Table S7 (Supporting Information). Fold enrichment was calculated as 2(Ct (FTO)‐Ct (IgG)).

### Exosome Isolation and Identification

Exosomes were isolated through a sequential ultracentrifugation protocol. Initially, the cell culture supernatant derived from HK1 and SUNE2 cell lines was centrifuged at 500 g for 10 min to remove precipitates. The resultant supernatant was then subjected to centrifugation at 2000 g for 10 min at 4 °C to eliminate cellular debris. The clarified supernatant was subsequently filtered through a 0.22 um membrane and further centrifuged at 10 0000 g for 4 h in an ultracentrifuge tube. The pellet obtained was resuspended in phosphate‐buffered saline (PBS) and underwent an additional centrifugation at 10 0000 g for 70 min. The final pellet obtained from this procedure was identified as exosomes. Transmission electron microscopy (TEM) was employed to examine exosome morphology. Exosome surface markers were characterized via Western blot analysis, utilizing antibodies at a dilution of 1:1000 (Abcam Inc.) to assess exosome‐specific marker proteins, Calnexin and CD63. The particle size and concentration of the exosomes were analyzed through nanoparticle tracking analysis (NTA) using the NanoSight NS300 (Malvern).

### Enzyme‐Linked Immunosorbent Assay (ELISA)

The ELISA was performed following the manufacturer's instructions provided with the ELISA kit (Nanjing BYabscience technology Co.,Ltd). Briefly, plasma samples diluted 1:1000, along with biotinylated antibodies and streptavidin‐HRP, were added to 96‐well plates that had been pre‐coated with the appropriate antibody. The plates were incubated at 37 °C. After 60 min, the liquid was removed, and the wells were thoroughly washed. Subsequently, a chromogen solution was added to each well, and the plates were incubated for 15 min at 37 °C, protected from light. Finally, a stop solution was added to each well, and the absorbance was measured at 450 nm. A control group was included, which did not contain plasma. Each sample was analyzed in triplicate to ensure accuracy and reproducibility.

### In Vivo Tumorigenesis and Metastasis Assays

All mouse experiments conducted in this study received approval from the Institutional Animal Care and Use Committee of Sun Yat‐Sen University. BALB/c nude mice (aged 4–6 weeks, SPF level, 18–20 g) and NCG nude mice (aged 4–6 weeks, SPF level, 20–22 g) were obtained from the Guangdong Medical Laboratory Animal Center, Guangdong, China. The xenograft experiments were organized into two groups: a control group and a NOTCH1 knockdown group. NPC xenograft models were established using HK1 cells and NOTCH1 KD HK1 cells. Specifically, 1 × 10^7^ HK1 cells were subcutaneously injected into the right flank of 6‐week‐old BALB/c nude mice. Female BALB/c nude mice, aged 6 weeks and weighing between 14 and 18 g, were used. Tumor dimensions, including length and width, were measured every three days, and tumor volumes were calculated using the formula: Tumor volume = (length × width^2^)/2. In the tail vein metastasis model, the experiment was categorized into three distinct groups: the control group, the FTO overexpression group, and the FTO overexpression combined with DAC51 inhibitor (Selleck,S9876) treatment group. A total of 1 × 10^6^ HK1 cells or FTOOE HK1 cells were administered via tail vein injection into NCG mice. Weekly in vivo imaging of lung and liver metastases was conducted post‐inoculation utilizing a small animal fluorescence imaging system. Liver and lung tissues were excised and preserved in 4% phosphate‐buffered neutral formalin. Subsequent analysis of lung tissues was carried out using hematoxylin and eosin (HE) staining, multiplex immunohistochemistry (mIHC), and flow cytometry.

### Flow Cytometry Analysis

The study involved flow cytometry analysis of tumor tissues derived from liver and lung metastases using the tail vein injection model. Isolated lung and liver tissues were maintained in a cold DMEM medium supplemented with 5% fetal bovine serum (FBS), 1% GlutaMAX, and 1% penicillin–streptomycin, followed by mechanical mincing. Subsequently, the samples underwent enzymatic digestion at 37 °C for 30 min with a cocktail containing 2 mg mL^−1^ collagenase IV, 0.2 mg mL^−1^ dispase, and 0.1 mg mL^−1^ DNase I in the supplemented DMEM. The resulting cell suspension was homogenized by passing it through 40 µm cell strainers, utilizing 10 mL of supplemented DMEM per sample. To remove myelin, the suspension was combined with 90% isotonic Percoll diluted in 10× PBS and centrifuged for 20 min at 800×g. The cell pellet was resuspended in a cold buffer (MACS BSA diluted in autoMACS rinsing solution) and subjected to two rounds of centrifugation at 4 °C for 5 min at 300 g, with resuspension in cold buffer and flow cytometry staining buffer, respectively, to achieve a concentration of 10 00000 cells/100 µL per sample. Finally, the samples were incubated for 15 min at 4 °C with the following monoclonal antibodies: anti‐human EPCAM (Miltenyi Biotech, Cat#130‐110‐802, RRID: AB_2658222) and anti‐FLAG (Miltenyi Biotech, Cat#130‐113‐803, RRID: AB_2819369).In all experiments, viobility fixable dye (Miltenyi Biotech, Cat#130‐109‐816) was used to label dead cells. Data were collected on a MACSQuant Analyzer (Miltenyi Biotech) and analyzed using Flowlogic software (Miltenyi Biotech).

### In Vivo Subcutaneous Matrigel Plug

The subcutaneous Matrigel plug mouse model was established using female nude mice aged 8 to 10 weeks. A suspension containing either 1 × 10^6^ HUVECs alone or in combination with 1 × 10^6^ HK1 cells was prepared in Matrigel plugs. These plugs were composed of 0.8 mL Matrigel (BD Biosciences), supplemented with basic fibroblast growth factor (250 ng mL^−1^; R&D Systems) and heparin (60 U mL^−1^; Hospira Inc.), and subsequently implanted subcutaneously. After a 14‐day incubation period, the Matrigel plugs were excised, embedded in paraffin, and sectioned for analysis. Angiogenesis was assessed through immunohistochemical detection using an anti‐human CD31 antibody (eBioscience).

### Tail Vein Injection Mouse Model

Eight‐week‐old male NCG mice were intravenously injected with 1 × 10^6^ cancer cells. In the inhibitor group, HK1 cells were treated with Dac51 (Selleck, #S9876) at a dosage of 7.5 mg kg^−1^. The drug was administered via intraperitoneal injection starting three weeks after the initial tail vein injection, with subsequent injections given every two days until the experimental endpoint. Tumor development and growth were monitored using bioluminescence imaging following the administration of luciferin (Product #P1043, prepared at a concentration of ≈30.30 mg mL^−1^, with an injection volume of 100 µL).

### Dac51 Drug Testing Experiment

Dac51 was initially dissolved in dimethyl sulfoxide (DMSO) to achieve a final concentration of 5%. Subsequently, the solution was diluted with corn oil to prepare the desired dosages for administration. Eight‐week‐old male NCG mice were randomly assigned to four treatment groups, each consisting of five mice, based on the dosage levels: a control group receiving only corn oil, a low‐dose group (5 mg kg^−1^), a medium‐dose group (7.5 mg kg^−1^), and a high‐dose group (20 mg kg^−1^). Each group received an intraperitoneal injection of 50 µL of the prepared drug solution every two days until the experimental endpoint. To ensure the safety and tolerability of the treatment regimen, the body weight of the mice was closely monitored every three days throughout the study.

### Sample Preparation and scRNA Sequencing

10x Genomics Cell Preparation Guide describes best practices and general protocols for washing, counting, and concentrating cells from both abundant and limited cell suspensions (greater than or less than 100000 total cells, respectively) in preparation for use in 10x Genomics Single Cell Protocols. Cell viability was measured with the Acridine Orange/Propidium Iodide (AO/PI) kit. scRNA‐seq libraries were prepared using ChromiumTM Single Cell G Chip and Chromium Single Cell 5′ Library & Gel Bead Kit v2, and sequencing was accomplished on an Illumina NovaSeq6000 System using a paired‐end 150 bp.

### Single‐cell RNA Sequencing Data Analysis

The Cell Ranger Single‐Cell toolkit (v7.2.0) was applied to align reads for this case based on the human reference genome GRCh38. Single‐cell downstream analysis was based on the Seurat R package.^[^ ^61^ ^]^ Further quality control was applied to cells based on the following thresholds: 1) a count of expressed genes exceeding 150 but not surpassing 6000; 2) cells containing mitochondrial RNA content lower than 10%. The DoubletFinder R package was used to remove potential doublets.^[^ ^62^ ^]^ The gene expression data was then processed by normalizing and scaling each sample's filtered gene expression matrix using the functions, “NormalizeData” and “ScaleData” in the Seurat package. Batch effects across this case and other tissues were harmonized and the gene expression matrices from all samples were integrated using the Harmony R package.^[^ ^63^ ^]^ Finally, 36601 genes were identified. Principal component analysis (PCA) was performed on the corrected expression matrix using highly variable genes (HVGs) identified by the “FindVariableGenes” function. The most representative principal components were used to determine different cell types with the “FindCluster” function. The cell types were annotated, and 8 clusters were identified based on expression of the following marker genes: *CD19* and *MS4A1* for B cells, *PECAM1* and *VWF* for endothelial cells, *KRT8* and *KRT18* for epithelial cells, *COL1A1* and *COL6A1* for fibroblasts, *MS4A2* and *CPA3* for mast cells, *CD14* and *FCGR3A* for myeloid cells, *TNFRSF17* and *IGHG1* for plasma B cells, and *CD3D* and *CD3E* for T cells.

### Differential Expression Analysis Between Cell Types

To identify differentially expressed genes for each cell subtype, the “FindAllMarkers” functions from the Seurat package were used with default parameters.^[^ ^61^ ^]^ The expression differences with *P* < 0.05 and log2(fold change) > 0.3 were considered differentially expressed genes.

### Copy Number Variation (CNV) Calling From Single Cell RNA Sequencing

Numbat is a computational method as used to jointly reconstruct the subclonal phylogeny and single cell copy number profile of the patient P3, and to classify the single cells into six genotypes.^[^ ^64^ ^]^ Next, the inferCNV R package (inferCNV of the Trinity CTAT Project, provided at https://github.com/broadinstitute/inferCNV) is used to infer the large‐scale chromosomal copy number variations of each cell and verify benign cells and malignant cells. The other parameters were set to defaults. Each chromosome amplification or deletion is defined as a CNV event, and the sum of all CNV events determines the CNV score for each cell.

### Cell Developmental Trajectory Analysis

RNA velocity analysis was performed utilizing velocyto^[^ ^65^ ^]^ and SCvelo.^[^ ^66^ ^]^ The 10 × velocyto pipeline was implemented to quantify both spliced and unspliced reads for each sample derived from BAM files generated by Cell Ranger. To infer the root and terminal states of the underlying Markov process, the corresponding velocyto.R function was applied. Additionally, the Python package PAGA^[^ ^67^ ^]^ was employed to validate the pseudotime trajectories among the various epithelial cell subtypes. The single‐cell trajectory analysis of malignant cell subtypes was performed with R‐package LandSCENT^[^ ^68^ ^]^ using default parameters to get the computation of signaling entropy.

### Tissue Preference of Each Cell Subtype

The ratio of observed to expected cell counts (Ro/e) were computed for each cell subpopulation to assess the preferential distribution of these subpopulations across various tissues, as previously outlined.^[^ ^69^ ^]^ In summary, the expected cell counts for each subpopulation within each tissue were derived from the Chi‐square analysis, with a Ro/e value greater than 1 indicating a preferential presence of that cell subpopulation in the corresponding tissue.

### Genomic Instability Estimation

To estimate the genomic instability of each malignant cell, the genomicInstability R package was used, which uses the aREA algorithm to quantify the enrichment of sets of contiguous genes (loci‐blocks), on the gene expression profiles to estimate the Genomic Instability Score (GIS) for each analyzed cell.

### Epithelial Cell Characterization

Eight epithelial cell subtypes were identified and annotated based on expression of some markers, including a subpopulation with ciliated cells characterization (CAPS, TPPP3, and TMEM190).^[^ ^70^ ^]^ The CytoTRACE algorithm^[^ ^16^ ^]^ was used for predicting the differentiation status of all epithelial cell subpopulations. CytoTRACE scores ranged from 0 to 1 with high score indicating low differentiation status and a low score indicating high differentiation.

### Pathway Enrichment Analysis

The Cytoscape plugin ClueGO^[^ ^71^ ^]^ was used to analyze genes exhibiting logFC >0.3 and *p*‐value <0.05 within the Epi_NOTCH1 subpopulation. ClueGO facilitated a comprehensive biological analysis of these genes. The KEGG pathway set as the targeted option was selected. A *p*‐value threshold of less than 0.05 was established to denote statistical significance. For Epi_nOTCH1 and Endo_SPARC subpopulations, gene enrichment was performed using the enrichGO function in the R package “clusterProfiler v4.0^[^ ^72^ ^]^” for GO analysis.

### Whole Exome Sequencing (WES) Data Processing

For P3 patients, the WES data were aligned by BWA^[^ ^73^ ^]^ mem software with the genome reference hg38, and then duplicates were removed using sambamba^[^ ^74^ ^]^ markdup, realign, and recal with GATK^[^ ^75^ ^]^ RealignerTargetCreator, IndelRealigner, BaseRecalibrator tools, call somatical variation with Mutect2, and variation filter with GATK FilterMutectCalls and SelectVariants tools. Somatic copy number variants (SCNVs) were called using Control‐FREEC v11.1.^[^ ^76^ ^]^

### Spatial Transcriptomic Sample Preparation and Analysis

Spatial transcriptomic sample preparation and analysis involved the sectioning of paraffin embedded tissue samples into 10 µm thick slides, which were subsequently affixed to the capture regions (6.5 × 6.5 mm) of the Visium Spatial Tissue Optimization Slide (10 × Genomics). Following fixation and permeabilization, the released mRNA was captured by oligonucleotides. cDNA synthesis was conducted using a master mixture containing reverse transcription reagents and fluorescently labeled nucleotides. The cDNA was then covalently linked to the oligonucleotides and retained on the Visium slide upon removal of the tissue. A sequencing library was constructed post these procedures, and sequencing was performed using the Illumina NovaSeq 6000 System. The Spaceranger software from 10 × Genomics was employed to process the fastq files and spatial transcriptomics images for each sample, utilizing the human reference genome GRCh38. To elucidate the spatial organization of specific cell subtypes, the Seurat R package was utilized to integrate spatial and single‐cell data. Raw UMI counts were normalized using the “SCTransform” function. Dimensionality reduction and clustering were executed as previously described. The distributions of cell subtypes were visualized within a spatial context over H&E images.

### Chip‐Seq and Data Analysis

All chip‐seq data for NOTCH1 were downloaded in fastq format. Raw reads were cleaned using fastp^[^ ^77^ ^]^ and aligned to the hg38 genome using Bowtie2^[^ ^78^ ^]^ with parameters: ‐t ‐p 15. Genome coverage files for NOTCH1 were generated by deepTools^[^ ^79^ ^]^ with the parameter “bamCoverage –normalizeUsing RPKM”. Peaks were called using MACS2^[^ ^80^ ^]^ and annotated by annotatePeaks.pl in Homer.^[^ ^81^ ^]^ IGV was used to visualize the genomic regions, and the enhancer regions were divided into nonoverlapping windows of different sizes (the windows with reads ≤ 1 / 20 of window size were filtered out).

### Cell–Cell Interaction Analysis

CellChat^[^ ^82^ ^]^ was used with default parameters to identify significant ligand‐receptor pairs between epithelial cells and endothelial cells. All categories of ligand‐receptor interactions in the database for the analysis were used.

### m6A‐Seq and Data Analysis

Total RNA was extracted from the designated biological samples utilizing TRIzol Reagent (ThermoScientific, cat. #15596026) in accordance with the manufacturer's established protocol. Following RNA extraction, genomic DNA was eliminated by treating the samples with DNase I (NEB, cat. #M0303L) to mitigate any potential DNA contamination. The integrity of the isolated RNA was evaluated by determining the A260/A280 ratio using a NanodropTM, OneC spectrophotometer (Thermo Scientific). The integrity of the RNA was assessed utilizing the LabChip GX Touch system (Revvity). The concentration of the qualified RNA was subsequently quantified using a Qubit 3.0 fluorometer in conjunction with the QubitTM RNA Broad Range Assay kit (Thermo Scientific, #Q10210). For the m6A methylated RNA immunoprecipitation (meRIP) assay, the enriched polyA+RNA underwent chemical fragmentation into ≈100‐nucleotide‐long segments through treatment with 20 mm ZnCl2 at 95 °C for 5–10 min. A fraction (10%) of the fragmented RNA was retained as an “Input” control, while the remaining portion was subjected to m6A immunoprecipitation. A specific anti‐N6‐methyladen^[^ ^82^ ^]^osine (m6A) polyclonal antibody (Synaptic Systems, cat. #202003) and RNasin (Promega, cat. #N2615) at a final concentration of 40 U µL^−1^ were introduced to the polyA+RNA, followed by a 2 h incubation at 4 °C with rotation to facilitate the specific binding of the antibody to m6A‐modified RNA. The RNA‐antibody complexes were subsequently immunoprecipitated using Protein‐G magnetic beads (Thermo Scientific, cat. #88848) for 1 h at 4 °C with rotation, ensuring the efficient capture of the m6A‐RNA complexes. Following the initial steps, the Protein‐G magnetic beads underwent thorough washing to eliminate non‐specifically bound RNA, after which the immunoprecipitated RNA was extracted utilizing TRIzol reagent. For the purpose of library preparation, both the input and immunoprecipitated RNA served as substrates for RNA sequencing library construction, employing select components from the KCTM Digital mRNA Library PrepKit (Seqhealth Tech. Co., Ltd., Wuhan, China), in accordance with the manufacturer's guidelines. This kit effectively mitigates duplication bias and errors during PCR and sequencing processes by incorporating unique molecular identifiers (UIDs) composed of 12 random bases to tag the cDNA molecules. The library preparation process encompassed the enrichment of PCR products corresponding to fragment sizes ranging from 200 to 500 base pairs. The enriched libraries were subsequently quantified and sequenced on a Novaseq X Plus platform (Illumina) utilizing the PE150 sequencing mode to generate paired‐end reads. m6A‐seq data were analyzed with an integrated analysis pipeline MeRIPseqPipe^[^ ^83^ ^]^ and visualized them using IGV.

### Public Datasets Used in This Study

To increase the statistical power, the publicly available scRNA‐seq data were downloaded from previous study HRA000087 that are publicly accessible at https://ngdc.cncb.ac.cn/gsa‐human. The available spatial transcriptome data was downloaded from a previous study GSE200310 from the Gene Expression Omnibus database (GEO, https://www.ncbi.nlm.nih.gov/geo/). The public bulk RNA‐seq datasets (GSE102349) were also included. Transcriptomic data and clinical information of the Cancer Genome Atlas (TCGA) cohort were downloaded from the UCSC Xena data portal (https://xenabrowser.net). The Chip‐Seq data for transcription factor NOTCH1 were downloaded from GEO in GSE29600, GSE39263, GSE69156, GSE92873, GSE63010.

### Statistical Analysis

Comparisons between two groups were performed using a two‐tailed Student's *t*‐test under the normality assumption. One‐way ANOVA with Dunnett's T3 multiple‐comparison test was used to compare several groups. Spearman's correlation was used to measure the correlation between two continuous variables, and *r* > 0.3 and *P* < 0.05 was considered significant. Log‐rank test was used for univariate survival analyses and showed as the Kaplan–Meier plot. All statistical analyses and visualization were performed using R or GraphPad Prism. The lines in the middle of the box plot are median, and the upper and lower lines indicate 25th and 75th percentiles. *P* < 0.05 was considered statistically significant. The number of replicates and statistical tests used in the figures is shown in the corresponding figure legends.

### Ethics Declarations—Ethics Approval and Consent to Participate

All samples were collected at Sun Yat‐sen University Cancer Center and were approved by the Ethics Committee of Sun Yat‐sen University Cancer Center (approval number: B2024‐098). All mouse experiments were approved by the Institutional Animal Care and Use Committee of Sun Yat‐sen University Cancer Center (approval number: L102012022003W).

## Conflict of Interest

The authors declare no conflict of interest.

## Author Contributions

C. W., X. L., L. G., J. L., and Y. W. contributed equally to this work. C.W., X.L., X.H., and L.G. conceived and designed the entire project. C.W. and X.L. designed and supervised the research. C.W., L.G. S.C., and S.L. prepared all samples for high‐throughput sequencing. X.L., L.D., and G.H. performed single‐cell RNA‐seq, spatial transcriptome, and m6A‐seq. C.W., Y.C., S.Z., Z.L., W.G., and Y.W. performed experiments in vitro. C.W., J.L., Y.Y., J.H., W.W., and B.Z. performed experiments in vivo. X.L. and L.D. performed statistical and bioinformatics analyses of high‐through sequencing data. Z.Z. supervised all bioinformatics analyses. H.M. and L.T. were responsible for tissue sample preparation. C.W., X.L., S.Z., F.W., X.H., and L.G. prepared the manuscript and all authors commented on the manuscript.

## Code Availability

Any additional information required for the analysis of data in this manuscript is available from the authors upon reasonable request.

## Supporting information



Supporting Information

Supporting Information

Supporting Information

Supporting Information

## Data Availability

scRNA‐seq, spatial transcriptome seq, whole exome sequencing and m6a‐seq raw data have been deposited in the Genome Sequence Archive in BIG Data Center, Beijing Institute of Genomics, Chinese Academy of Sciences under accession number HRA010103 and HRA010084 that are publicly accessible at https://ngdc.cncb.ac.cn/gsa‐human/s/vL8SuLF5 and https://ngdc.cncb.ac.cn/gsa‐human/s/5A2pN31F. Other data is provided within the manuscript or supplementary information files.
