# Supplementary material for: The Tumor‐to‐Endothelial Transfer of FTO Promotes Vascular Remodeling and Metastasis in Nasopharyngeal Carcinoma
Source: Adv Sci (Weinh). 2025 Nov 28;13(8):e09524. doi: 10.1002/advs.202509524 (PMC12884774; doi:10.1002/advs.202509524)
Supplement: Supplementary file 1 — Supporting Information [file ADVS-13-e09524-s002.docx]

Supporting Information

**The Tumor-to-endothelial Transfer of FTO Promotes** **Vascular Remodeling and Metastasis in Nasopharyngeal Carcinoma**

Chun Wu^#^, Xuefei Liu ^#,^ *, Liwen Gu ^#^, Jingru Lian ^#^, Yuting Wang ^#^, Yixin Cheng, Lianhui Duan, Guanyin Huang, Siqi Chen, Boxi Zhao, Sailan Liu, Yufan Yang, Shuqian Zheng, Zijian Lu, Wanping Guo, Jianyang Hu, Wenjing Wang, Zhixiang Zuo^7^, Haiqiang Mai, LinQuan Tang, Songfa Zhang*, Feiqiu Wen*, Xin Hong *, Ling Guo*

This file includes:

Figure S1 to Figure S15


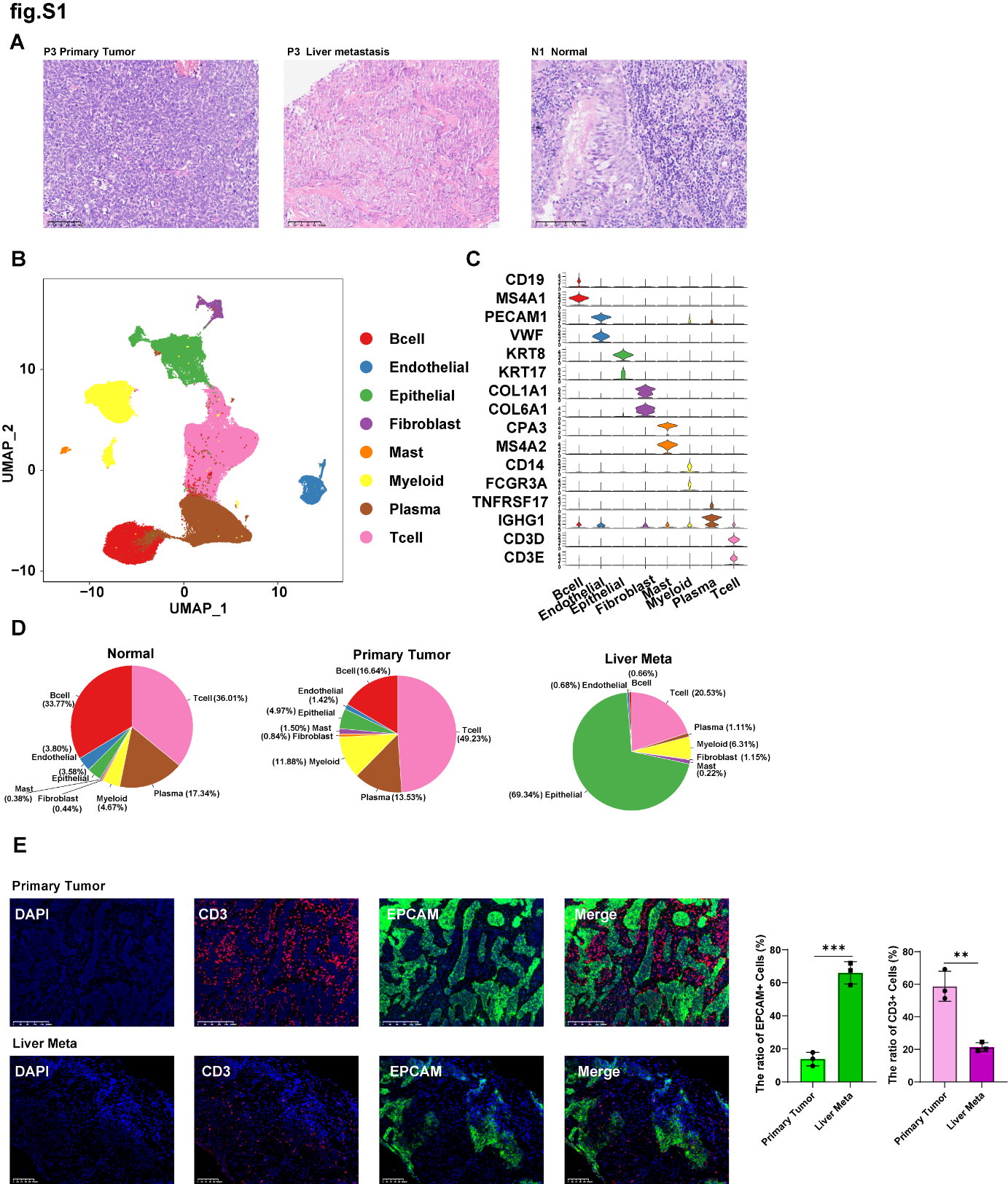


**Figure S1 scRNA-seq profiling of all cells in NPC. Related to Figure 1.**

**(A).** H&E-stained tissue sections showing the morphology of NPC primary tumor, liver metastasis and normal nasopharyngeal tissue. **(B).** UMAP plot showing the subtypes of all cells, each dot indicated a single cell. Color-coded for the cell type. **(C).** Violin plot showing the selected markers for each subtype of all cells. **(D).** Pie charts showing the proportion of the different cell subtypes across normal tissue, primary tumor and liver metastasis.


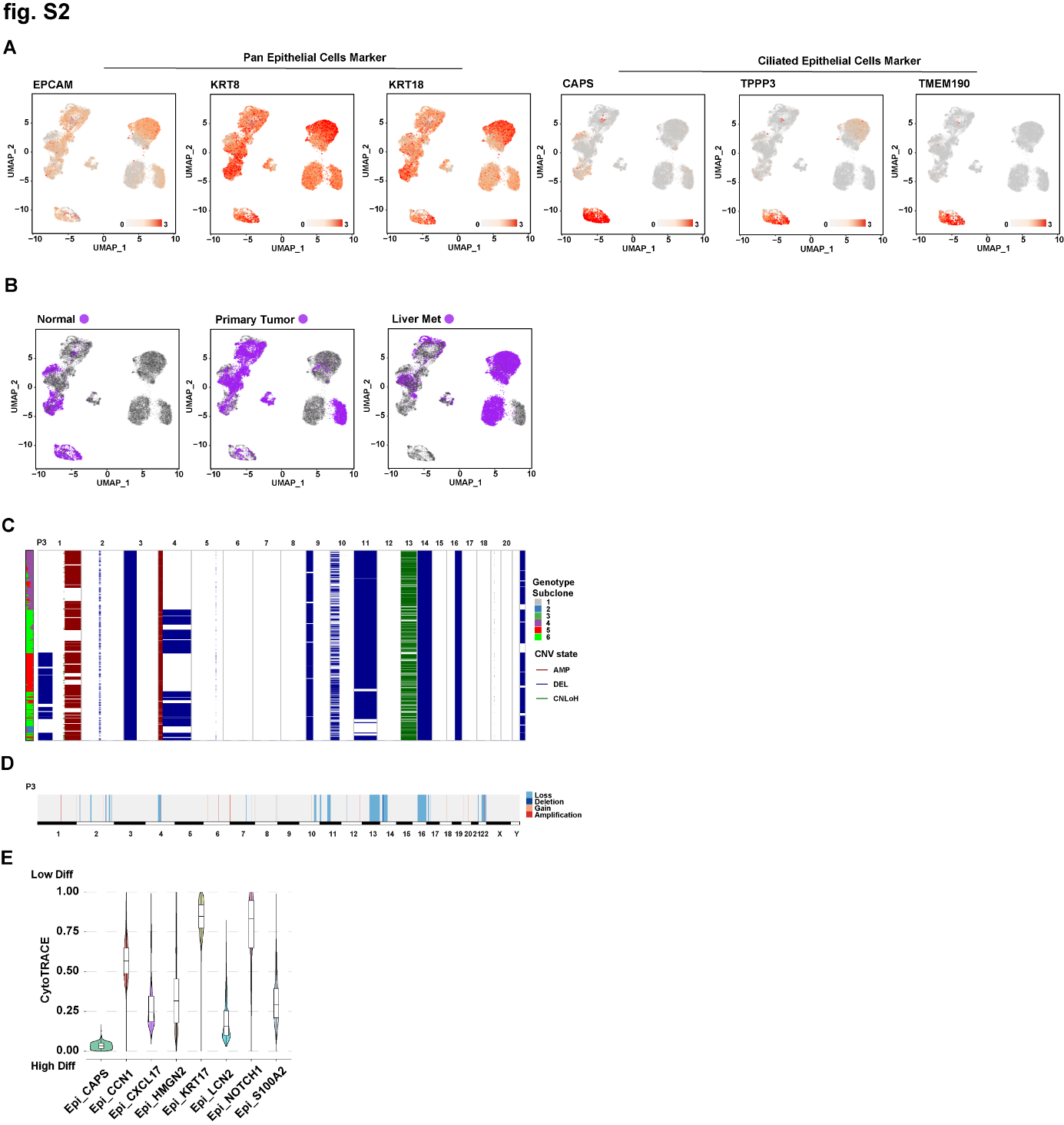


**Figure S2** **Identification of malignant epithelial cells characteristics. Related to Figure 1.**

**(A).** UMAP plot showing the expression levels of *EPCAM, KRT8* and *KRT18*, defined for epithelial cells and *CAPS*, *TPPP3* and *TMEM190*, defined for ciliated cells. **(B).** UMAP plot of epithelial cells showing different tissue origins. **(C).** Heatmap showing large-scale CNVs for epithelial cells from patient P3, inferring from whole exome sequencing. Colors indicate the CNV states. Red: amplifications; blue: deletions. **(D).** Heatmap showing large-scale CNVs for epithelial cells from patient P3, defining from single-cell sequencing. **(E).** Violin plot showing distribution of CytoTRACE scores among 8 epithelial cell subtypes.


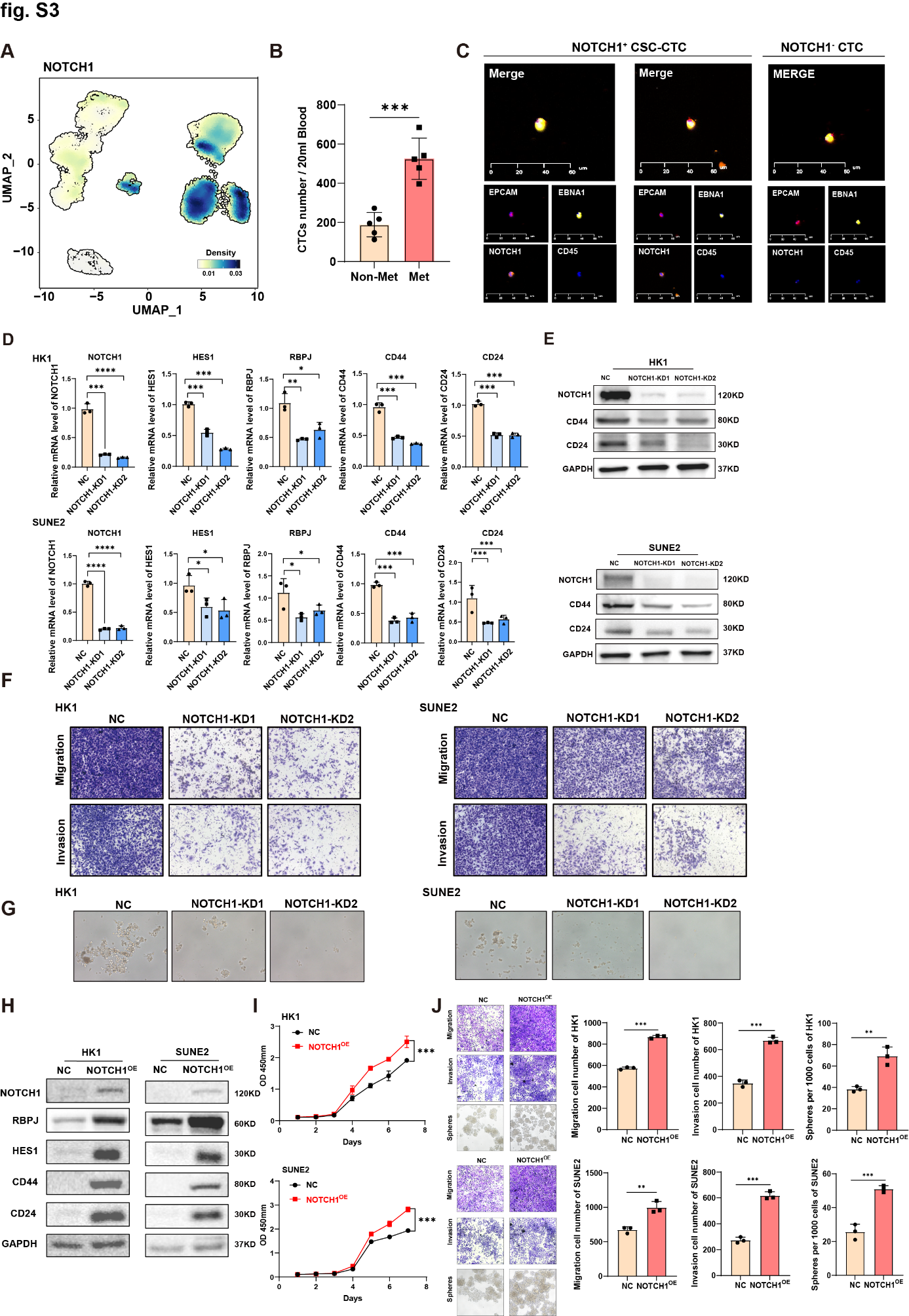


**Figure S3 NOTCH1 enhances the stemness and malignancy of tumor cells. Related to Figure 1.**

**(A).** UMAP plot showing the density distribution of NOTCH1 expression across epithelial subpopulations. **(B).** Bar plot showing the number of circulating tumor cells (CTCs) in non-metastasis and metastasis NPC patients. **(C)**. Immunofluorescence images showing the surface markers (EPCAM, CD45), EBV viral infection marker (EBNA1), and NOTCH1 protein expression in NOTCH1^+^ and NOTCH1^-^ CTCs. Representative images display the co-localization of NOTCH1 (purple), EPCAM (green), CD45 (red), and EBNA1 (yellow), respectively. **(D).** RT-qPCR analysis showing the mRNA expression levels of *NOTCH1*, *HES1*, *RBPJ*, *CD44* and *CD24* in HK1 and SUNE2 cells following NOTCH1 knockdown. **(E).** Western blot analysis displaying the NOTCH1, CD44 and CD24 expressions HK1 and SUNE2 cells following NOTCH1 knockdown. **(F).** Transwell migration and invasion assays assessing the impact of NOTCH1 knockdown on the migration and invasion abilities of HK1 and SUNE2 cells. **(G).** The sphere formation assays showed the reduced sphere-propagating capacity of HK1 and SUNE2 cells following NOTCH1 knockdown. **(H).** Western blot analysis displaying the NOTCH1, RBPJ, HES1, CD44 and CD24 expression in HK1 and SUNE2cells following NOTCH1 overexpression. **(I).** CCK-8 assay showing the effect of NOTCH1 overexpression on the proliferation of HK1 and SUNE2 cells. **(J).** Transwell migration, invasion and sphere formation assays assessing the impact of NOTCH1 overexpression on the migration, invasion sphere-propagating abilities of HK1 and SUNE2 cells. Data in (**D, I and J**) are mean ± SD from 3 independent experiments, **P* < 0.05, ***P* < 0.01 and ****P* < 0.001 were determined by two-way ANOVA with Bonferroni’s post test.


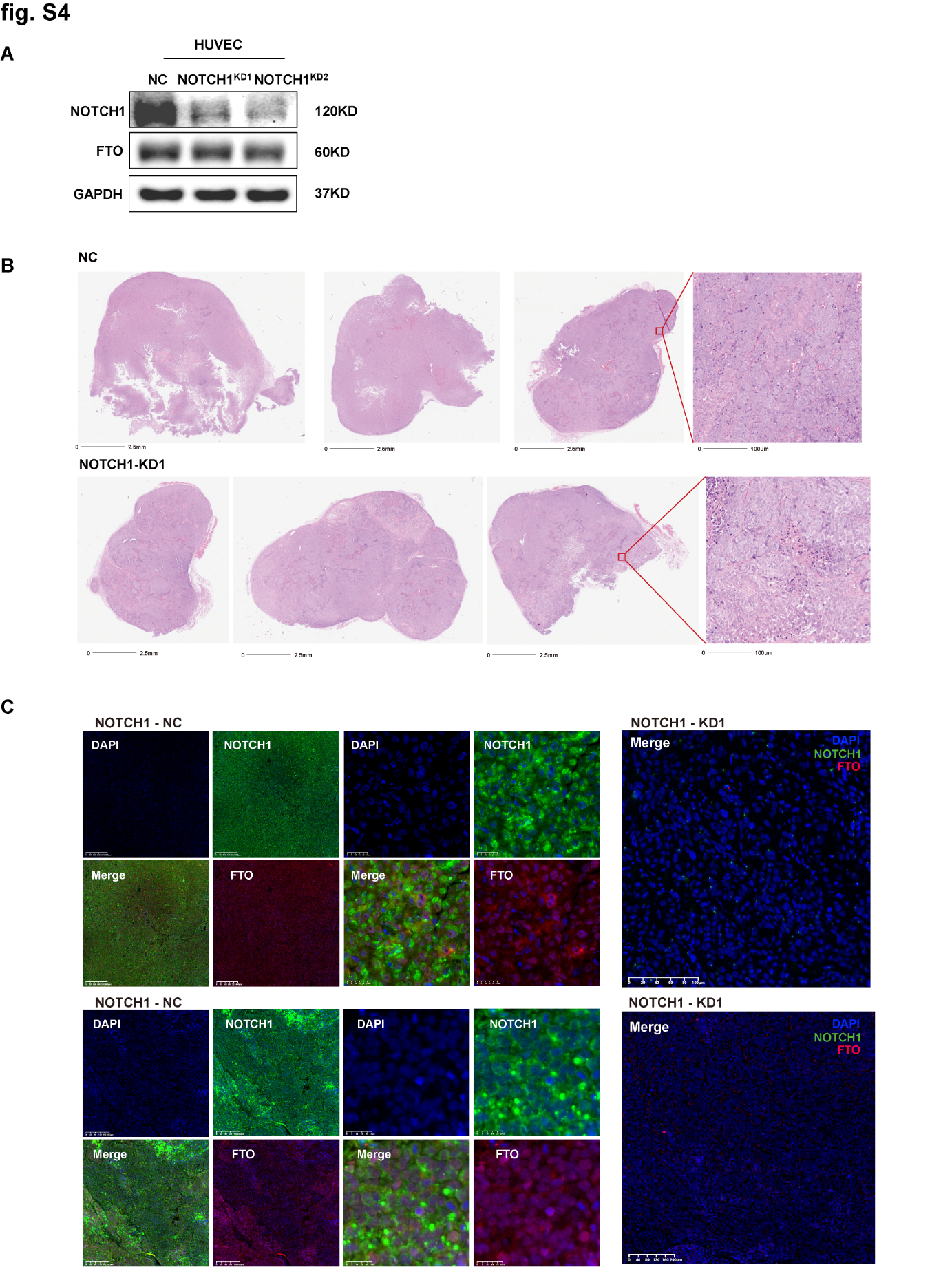


**Figure S4 Histological analysis of xenograft tumors after NOTCH1 knockdown *in vivo*. Related to Figure 2.**

**(A).** Western blot analysis displaying the NOTCH1 and FTO expressions in HUVEC cells following NOTCH1 knockdown

**(B).** The representative H&E images showing central region of the tumor in xenograft tumors in vivo. **(C).** mIHC images showing the expression of NOTCH1 (green) and FTO (red) in xenograft tumors. In the NOTCH1 knockdown group, FTO expression is substantially diminished, as visualized by DAPI staining and merge panels.


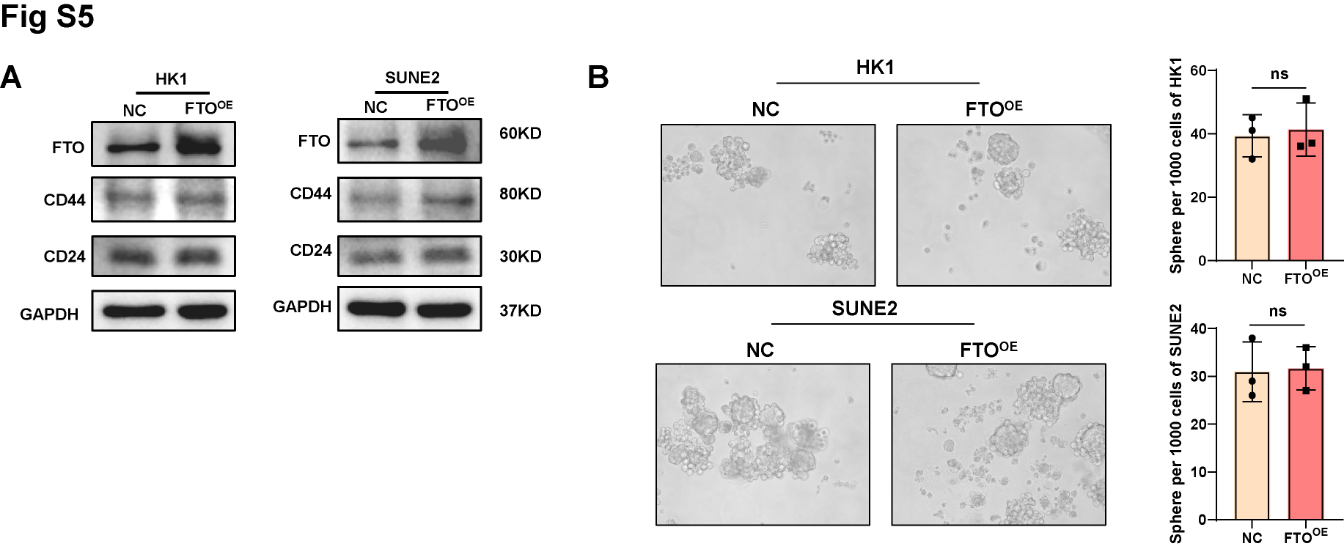


**Figure S5 FTO does not appear to be involved in CSC maintenance. Related to Figure 2.**

**(A).** Western blot analysis displaying the FTO, CD44 and CD24 expressions HK1 and SUNE2 cells following FTO overexpression.

**(B).** Sphere formation assays assessing the impact of FTO overexpression on the sphere-propagating abilities of HK1 and SUNE2 cells.


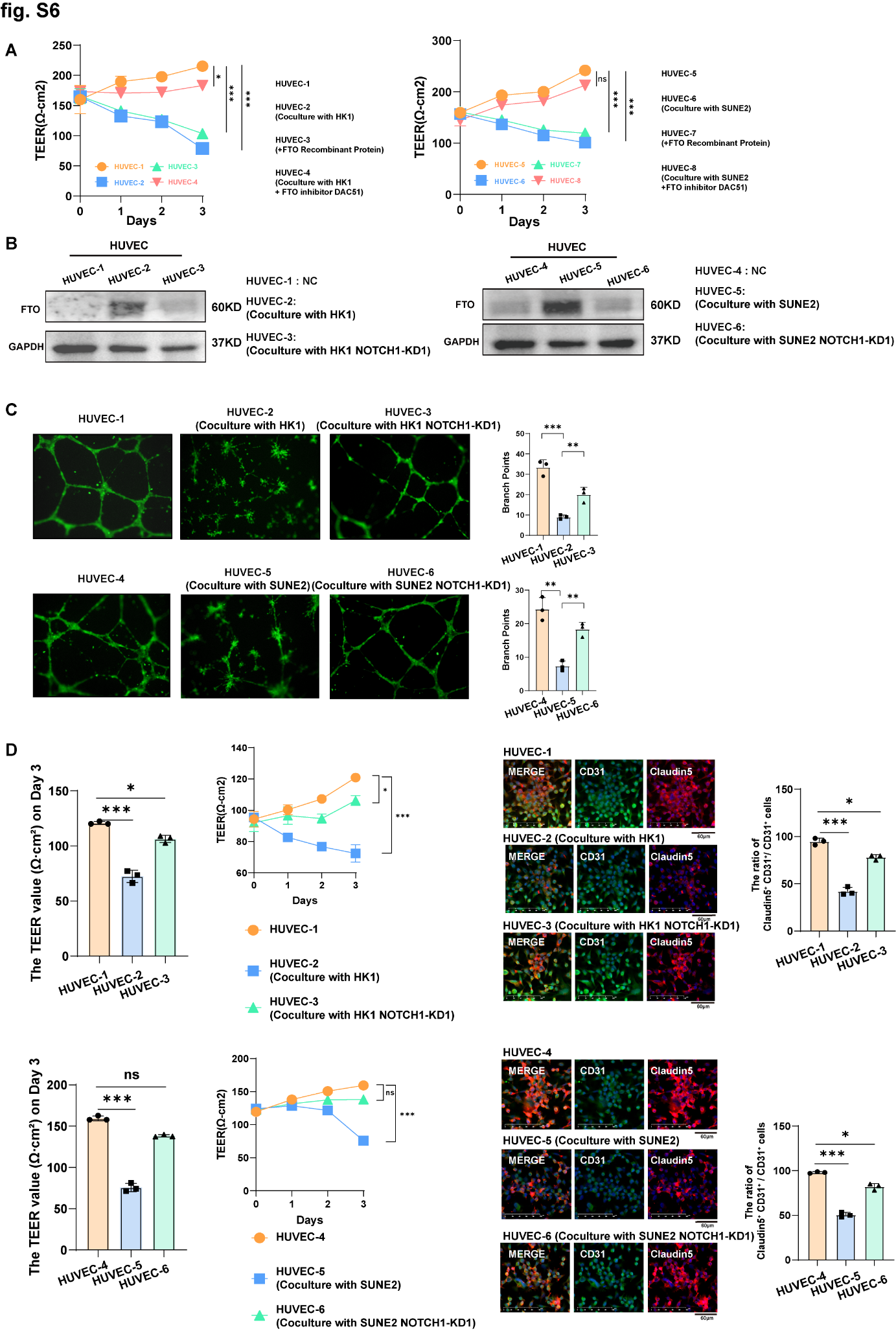


**Figure S6 Depletion of NOTCH1 at least partially rescues endothelial dysfunction in the co-culture model of endothelial cells with tumor cells. Related to Figure 3.**

**(A).** The values of trans-endothelial electrical resistance (TEER) showing permeability of HUVECs under cultured alone, co-cultured with malignant cells, added FTO recombinant protein and co-cultured with malignant cells treated with FTO inhibitor DAC51. **(B).** Western blot analysis showing the FTO expression in HUVEC cultured alone (HUVEC1, HUVEC4), HUVEC cocultured with HK1 or SUNE2 (HUVEC2, HUEVC5) and HUVEC cocultured with NOTCH1-KD HK1 or SUNE2 (HUVEC3, HUEVC6). **(C).** The tube formation assay showing tube-forming ability of HUVECs under HUVEC cultured alone (HUVEC1, HUVEC4), HUVEC cocultured with HK1 or SUNE2 (HUVEC2, HUEVC5) and HUVEC cocultured with NOTCH1-KD HK1 or SUNE2 (HUVEC3, HUEVC6). **(D).** TEER showing permeability of HUVECs under various experimental groupings. The groupings are identical to that in **(B)** (left panel). Bar plot showing comparison of the proportion of Claudin-5^+^CD31^+^ /CD31^+^ cells in different groups (right panel).


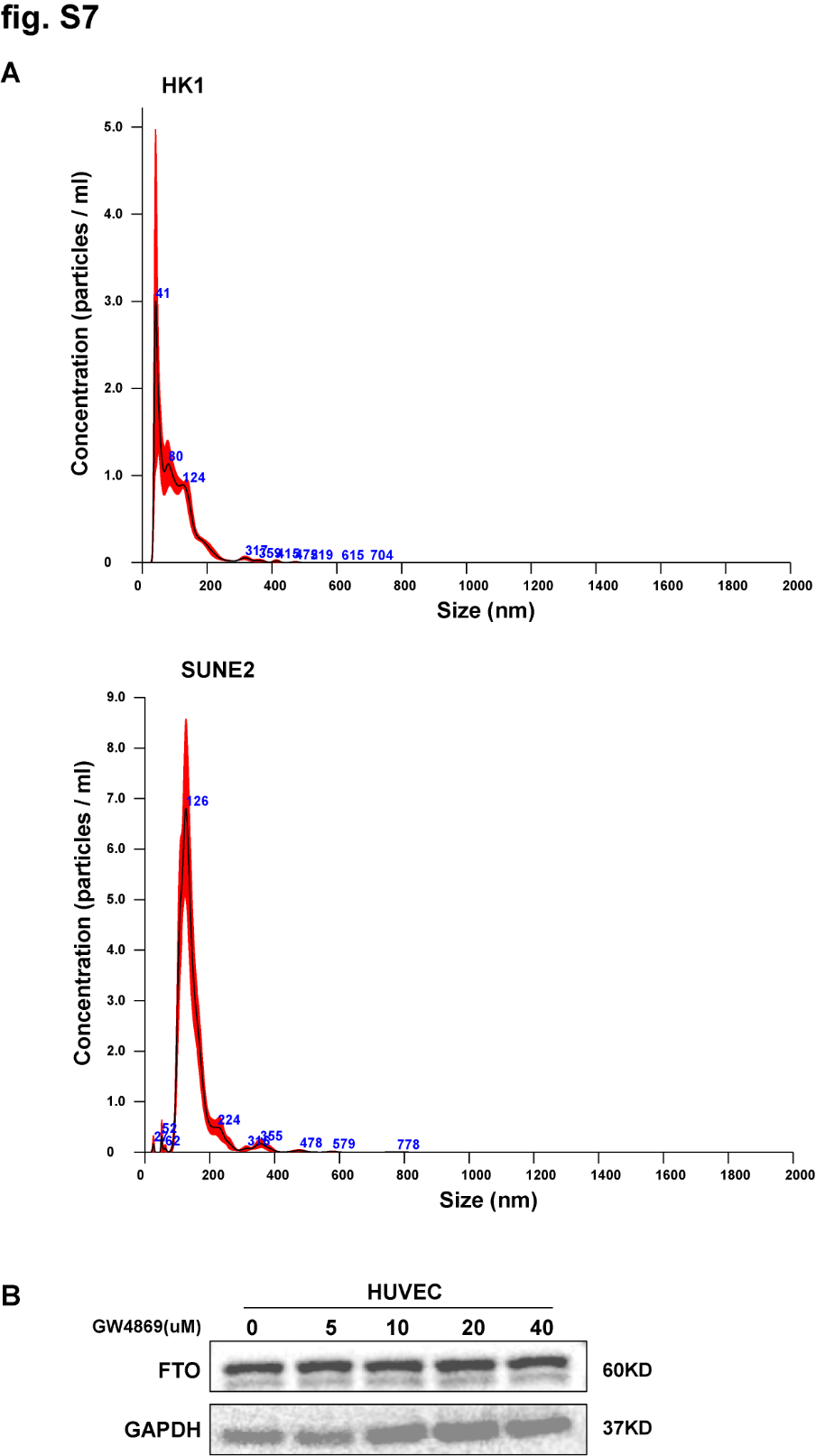


**Figure S7 Tumor cell-derived exosomes contain FTO proteins. Related to Figure 3.**

**(A).** Nano-tracking analysis (NTA) showing the particle diameter range of EVs. **(B).** Western blot analysis showing unaffected FTO protein levels in HUVEC cells after added the exosome inhibitor GW4869 by concentration gradient.


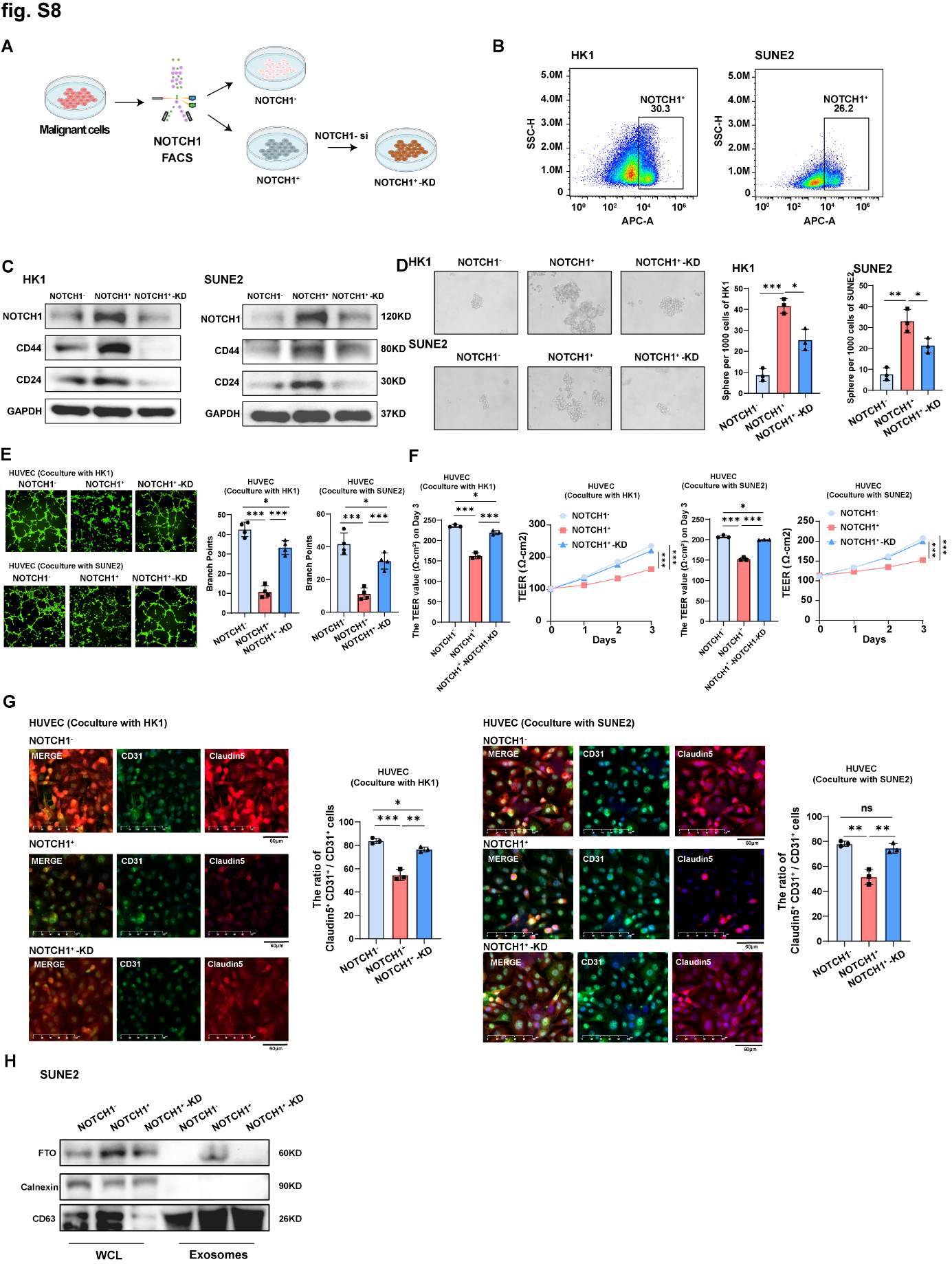


**Figure S8 NOTCH1+ CSC impacts endothelial integrity through FTO protein secretion. Related to Figure 3.**

**(A).** Schematic of cell sorting: NOTCH1+ and NOTCH1− subpopulations were isolated from HK1 and SUNE2 cell lines by FACS. The NOTCH1+ subpopulation was further subjected to the NOTCH1 knockdown, yielding three experimental groups. **(B).** FACS plots showing NOTCH1+ and NOTCH1− subpopulations were isolated from HK1 and SUNE2 cell lines. **(C).** Western blot showing the stemness-related genes such as NOTCH1, CD44 and CD24 in NOTCH1+ subpopulation and NOTCH1− subpopulation, NOTCH1+ with NOTCH1 knockdown subpopulation in HK1 cells or SUNE2 cells. **(D).** Sphere-propagating capacity in NOTCH1+ subpopulation and NOTCH1− subpopulation, NOTCH1+ with NOTCH1 knockdown subpopulation in HK1 cells or SUNE2 cells. (E). The tube formation assay showing tube-forming ability of HUVECs co-cultured with NOTCH1+ subpopulation and NOTCH1− subpopulation, NOTCH1+ with NOTCH1 knockdown subpopulation. **(F).** The values of trans-endothelial electrical resistance (TEER) showing permeability of HUVECs co-cultured with NOTCH1+ subpopulation and NOTCH1− subpopulation, NOTCH1+ with NOTCH1 knockdown subpopulation. **(G).** mIHC showing the tight junction markers Claudin-5 expression in endothelial cells and bar plot showing comparison of the proportion of Claudin-5+CD31+ /CD31+ cells in different groups. **(H).** Western blot showing the exosome positive (CD63) and negative markers (Calnexin) confirming exosome isolation from the co-culture system and the protein expression level of FTO in exosomes under HUVECs co-cultured with NOTCH1+ subpopulation and NOTCH1− subpopulation, NOTCH1+ with NOTCH1 knockdown subpopulation.


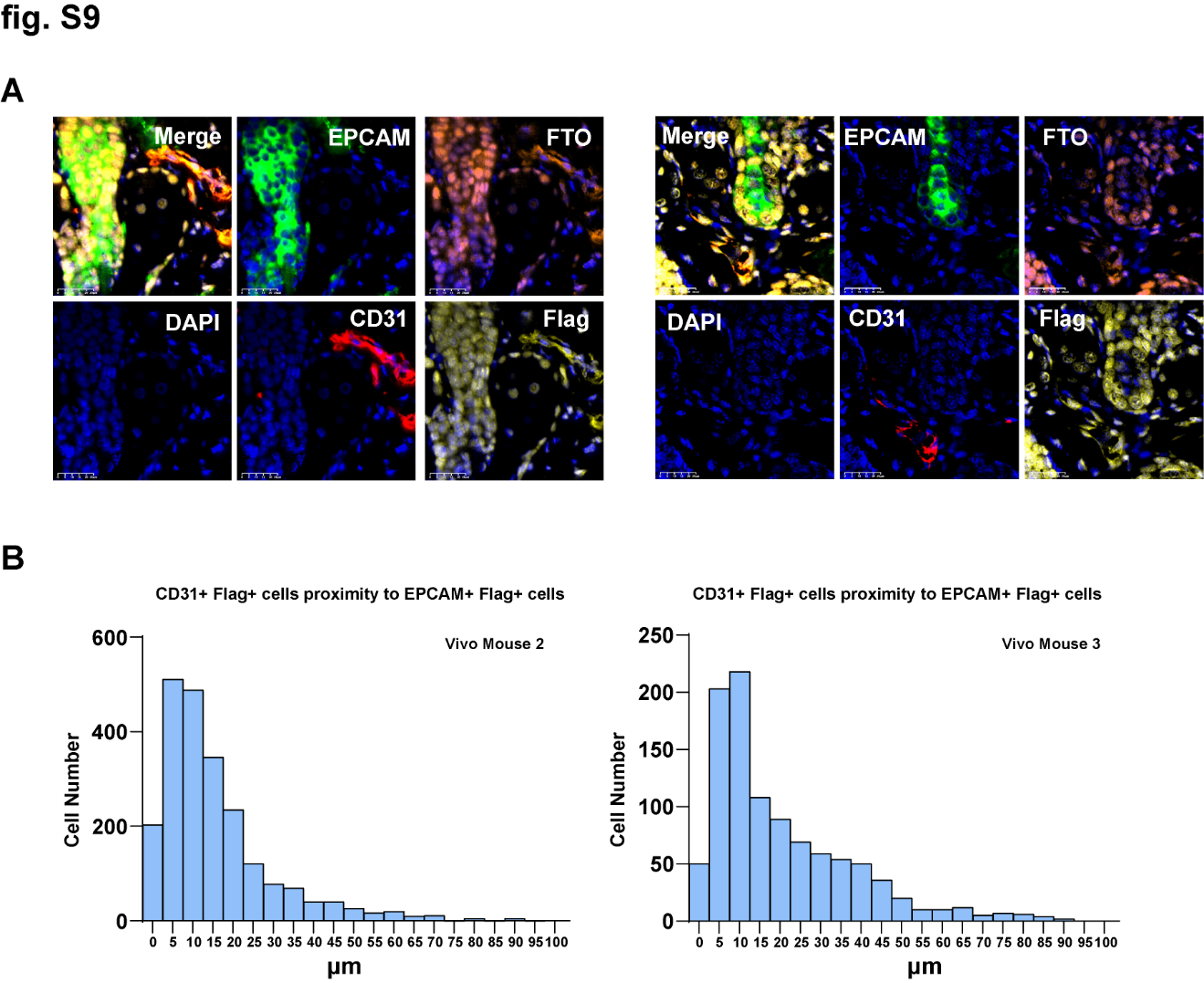


**Figure S9 mIHC analysis and cell proximity assessment in subcutaneous co-injection model. Related to Figure 3.**

**(A).** mIHC showing staining of tumor tissue sections from subcutaneous tumor models with DAPI (blue) for nuclei, CD31 (red) for endothelial cells, EPCAM (green) for epithelial cells, FTO (orange) for target protein, and Flag (yellow) for target protein. The merged image illustrates the localization of these markers.

**(B).** Bar chart showing the proximity of CD31^+^ Flag^+^ cells to EPCAM^+^ Flag^+^ cells in tumor tissue from vivo Mouse2-3.


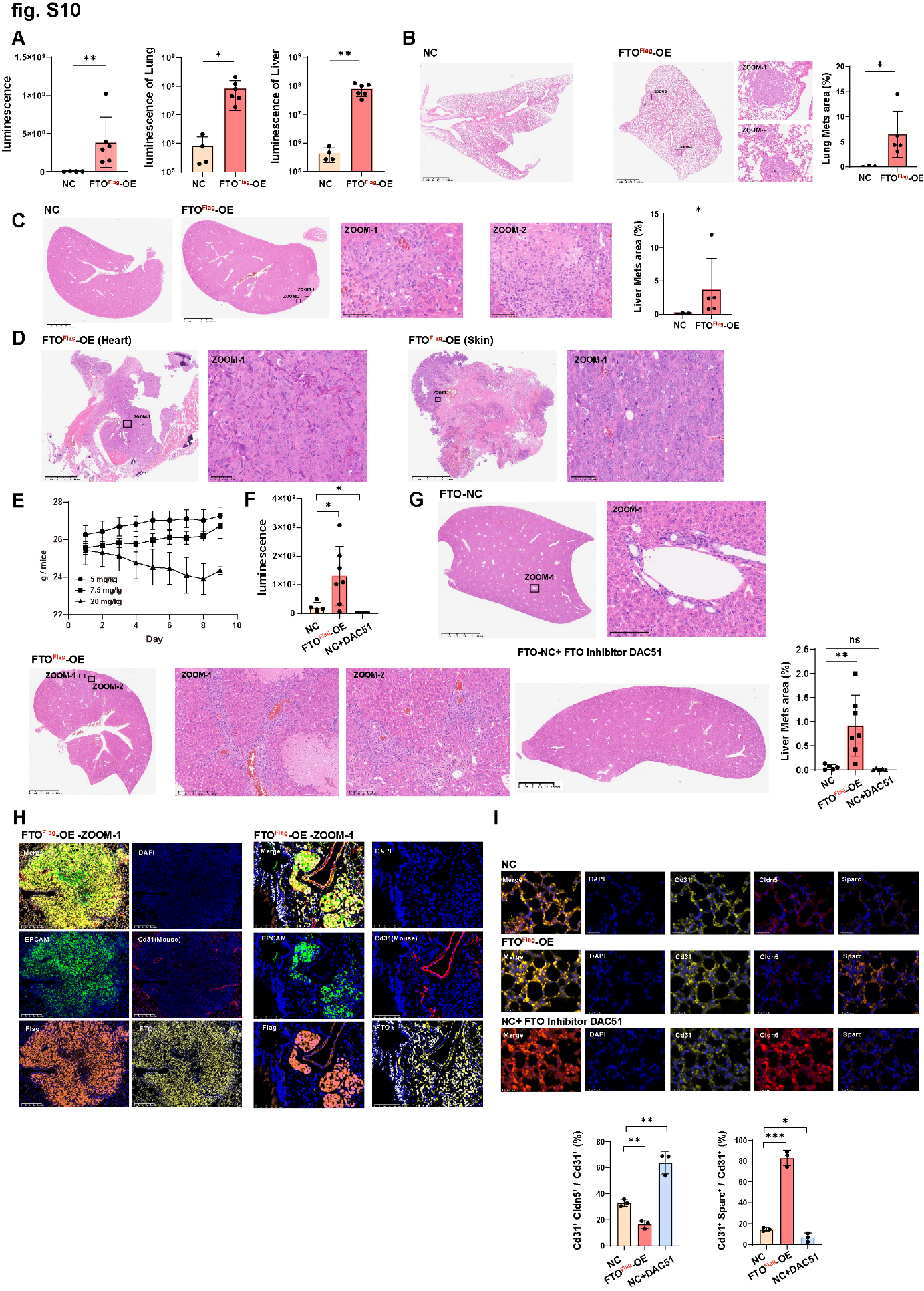


**Figure S10 Metastatic potential of control or FTO-OE HK1 cells by tail vein injection and the anti-tumor effects of FTO inhibitor DAC51 *in vivo*. Related to Figure 4**

**(A).** Bar plot shows the fluorescence values of whole body, lung and liver signal obtained from IVIS imaging captured at the experimental endpoint for the two experimental groups (NC and Flag-FTO-OE groups). **(B).** H&E staining of lungs dissected from two experimental groups (NC and Flag-FTO-OE) of the metastatic mouse model. **(C).** H&E staining of livers dissected from two experimental groups (NC and Flag-FTO-OE) of the metastatic mouse model. **(D)**. H&E staining of heart and skin dissected from Flag-FTO-OE group of the metastatic mouse model. **(E)**. Line plot showing drug toxicity of DAC51 in three conditions under continuous observation for nine days. **(F).** Bar plot shows the fluorescence values obtained from IVIS imaging captured at the experimental endpoint for the three experimental groups (NC, Flag-FTO-OE and NC-DAC51 treatment groups). **(G).** H&E staining of livers dissected from three experimental groups (NC, Flag-FTO-OE and NC-DAC51 treatment groups) of the metastatic mouse model. **(H).** mIHC images showing the expression of EPCAM (green), FTO (yellow), Cd31^+^ (red) and Flag^+^ (orange) in xenograft tumors in different groups. **(I).** mIHC showed the expression of Cldn5 (Red), Sparc (Orange), Cd31 (Yellow) and DAPI (Blue) from three experimental groups (NC, Flag-FTO-OE and NC treated with DAC51) of the lungs metastatic mouse model. Boxplot showing the fraction of Cldn5^+^ Cd31^+^ cells and Sparc^+^ Cd31+ cells among all Cd31^+^ cells in three experimental groups (right). ***p* < 0.01, ***p* < 0.01 and *** *p* < 0.001, two-tailed Student's t-test.


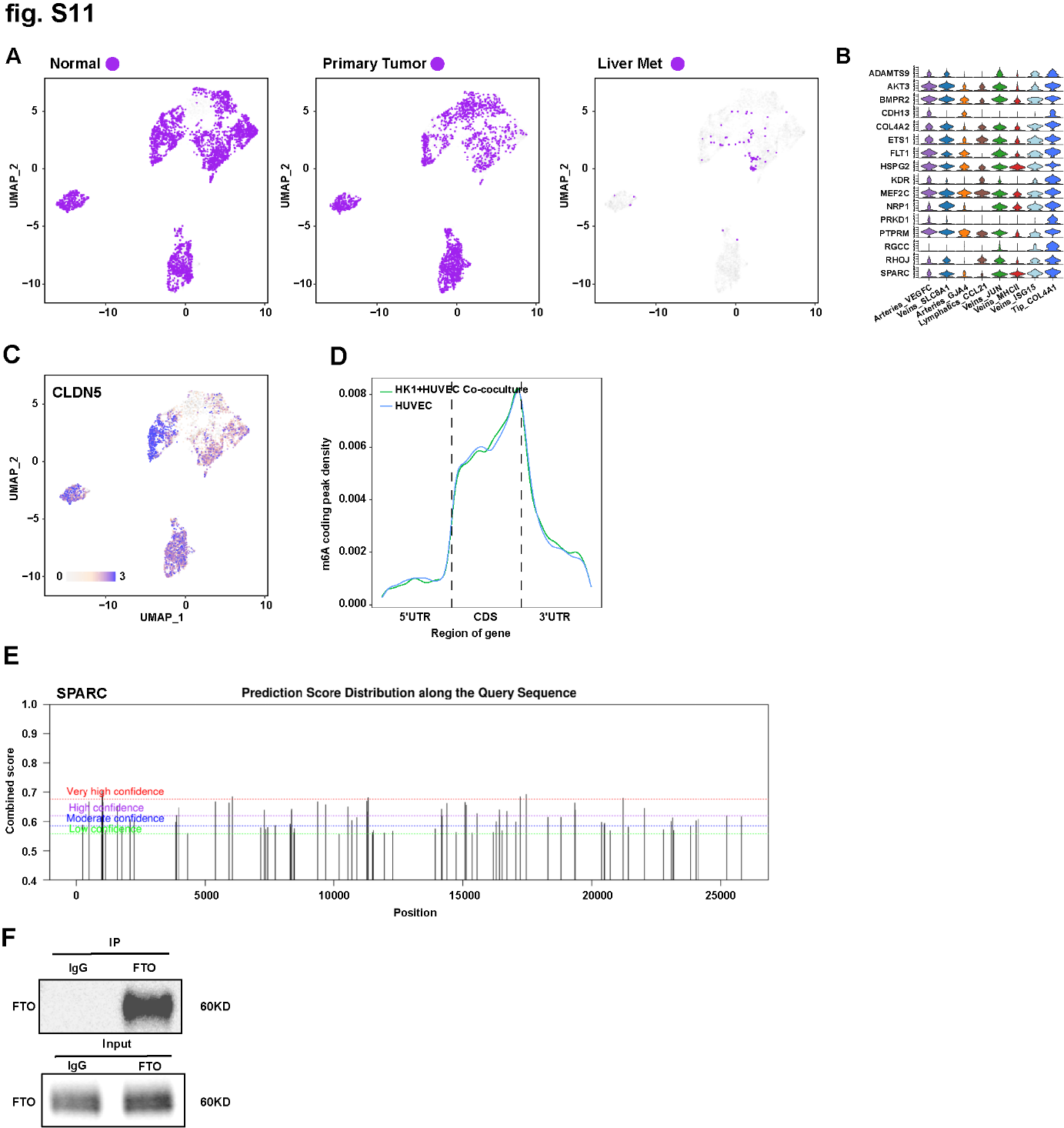


**Figure S11 Landscape of endothelial cells in NPC. Related to Figure 5.**

**(A).** UMAP plot showing endothelial cells derived from different tissue sources. **(B).** Violin plot showing the selected markers for each subtype of endothelial cells. **(C).** UMAP plot showing the expression levels of CLDN5 in endothelial cells. **(D).** Density curve showing the distribution of m6A peaks across the transcripts. The transcript is divided into three parts, namely 5’ UTR, CDS, and 3’UTR. **(E).** The prediction of m6A RNA modification sites in SPARC. **(F).** Western blotting was performed with FTO antibodies to show immunoprecipitation efficiency.


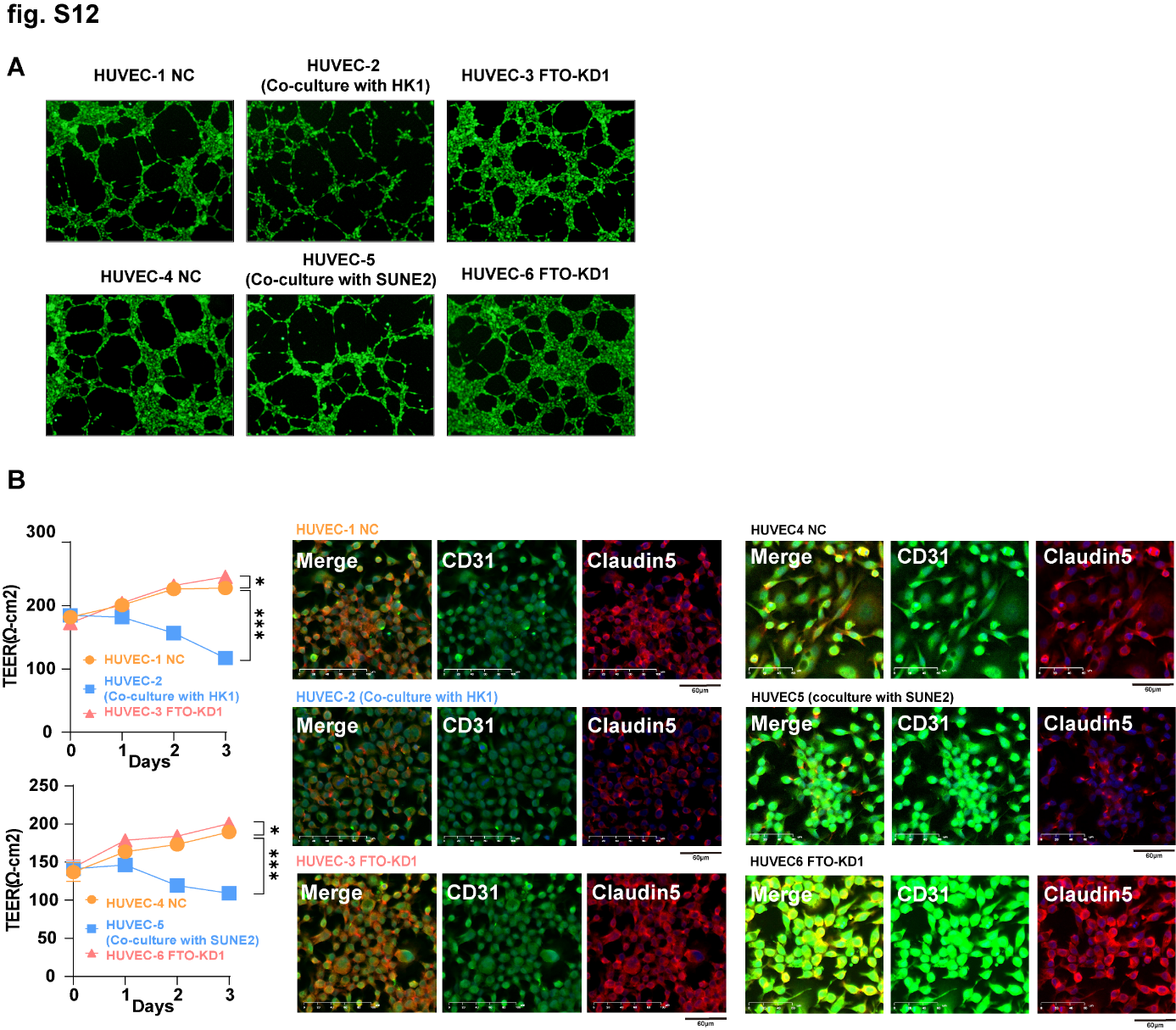


**Figure S12 The tube formation and TEER assay in HUVEC in different culture conditions. Related to Figure 5**

**(A).** The tube formation assays showing tube-forming ability of HUVECs under HUVEC cultured alone (HUVEC1, HUEVC4), HUVEC cocultured with HK1 or SUNE2 (HUVEC2, HUEVC5) and HUVEC with FTO knockdown cultured alone (HUVEC3, HUEVC6). **(B).** TEER showing permeability of HUVECs under various experimental groupings. The groupings are identical to that in **(A).** The Immunofluorescence showing the expression levels of CD31 and Cladin5 in HUVECs under various experimental groupings. The groupings are identical to that in **(A).**


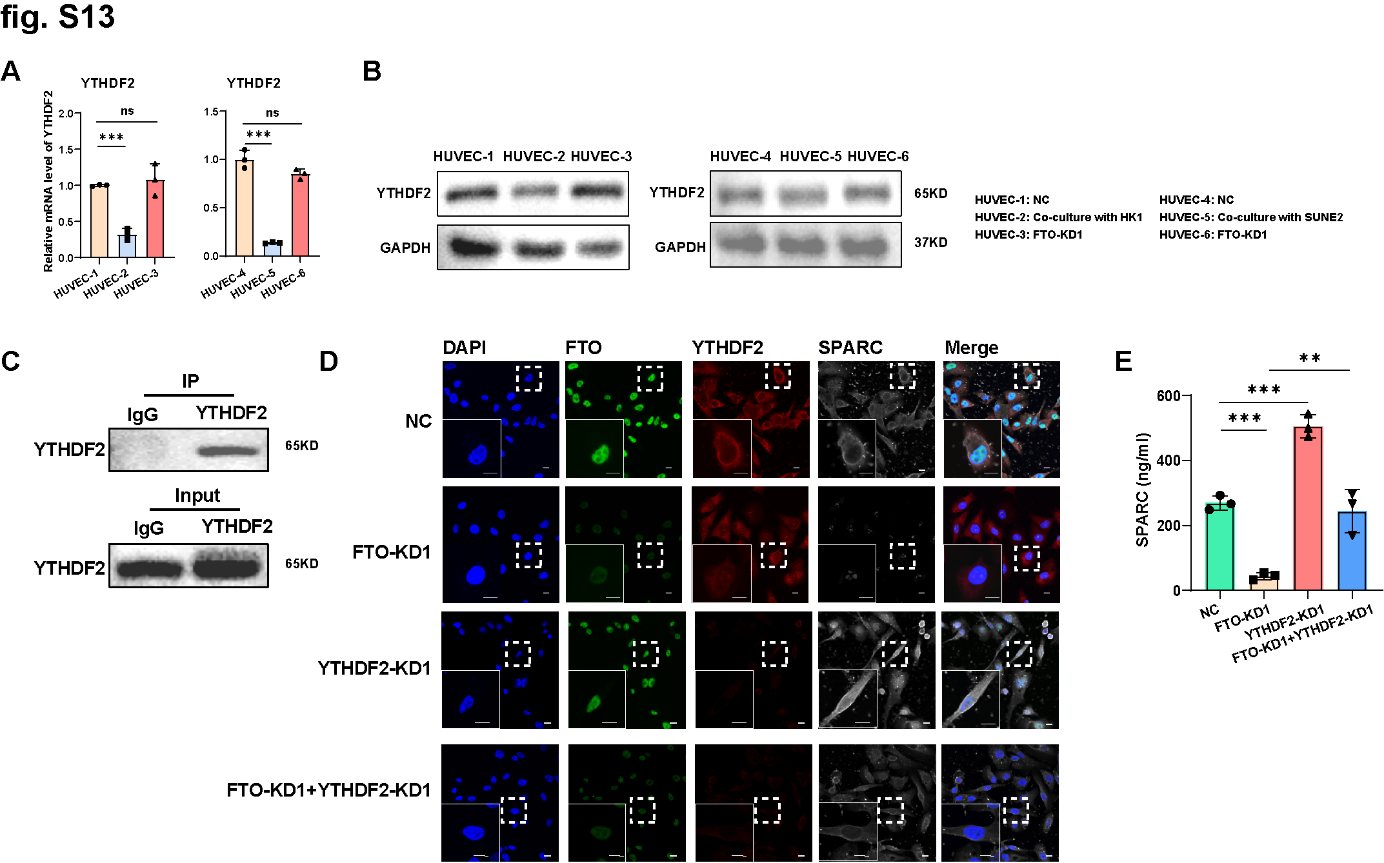


**Figure S13 Changes in YTHDF2 expression levels in different culture conditions. Related to Figure 5.**

**(A).** RT-qPCR showing the mRNA expression levels of *YTHDF2* in HUVEC under HUVEC cultured alone (HUVEC1, HUEVC4), HUVEC cocultured with HK1 or SUNE2 (HUVEC2, HUEVC5) and HUVEC with FTO knockdown cultured alone (HUVEC3, HUEVC6). **(B).** Western blot showing the protein expression levels of YTHDF2 in HUVECs under various experimental groupings. The groupings are identical to that in **(A)**. **(C).** Western blotting was performed with YTHDF2 antibodies to show immunoprecipitation efficiency. **(D)**. Fluorescent photographs of HUVEC cells in control group, FTO-KD group, YTHDF2-KD group and FTO/YTHDF2 double knockdown group. DAPI (blue), FTO (green), YTHDF2 (red) and SPARC (gray) in HUVEC cells, each stain was shown separately and merged. Scale bars: 10 µm. **(E)**. Bar plot showing SPARC protein levels in culture medium by ELISA in control group, FTO-KD group, YTHDF2-KD group and FTO/YTHDF2 double knockdown group.


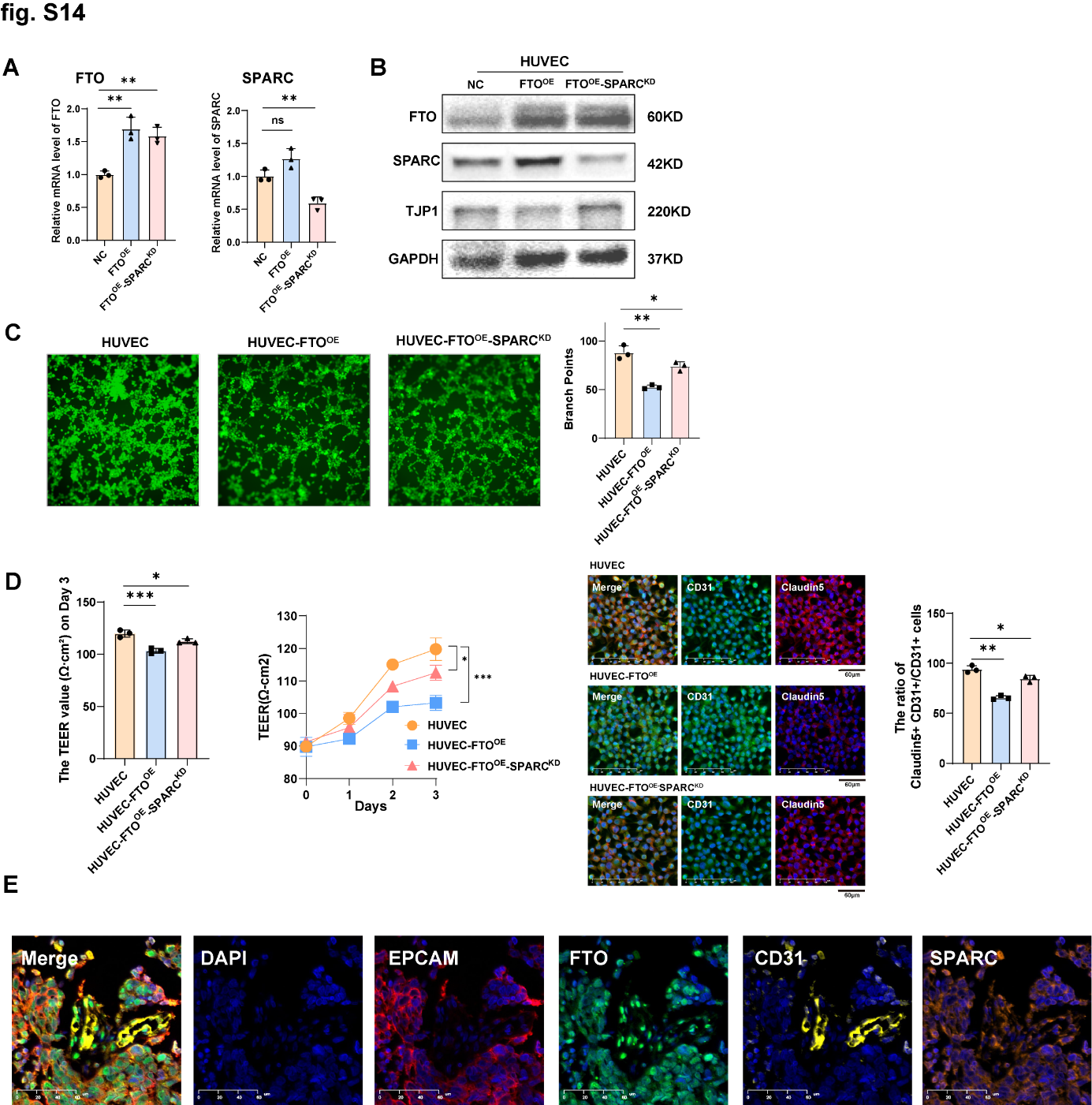


**Figure S14 SPARC protein disrupts the endothelial integrity. Related to Figure 6.**

**(A).** RT-qPCR showing the mRNA expression levels of *FTO* and *SPARC* under FTO overexpression, combined FTO overexpression and SPARC knockdown conditions. **(B).** Western blot showing the effect of FTO overexpression, combined FTO overexpression and SPARC knockdown conditions on the protein expression level of FTO, SPARC and TJP1. **(C).** The tube formation assay showing tube-forming ability of HUVECs under FTO overexpression, combining FTO overexpression and SPARC knockdown conditions. **(D).** TEER showing permeability of HUVECs under FTO overexpression, combined FTO overexpression and SPARC knockdown conditions (left panel). The Immunofluorescence showing the tight junction markers Claudin-5 expression in endothelial cells (middle panel) and bar plot showing comparison of the proportion of Claudin-5^+^ CD31^+^ /CD31^+^ cells in different groups (right panel). **(E).** mIHC showing the expression of FTO (green), EPCAM (red), CD31 (yellow), and SPARC (orange) in NPC tissues.


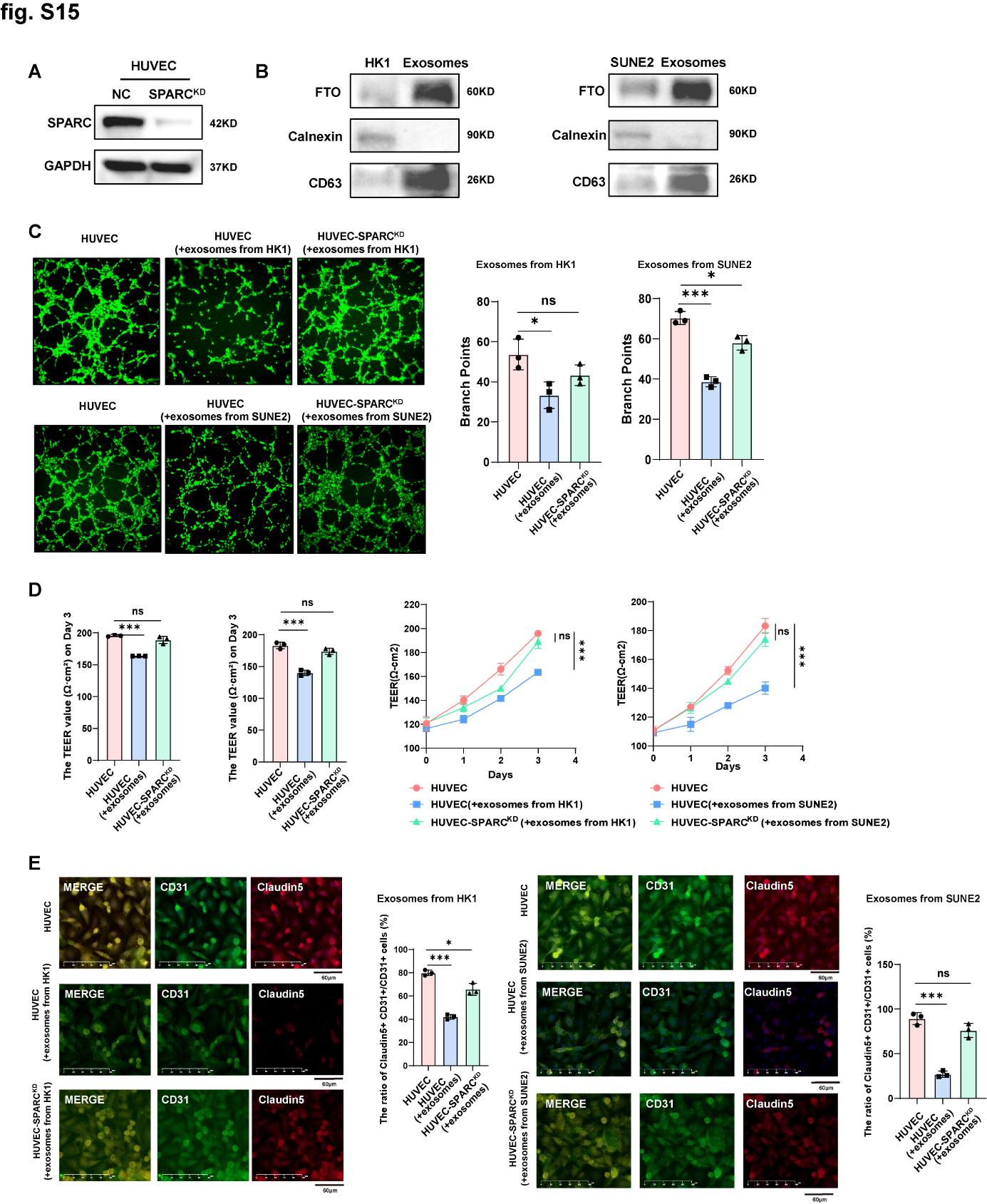


**Figure S15 Tumor-derived exosomal FTO mediates endothelial dysfunction via SPARC Related to Figure 6.**

**(A).** Western blot showing SPARC expression in HUVEC cells and SPARC knockdown (SPARC-KD) HUVAC cells. **(B).** Western blot showing the exosome positive (CD63) and negative markers (Calnexin) confirming exosome isolation from HK1 and SUNE2 cells and the protein expression level of FTO in exosomes from HK1 and SUNE2 cells. **(C).** The tube formation assay showing tube-forming ability of HUVECs in control HUVEC, HUVAC exposed to tumor-derived exosomes, SPARC-KD HUVAC exposed to tumor-derived exosomes. **(D).** The values of trans-endothelial electrical resistance (TEER) showing permeability of HUVECs in different groups. The groupings are identical to that in **(C).** **(E).** mIHC showing the tight junction markers Claudin-5 expression in endothelial cells and bar plot showing comparison of the proportion of Claudin-5+CD31+ /CD31+ cells in different groups. The groupings are identical to that in **(C).**
