# Supplementary material for: The Tumor‐to‐Endothelial Transfer of FTO Promotes Vascular Remodeling and Metastasis in Nasopharyngeal Carcinoma
Source: Adv Sci (Weinh). 2025 Nov 28;13(8):e09524. doi: 10.1002/advs.202509524 (PMC12884774; doi:10.1002/advs.202509524)
Supplement: Supplementary file 3 — Supporting Information [file ADVS-13-e09524-s001.pdf]

Figure3 E

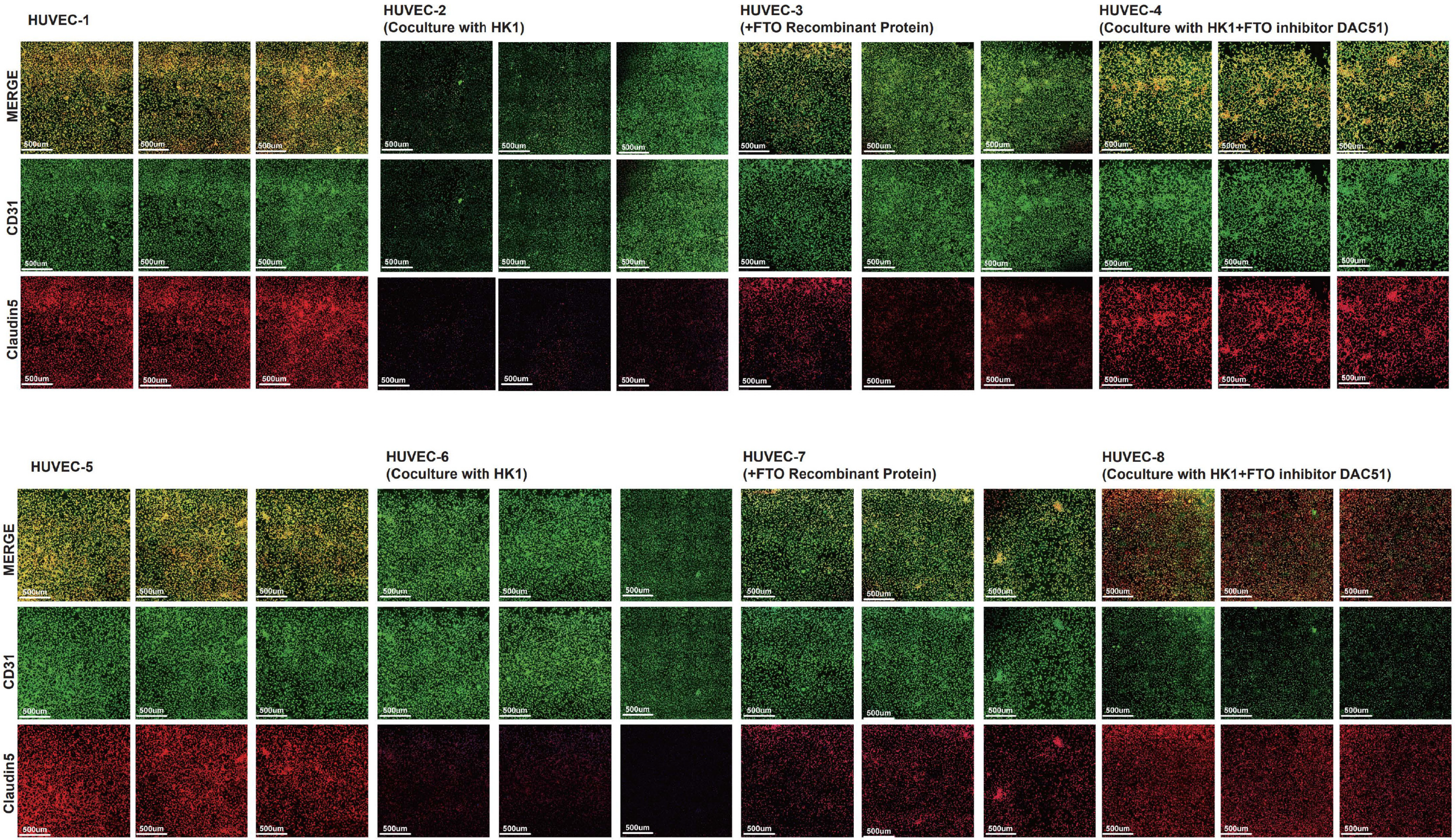
HUVEC-1
HUVEC-2
(Coculture with HK1)
HUVEC-3
(+FTO Recombinant Protein)
HUVEC-4
(Coculture with HK1+FTO inhibitor DAC51)
MERGE
CD31
Claudin5
500um
HUVEC-5
HUVEC-6
(Coculture with HK1)
HUVEC-7
(+FTO Recombinant Protein)
HUVEC-8
(Coculture with HK1+FTO inhibitor DAC51)

Figure6 D

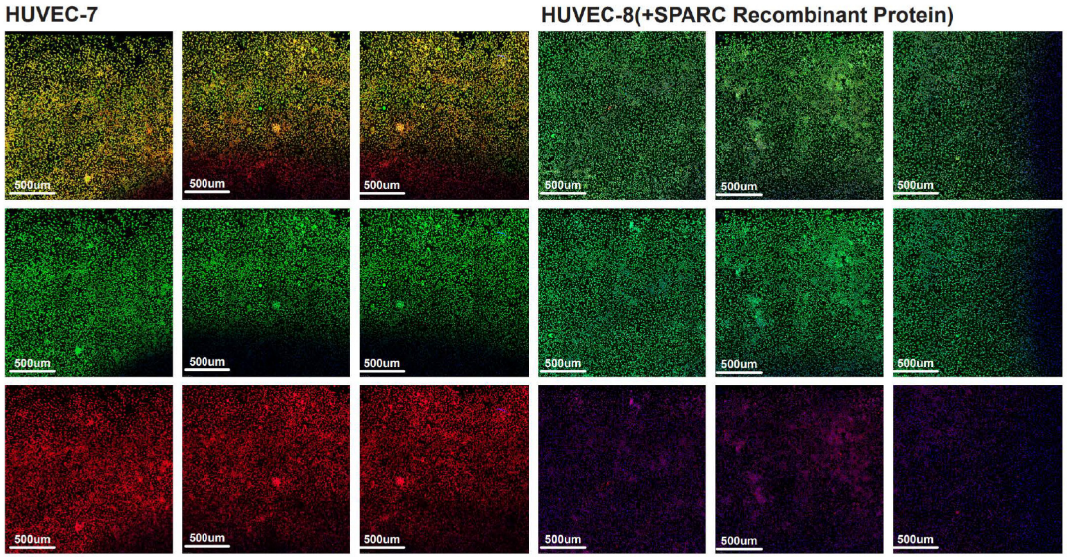
HUVEC-7
HUVEC-8(+SPARC Recombinant Protein)
500um
500um
500um
500um
500um
500um
500um
500um
500um
500um
500um
500um
500um
500um
500um
500um
500um
500um

FigureS6 D

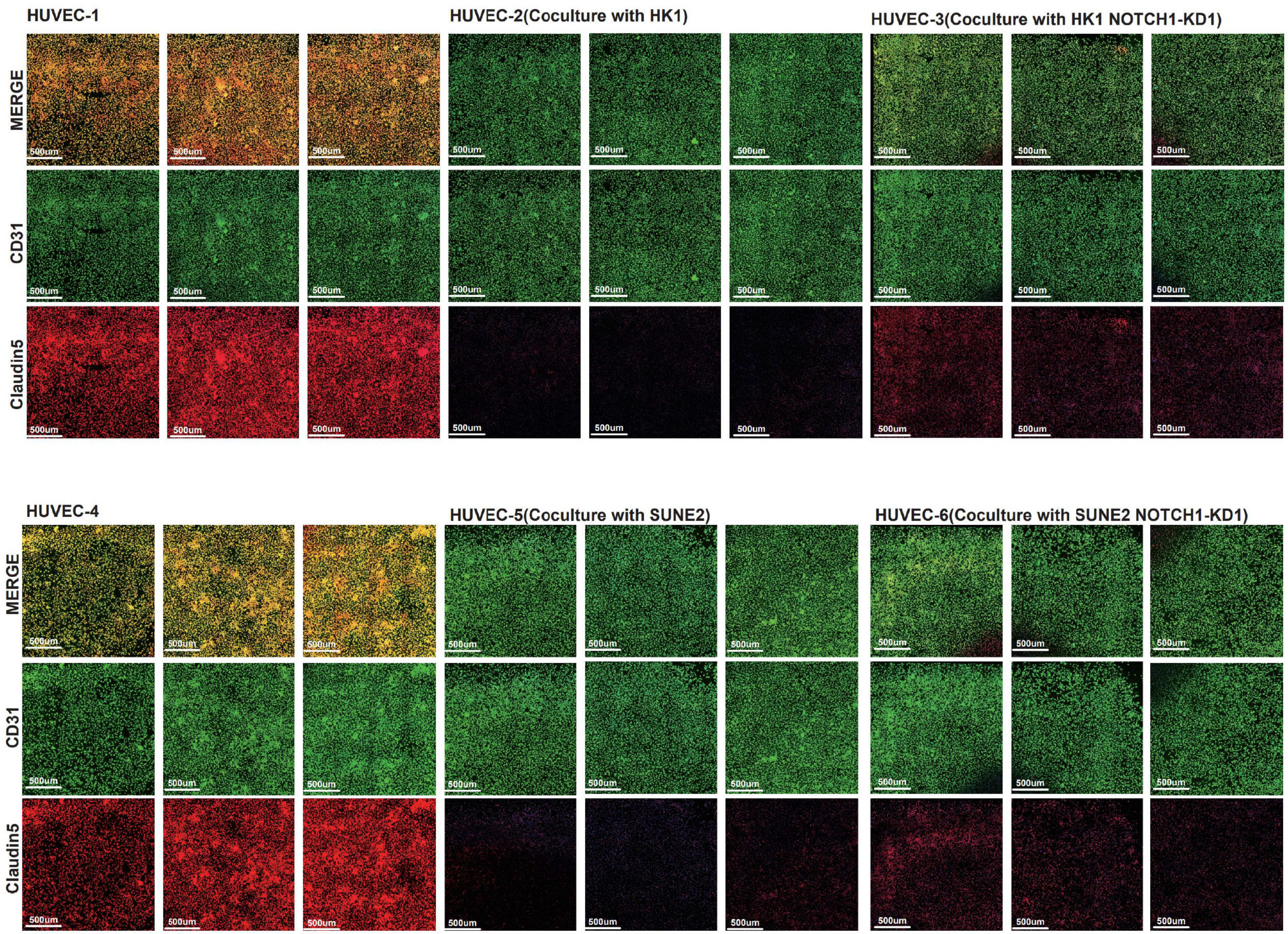
HUVEC-1
HUVEC-2(Coculture with HK1)
HUVEC-3(Coculture with HK1 NOTCH1-KD1)
MERGE
CD31
Claudin5
500um
HUVEC-4
HUVEC-5(Coculture with SUNE2)
HUVEC-6(Coculture with SUNE2 NOTCH1-KD1)

FigureS8 G

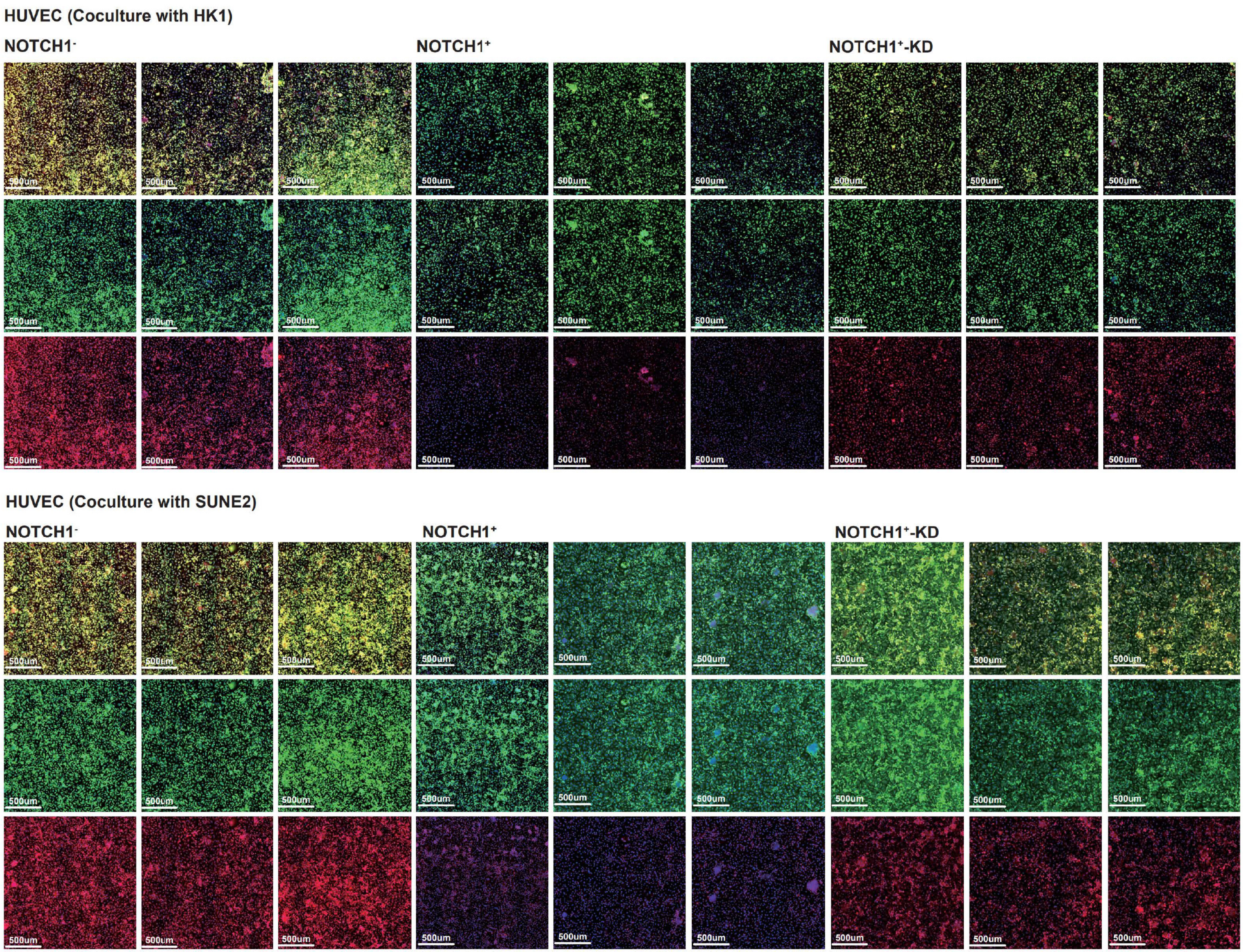
HUVEC (Coculture with HK1)
NOTCH1⁻
NOTCH1⁺
NOTCH1⁺-KD
500um
HUVEC (Coculture with SUNE2)
NOTCH1⁻
NOTCH1⁺
NOTCH1⁺-KD

FigureS12 B

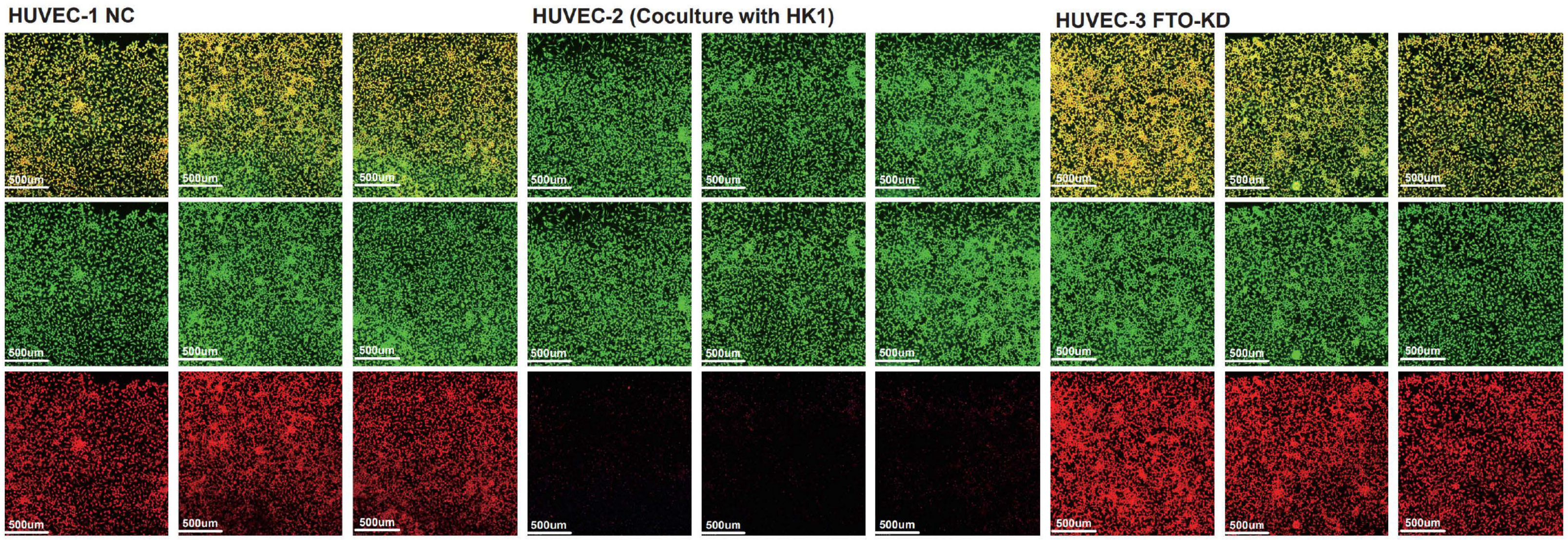
HUVEC-1 NC
HUVEC-2 (Coculture with HK1)
HUVEC-3 FTO-KD
500um

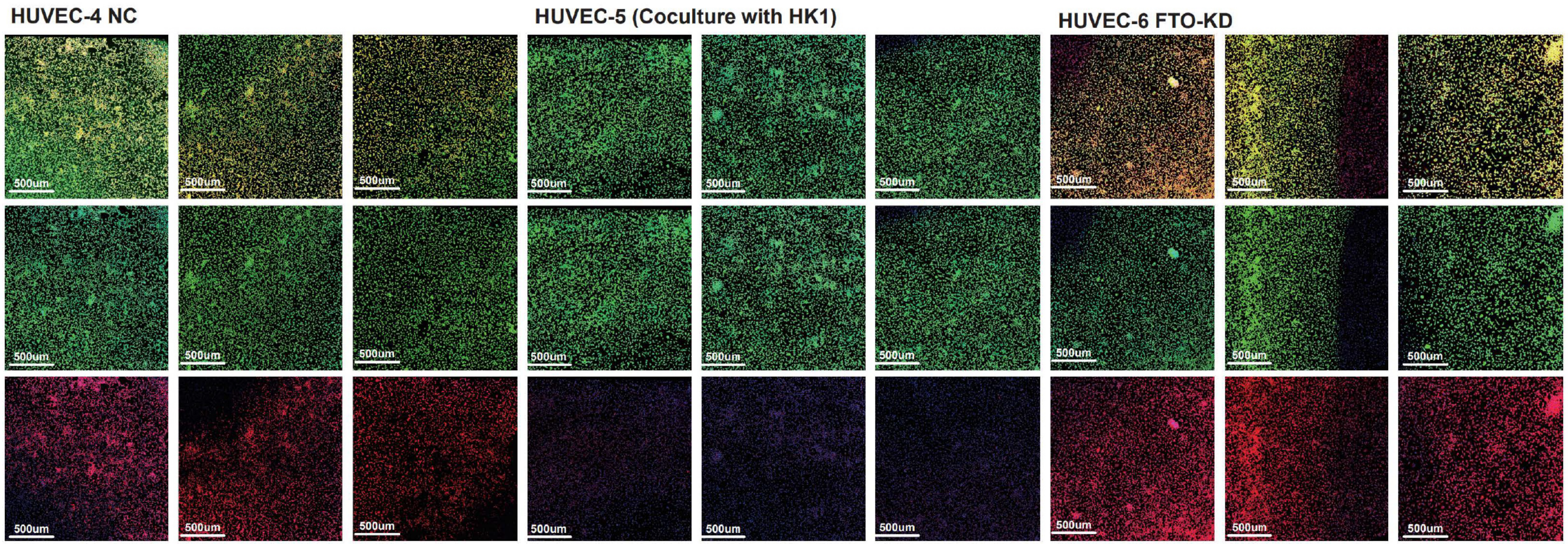
HUVEC-4 NC
HUVEC-5 (Coculture with HK1)
HUVEC-6 FTO-KD
500um

FigureS14 D

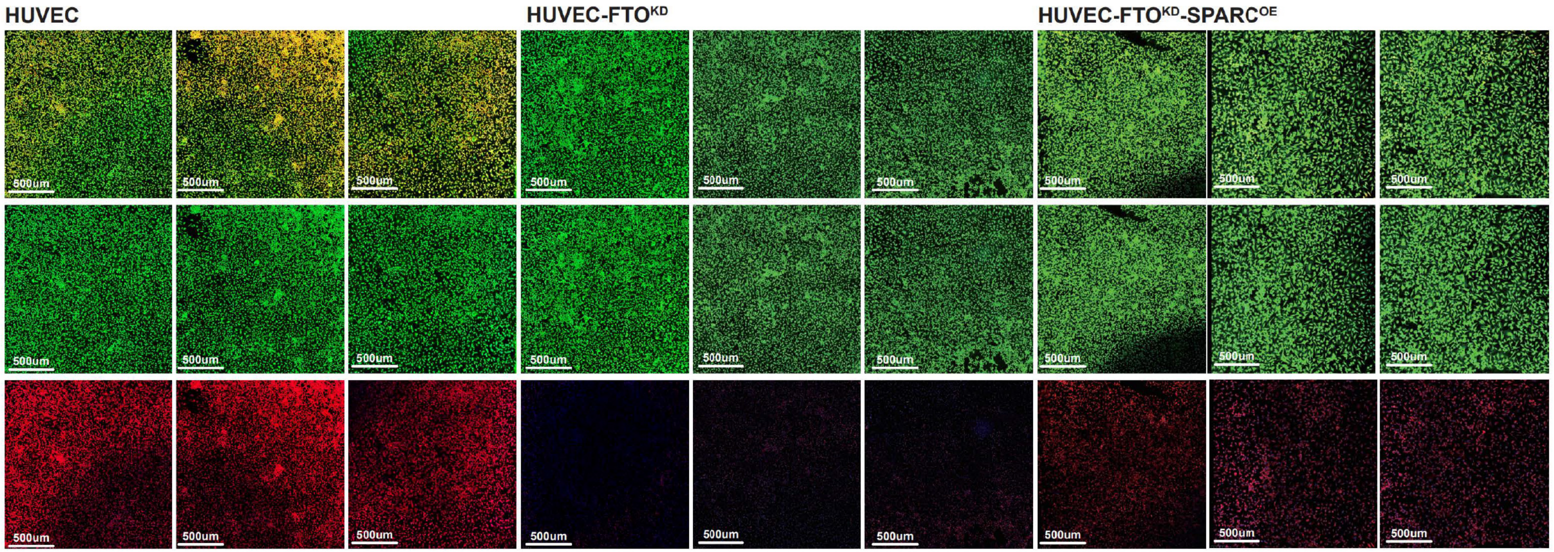

FigureS15 E

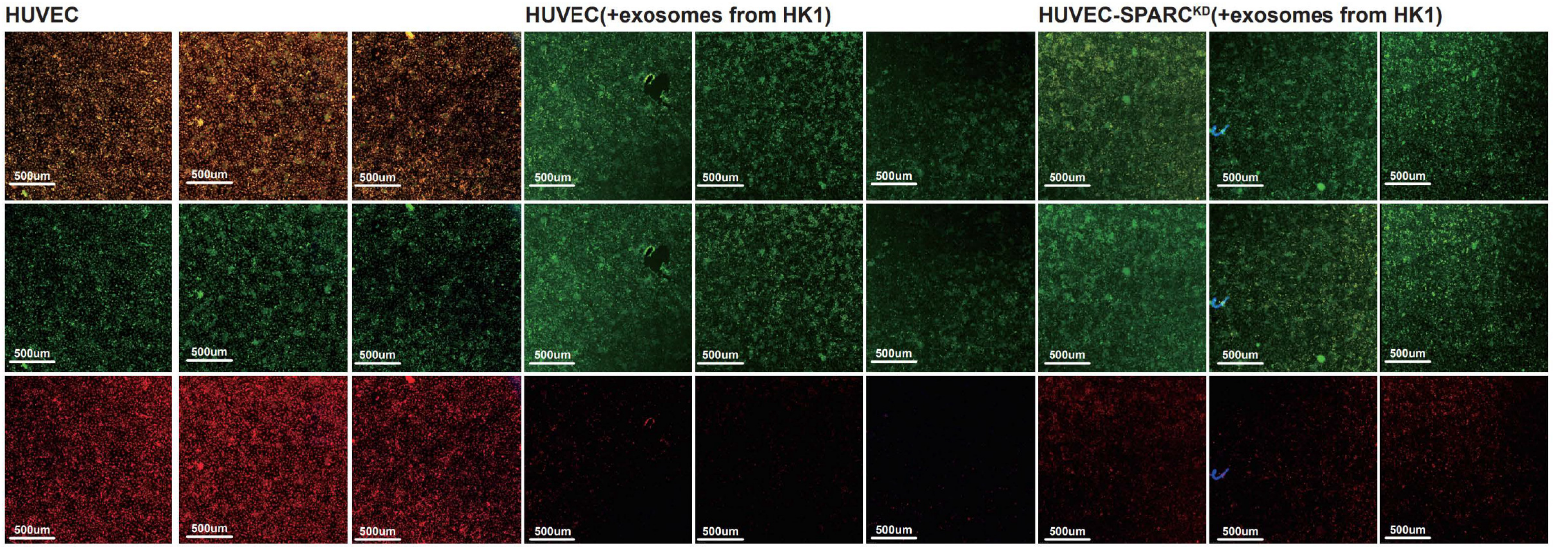
HUVEC
HUVEC(+exosomes from HK1)
HUVEC-SPARC^{KD}(+exosomes from HK1)
500um

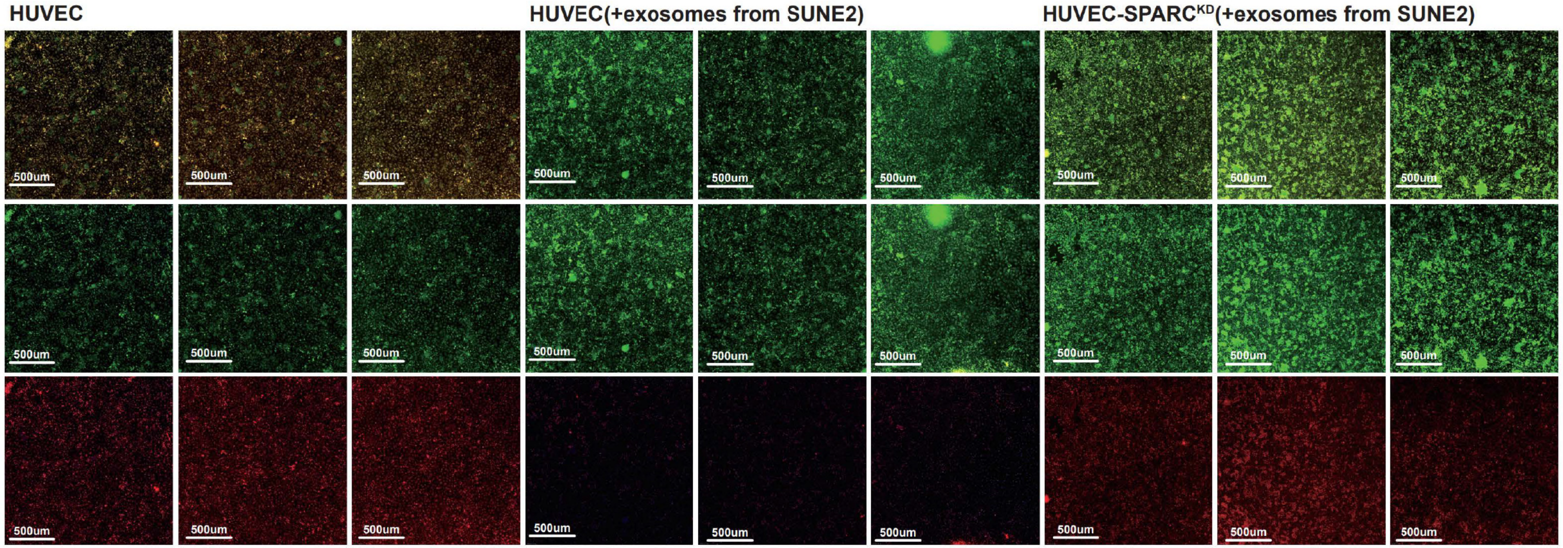
HUVEC
HUVEC(+exosomes from SUNE2)
HUVEC-SPARC^{KD}(+exosomes from SUNE2)
500um
