# Supplementary material for: The Tumor‐to‐Endothelial Transfer of FTO Promotes Vascular Remodeling and Metastasis in Nasopharyngeal Carcinoma
Source: Adv Sci (Weinh). 2025 Nov 28;13(8):e09524. doi: 10.1002/advs.202509524 (PMC12884774; doi:10.1002/advs.202509524)
Supplement: Supplementary file 4 — Supporting Information [file ADVS-13-e09524-s003.pdf]

High-resolution H&E, IHC and mIF images for Figures

NOTCH1 - NC

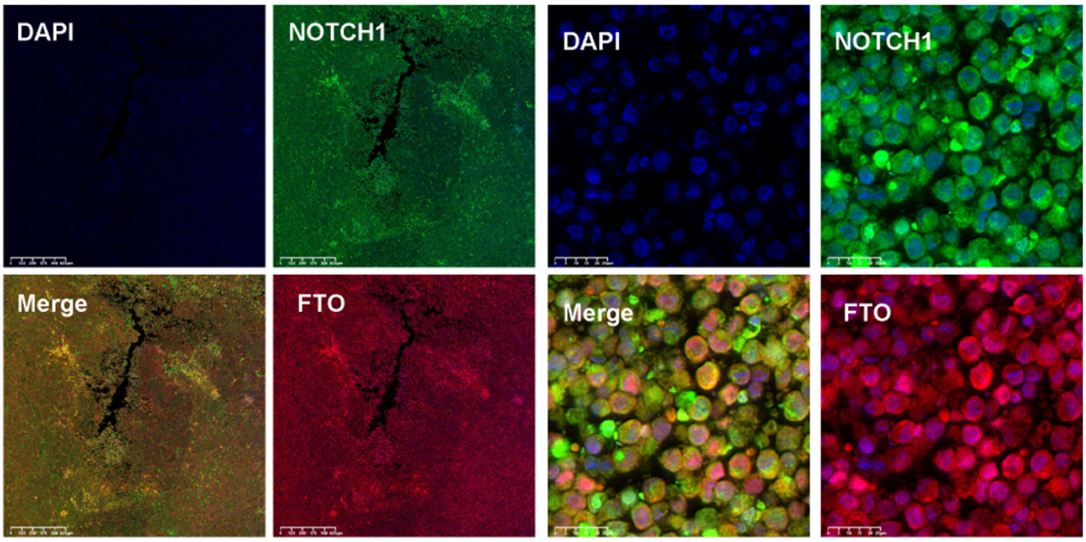

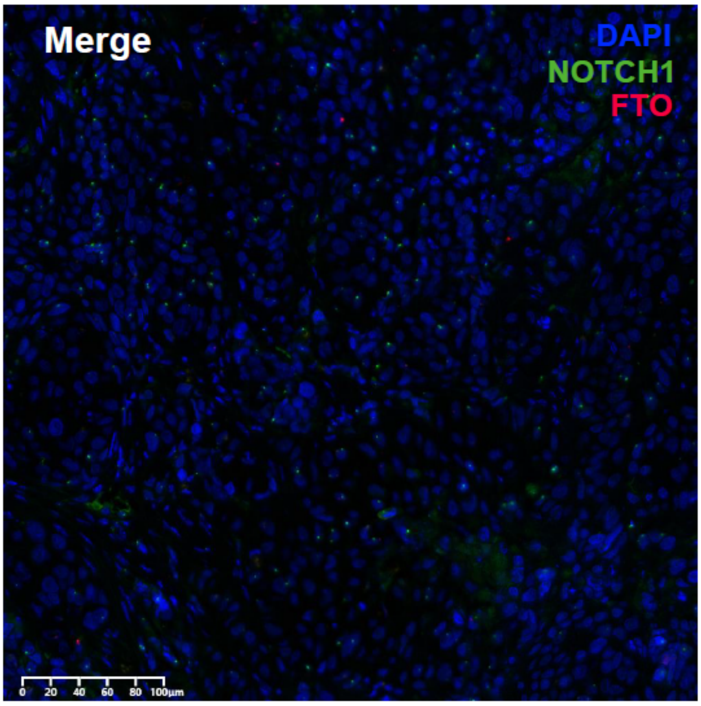

High-resolution image for Figure. 2K.

Nasopharyngeal carcinoma - 1

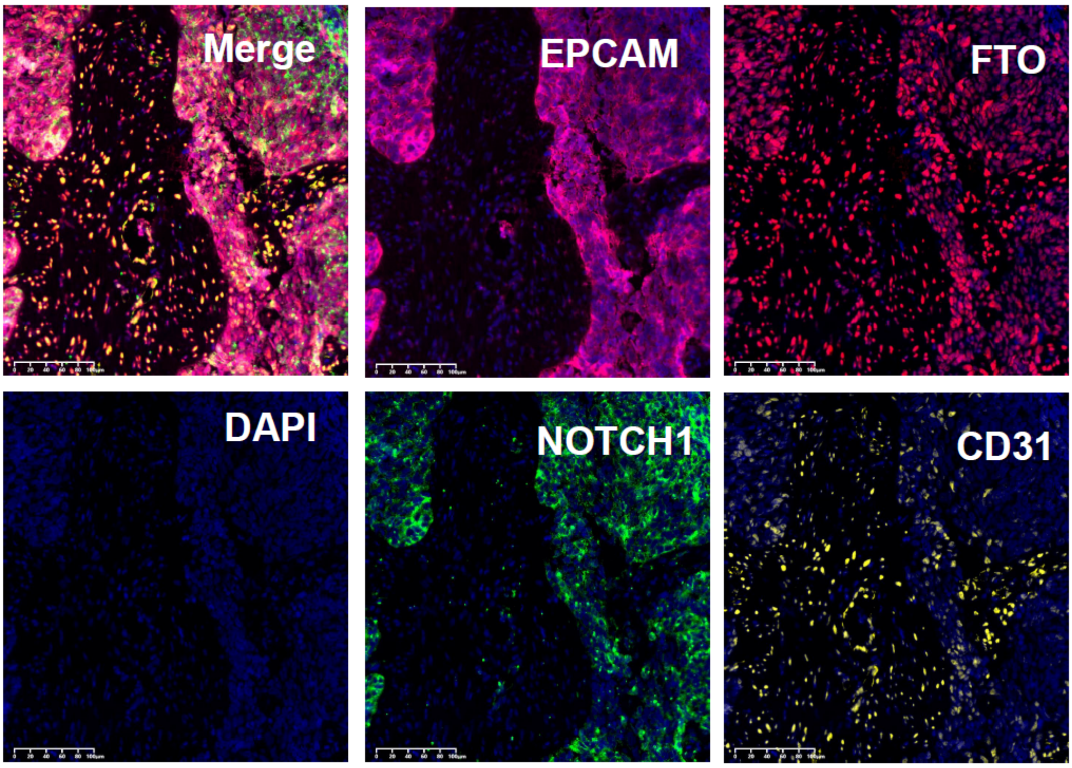

Nasopharyngeal carcinoma - 2

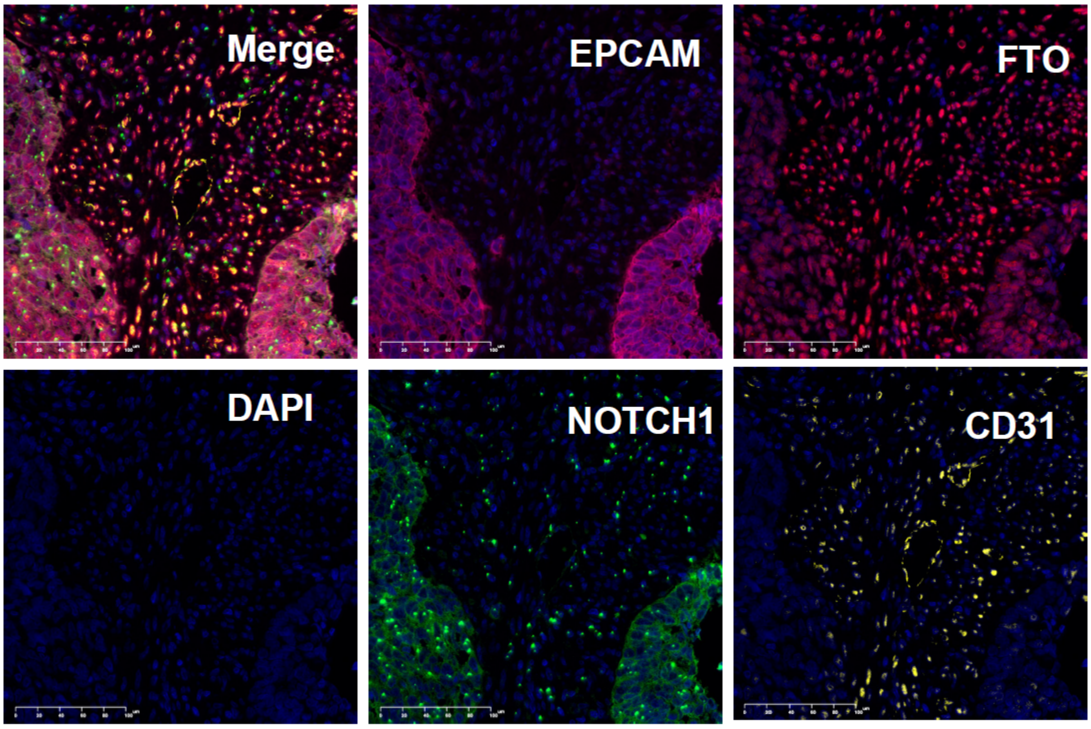

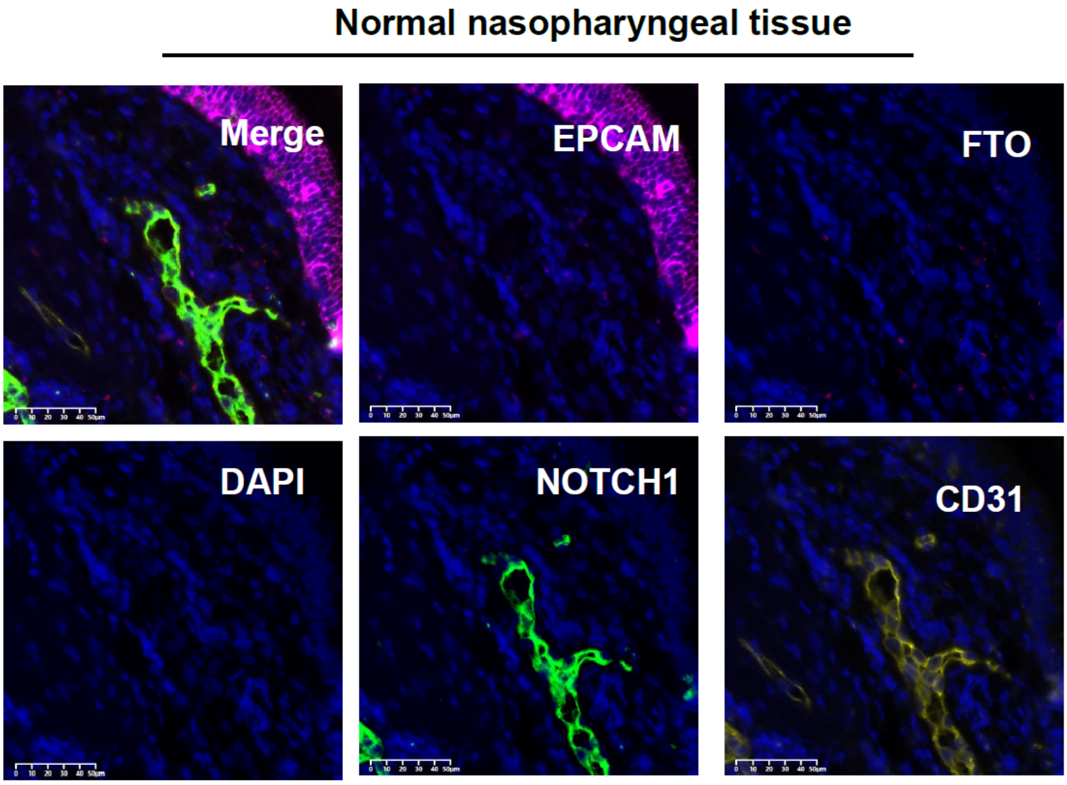

High resolution image for Figure. 2L.

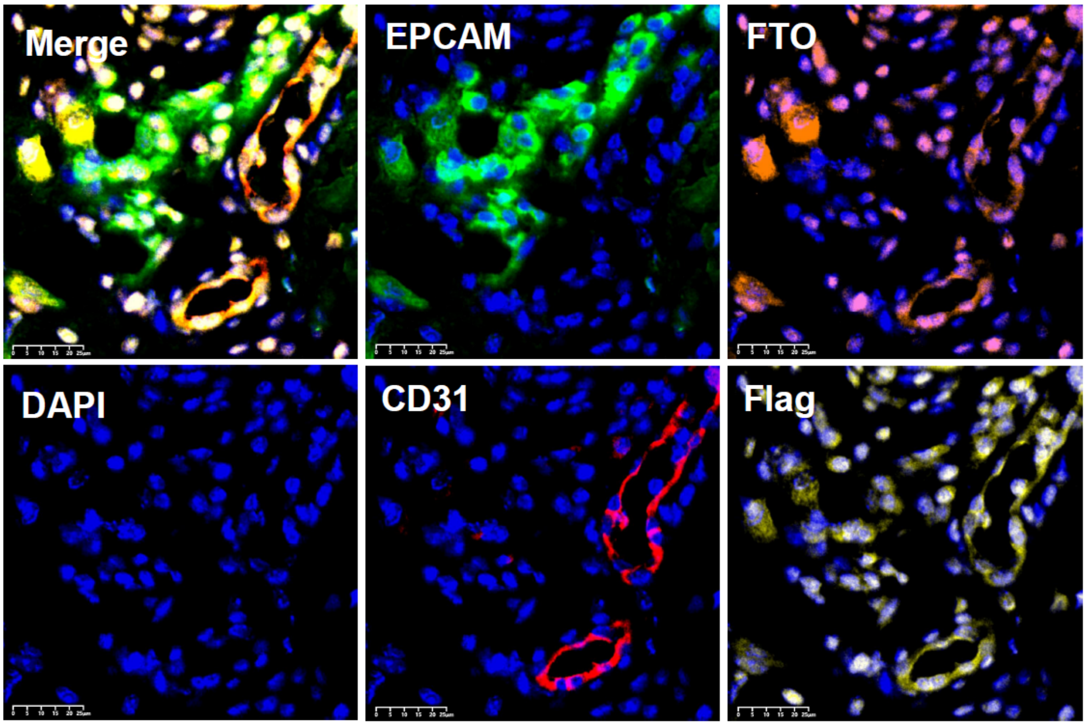

High-resolution images for Figure 3P.

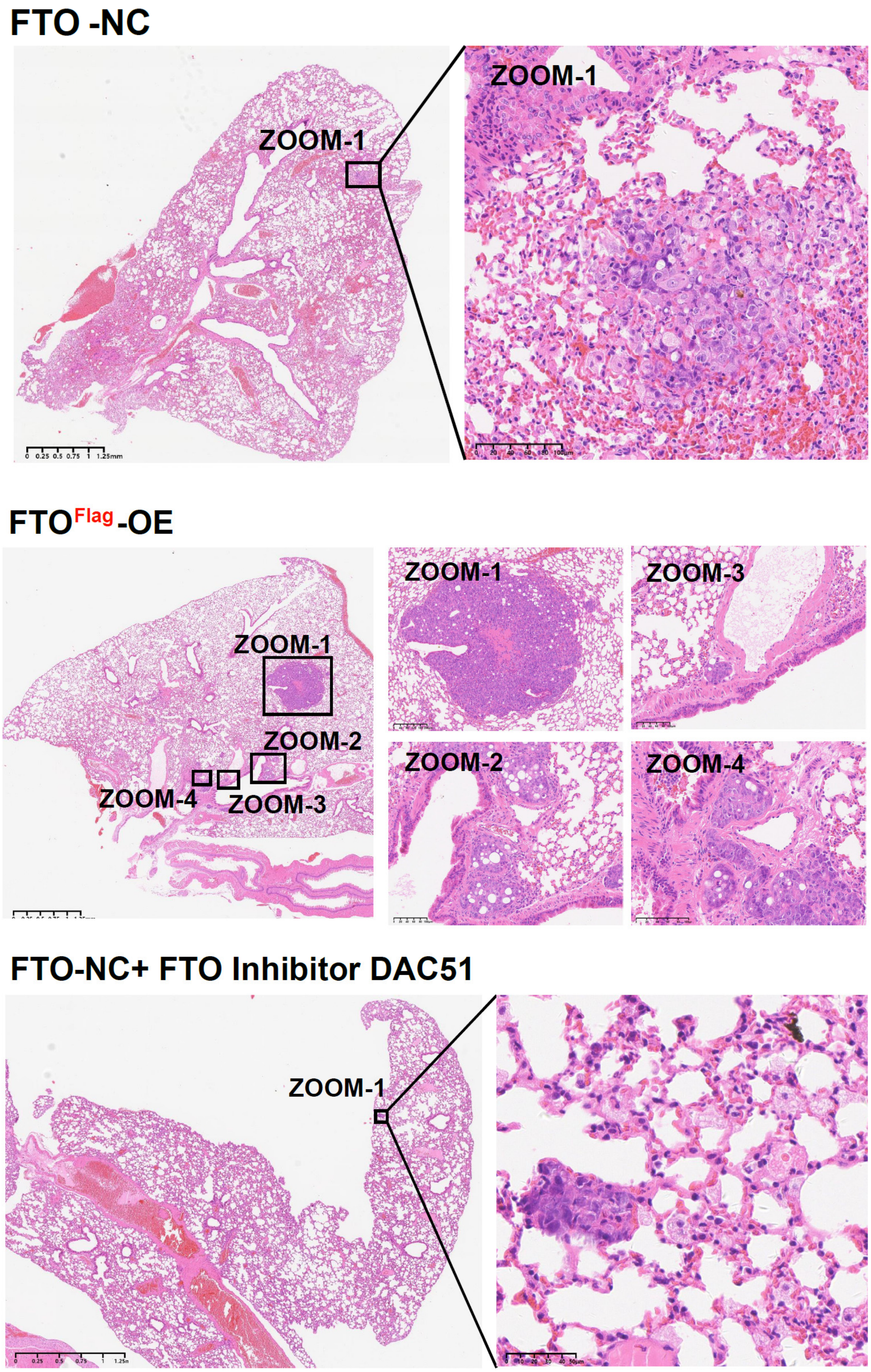

High-resolution image for Figure. 4H.

FTO^{Flag}-OE -ZOOM-2

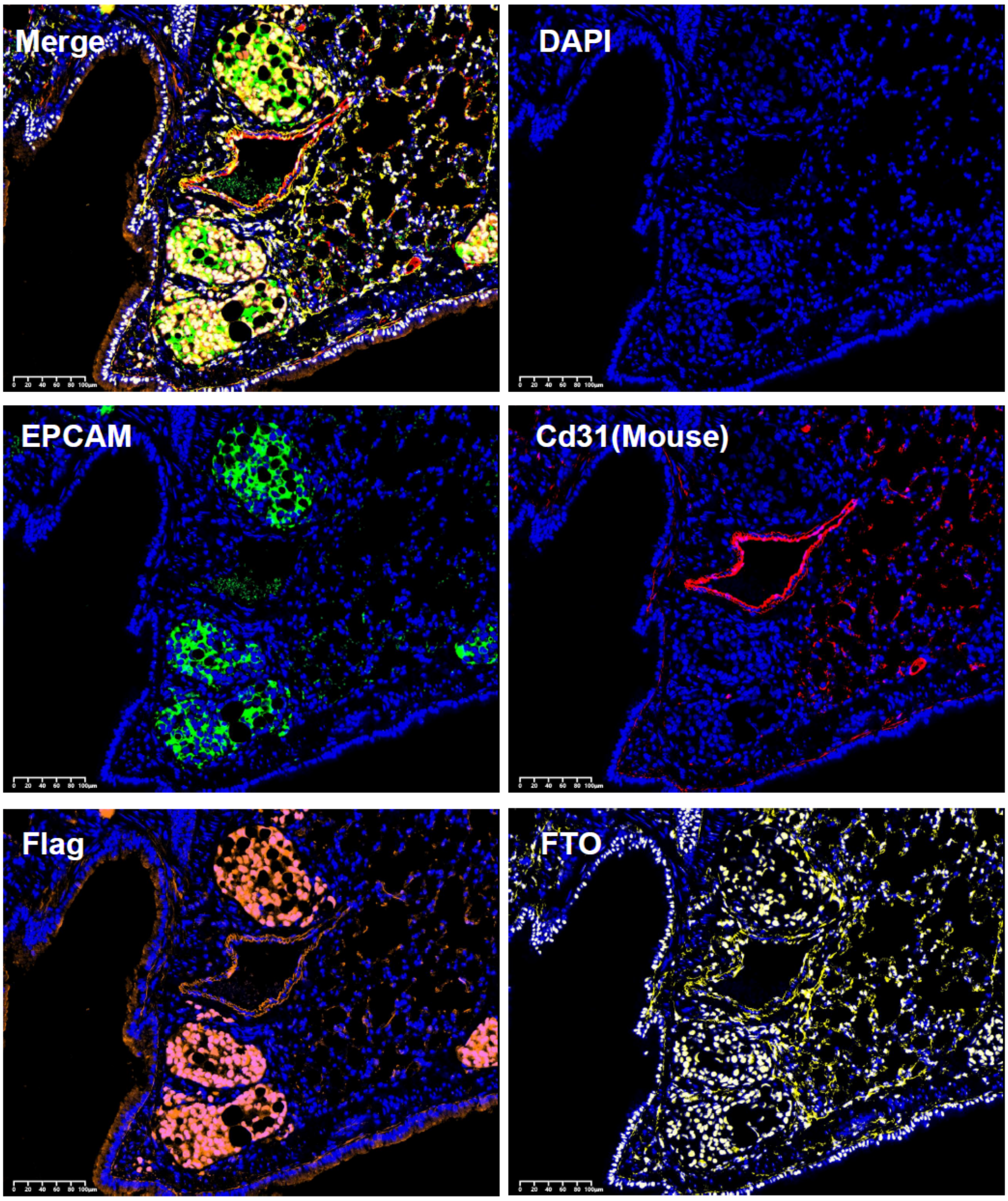

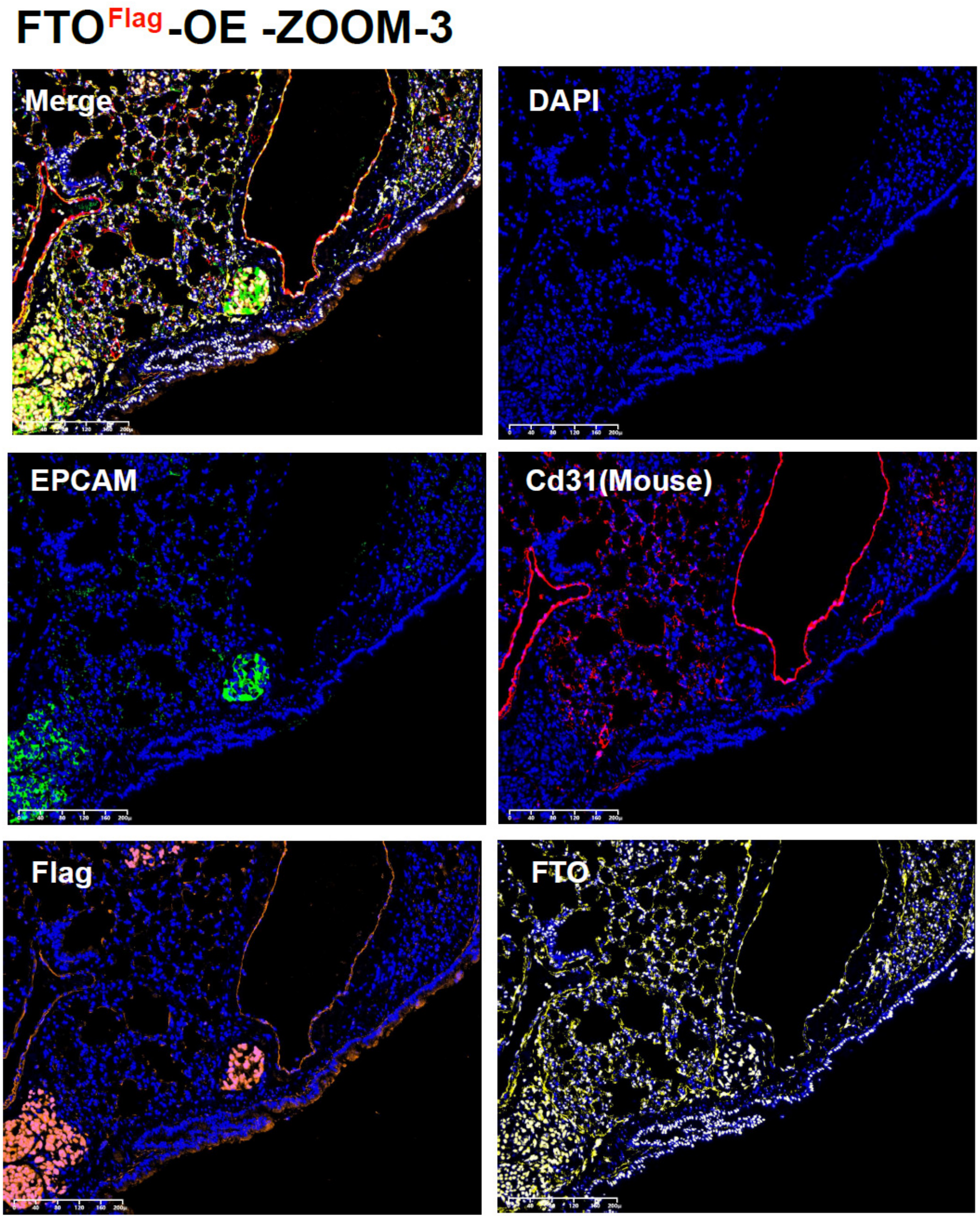

High-resolution image for Figure. 4I.

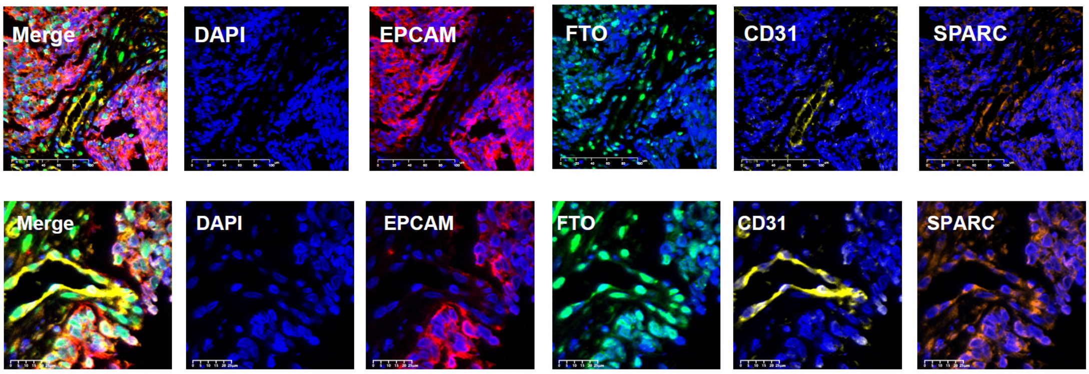

High-resolution image for Figure. 6E

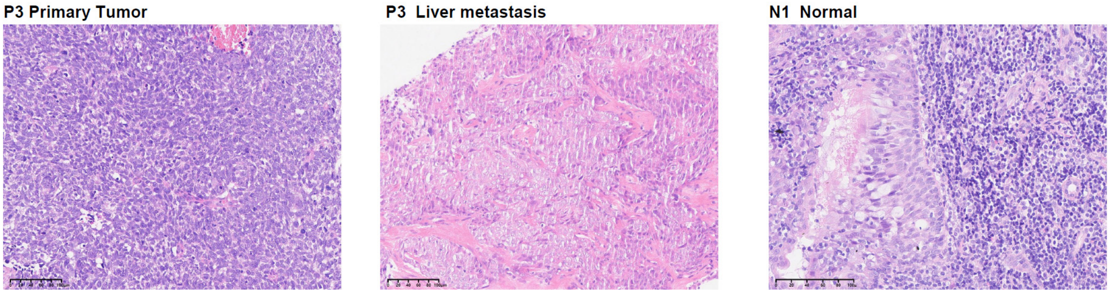

High-resolution image for Figure. S1A.

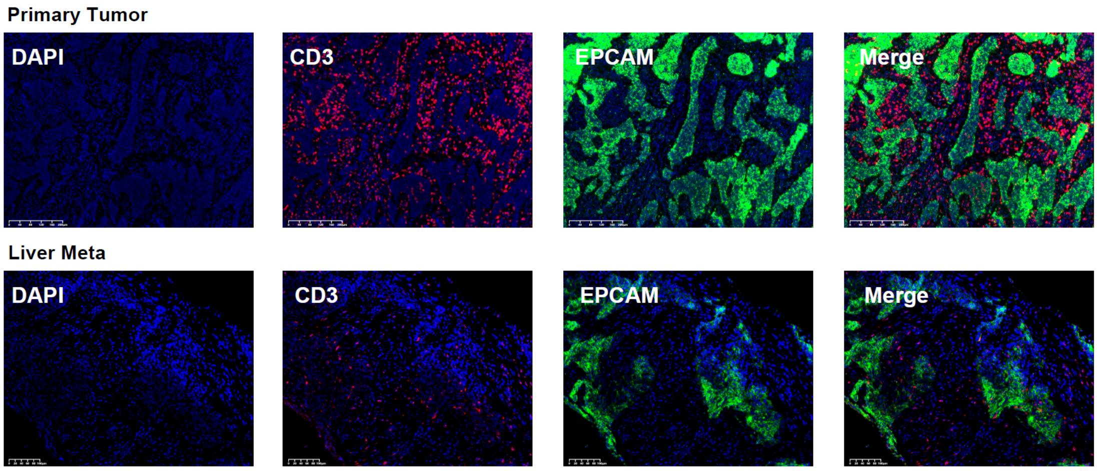

High-resolution image for Figure. S1E.

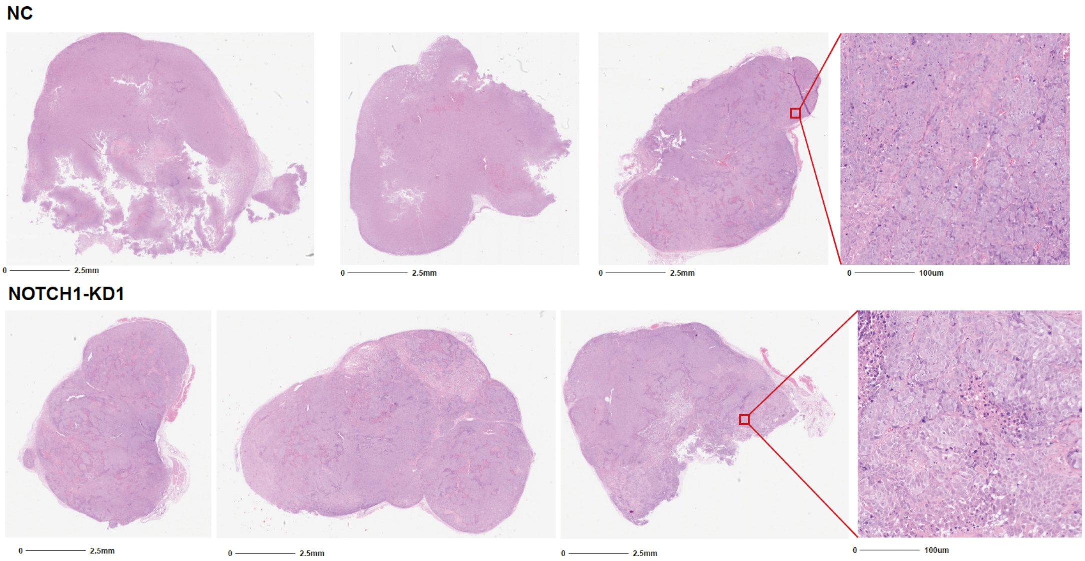

High-resolution image for Figure. S4B.

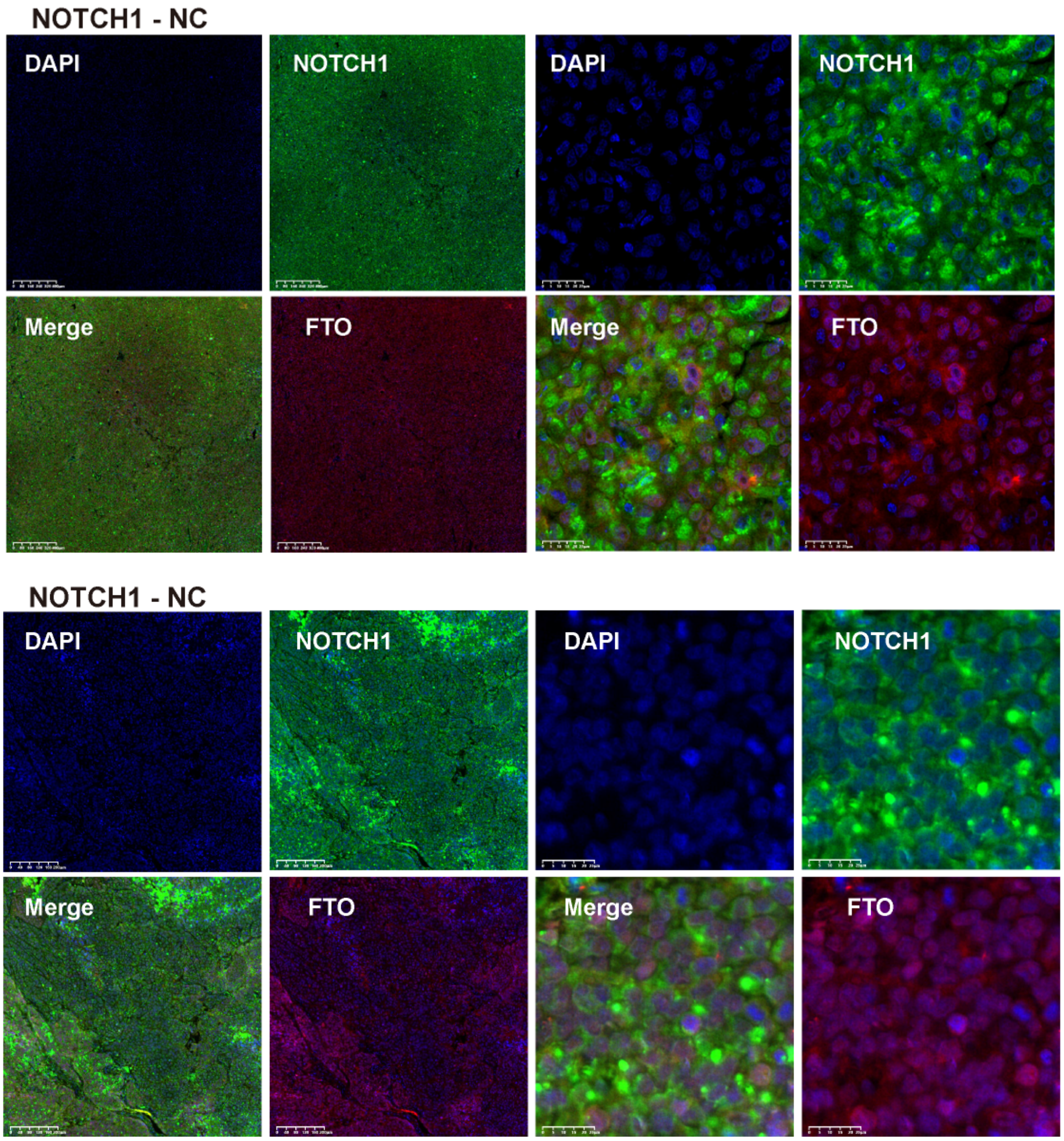

High-resolution image for Figure. S4C.

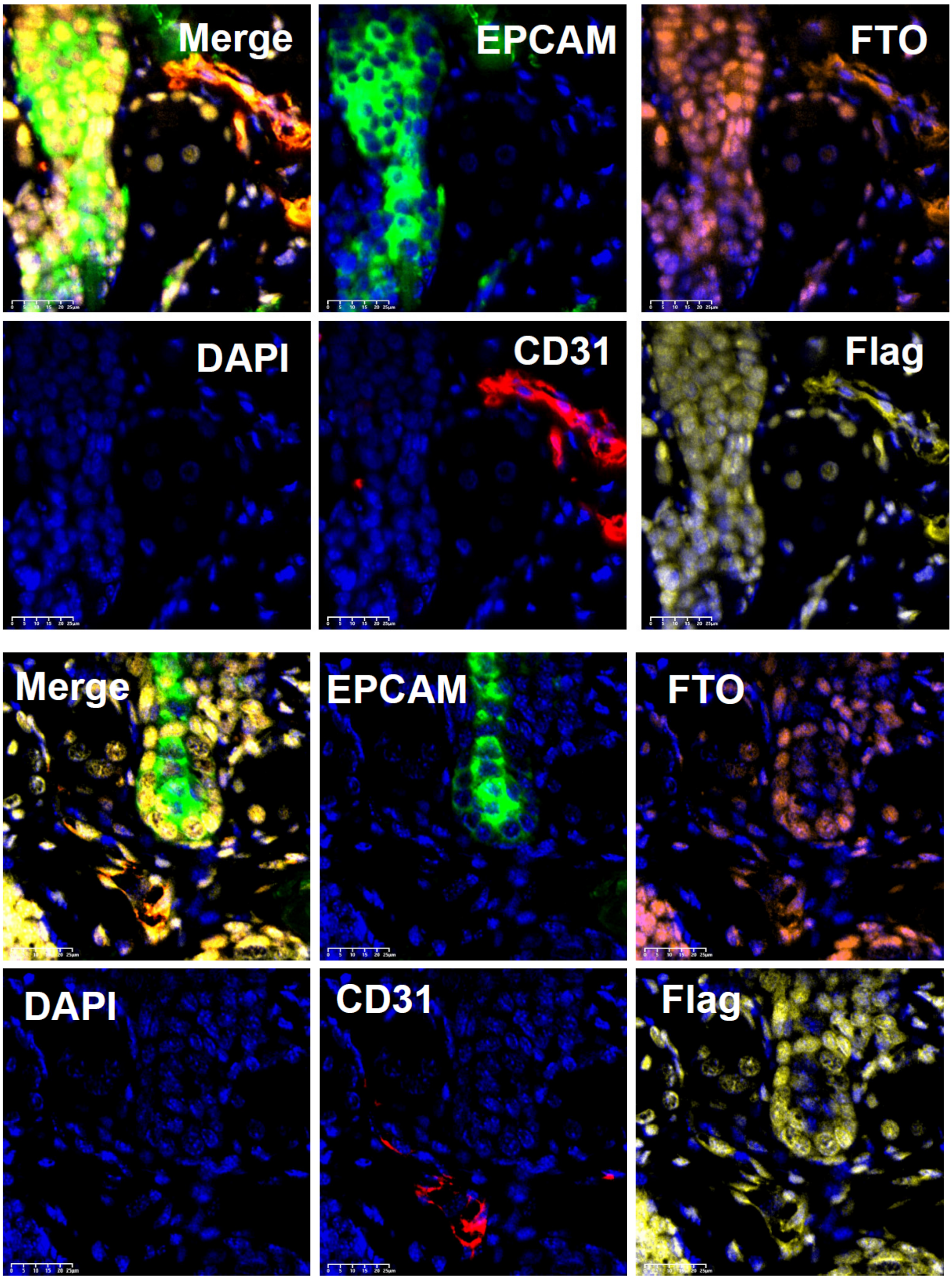

High-resolution image for Figure. S9A.

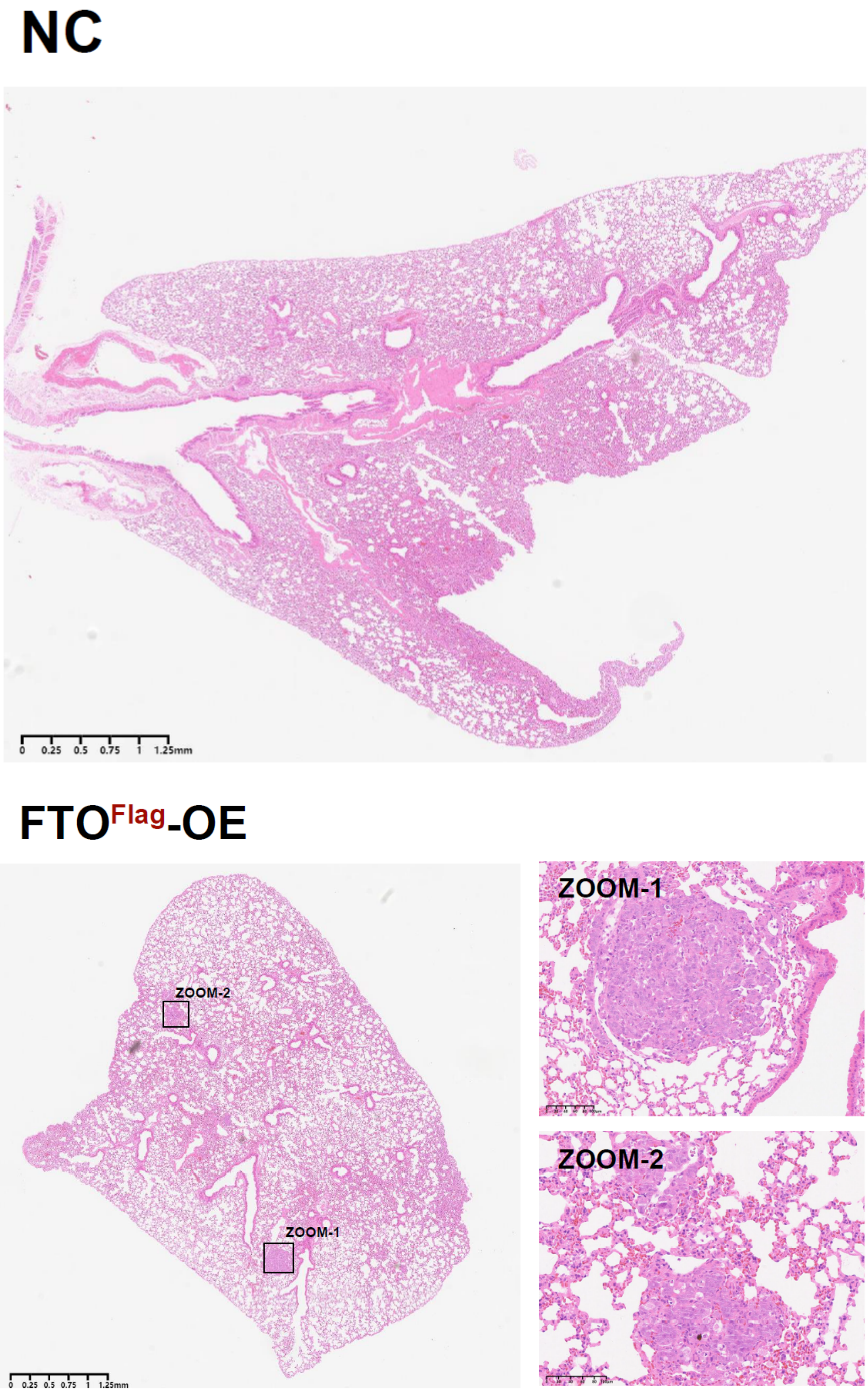

High-resolution image for Figure. S10B.

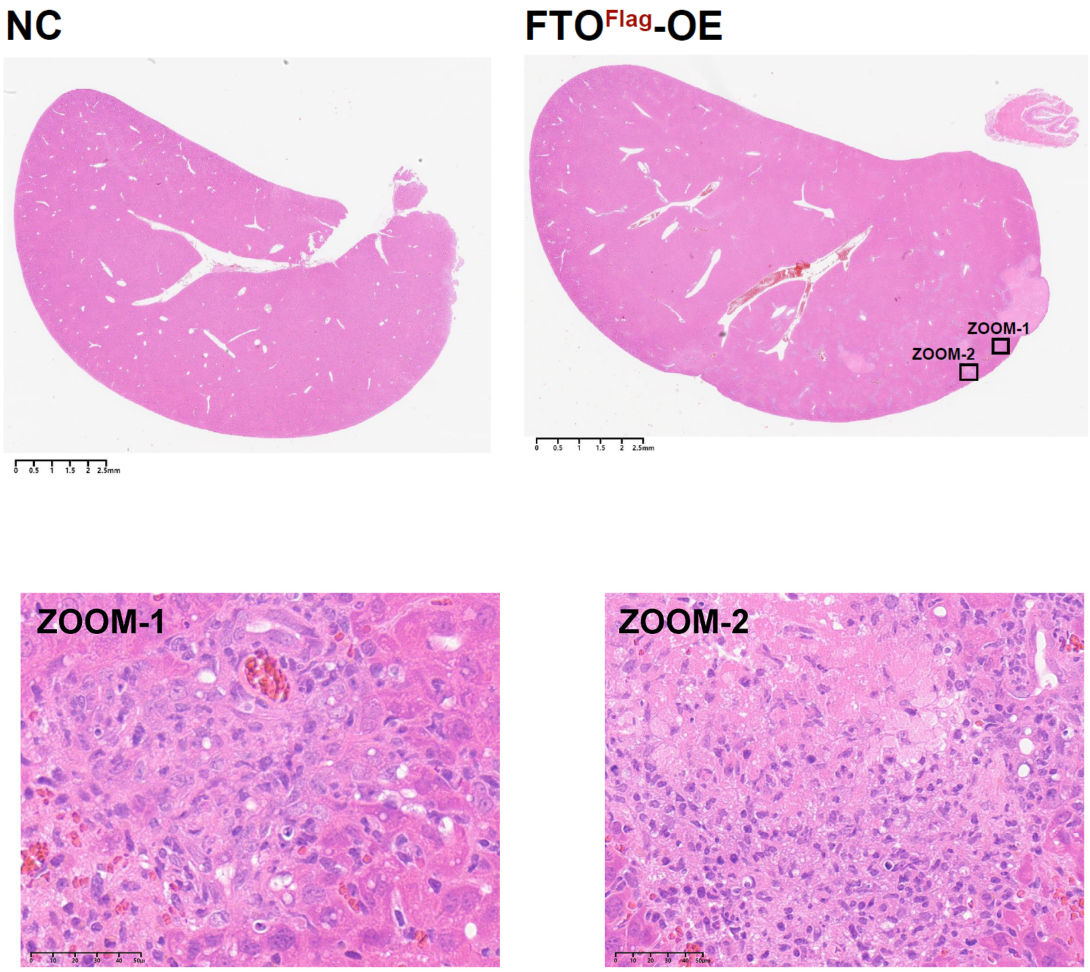

High-resolution image for Figure. S10C.

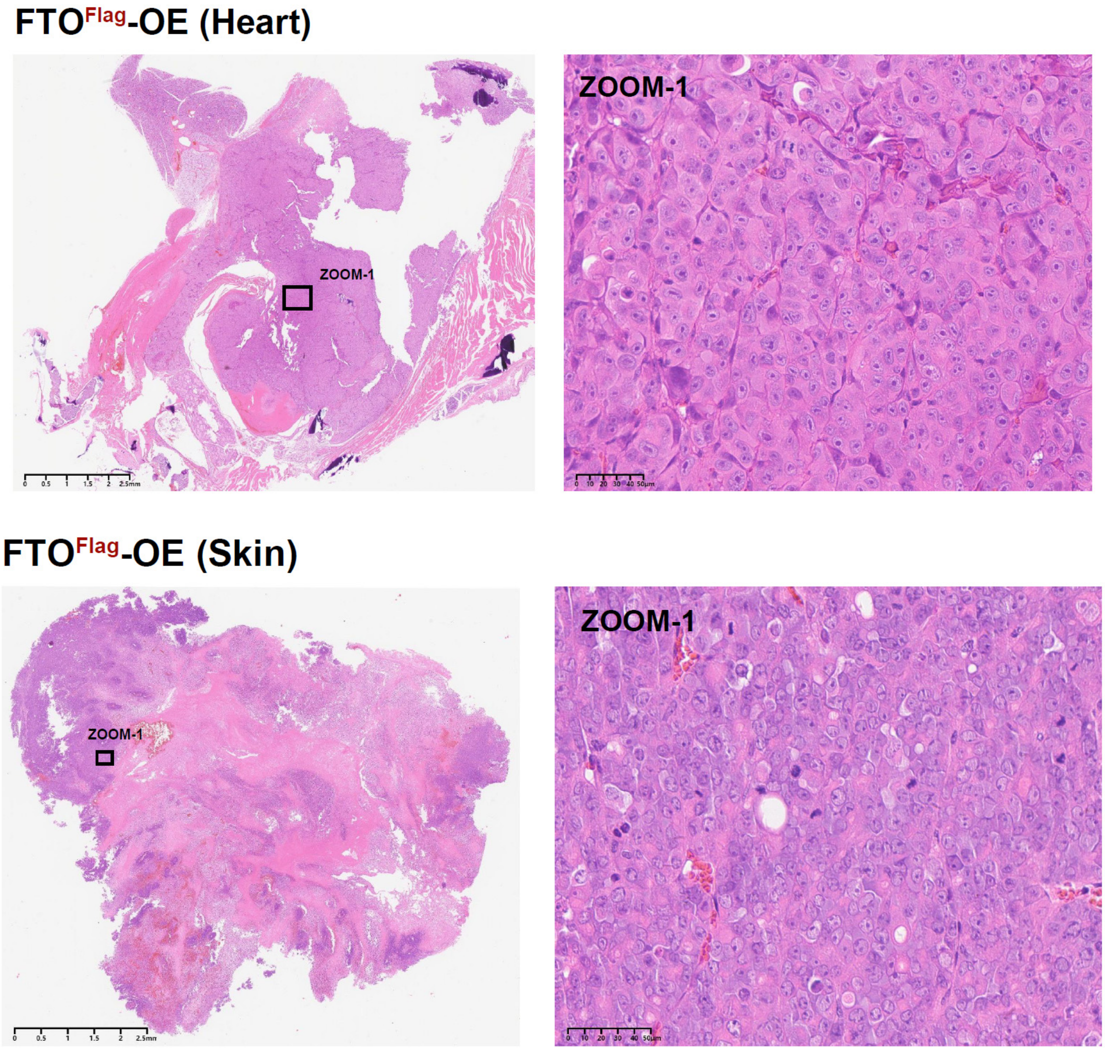

High-resolution image for Figure. S10D.

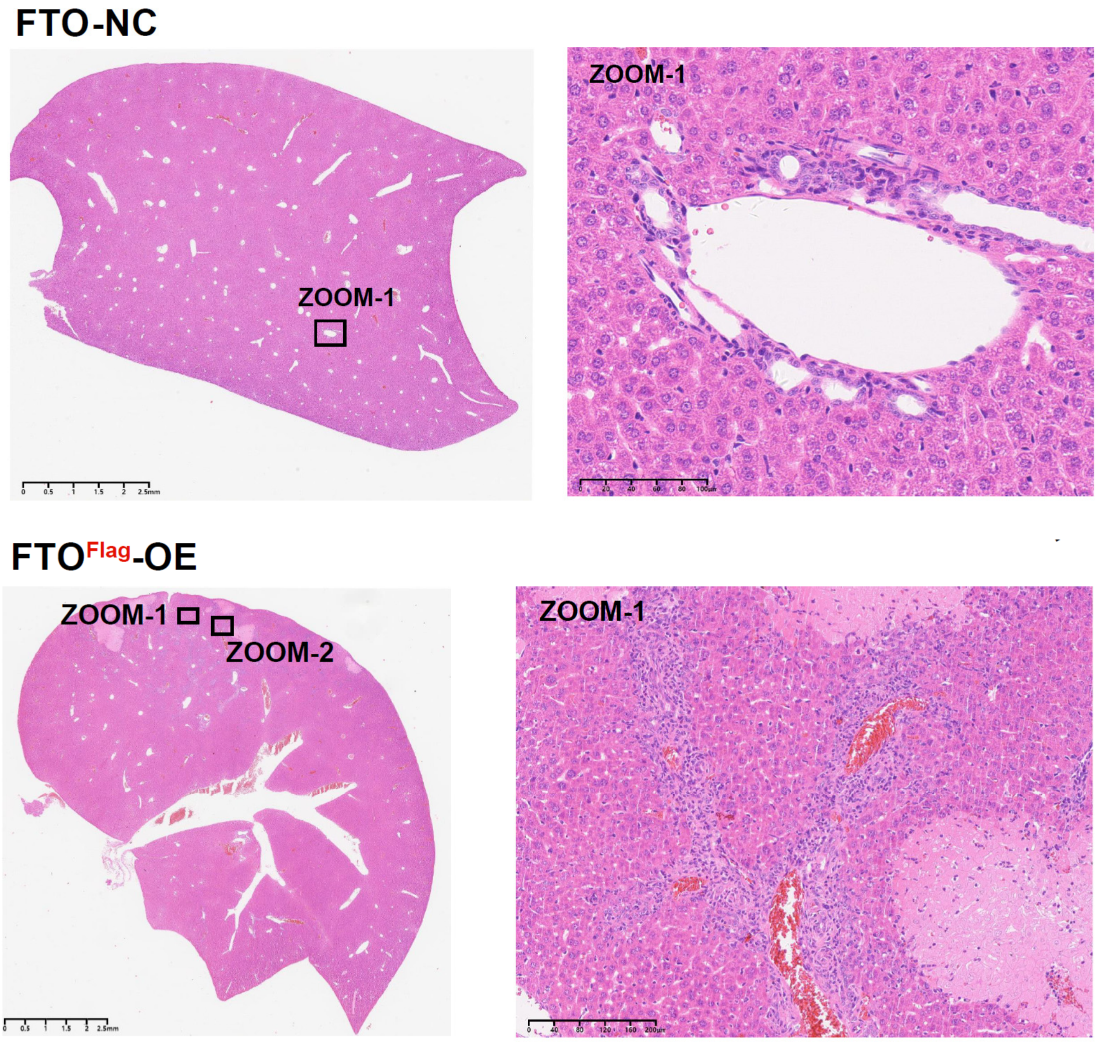
FTO-NC
ZOOM-1
ZOOM-1
FTOFlag-OE
ZOOM-1
ZOOM-2
ZOOM-1

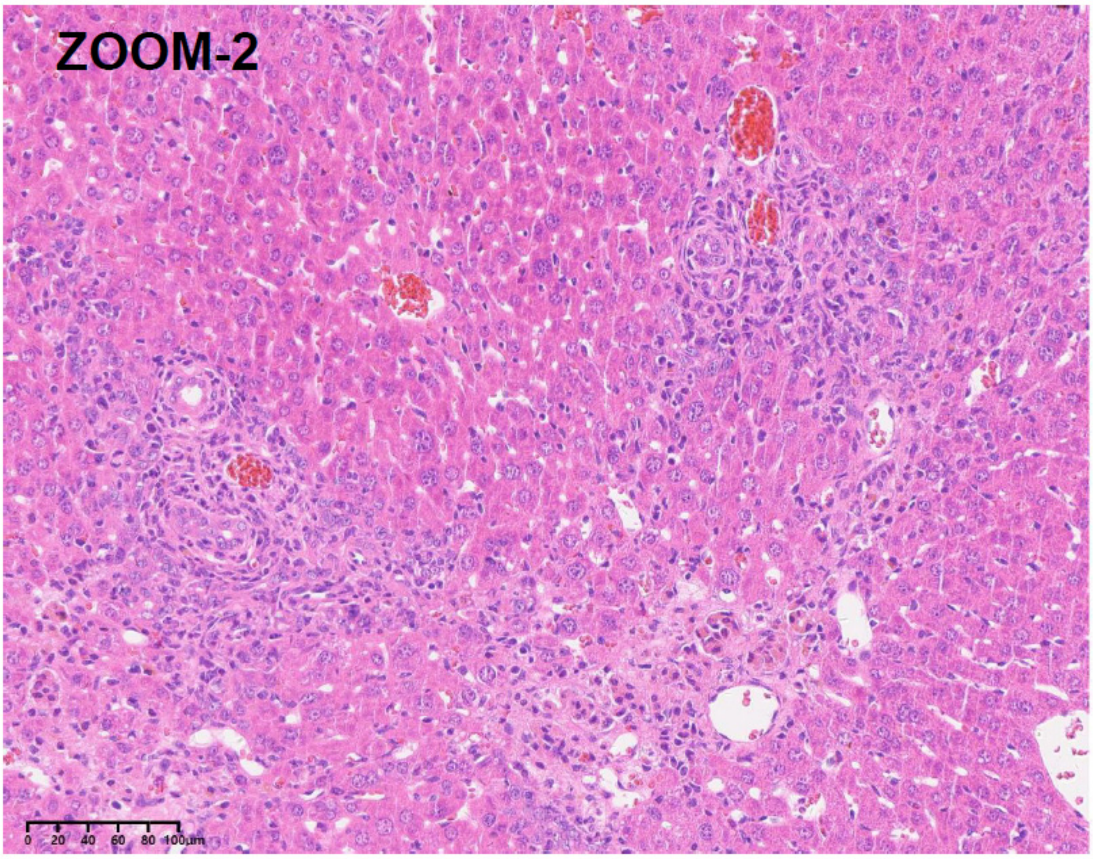

FTO-NC+ FTO Inhibitor DAC51

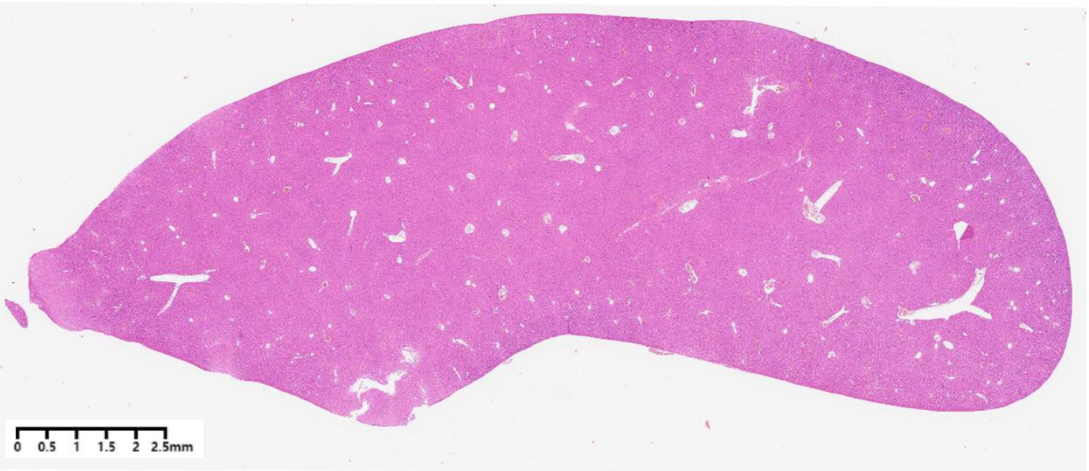

High-resolution image for Figure. S10G.

FTO^{Flag}-OE -ZOOM-1

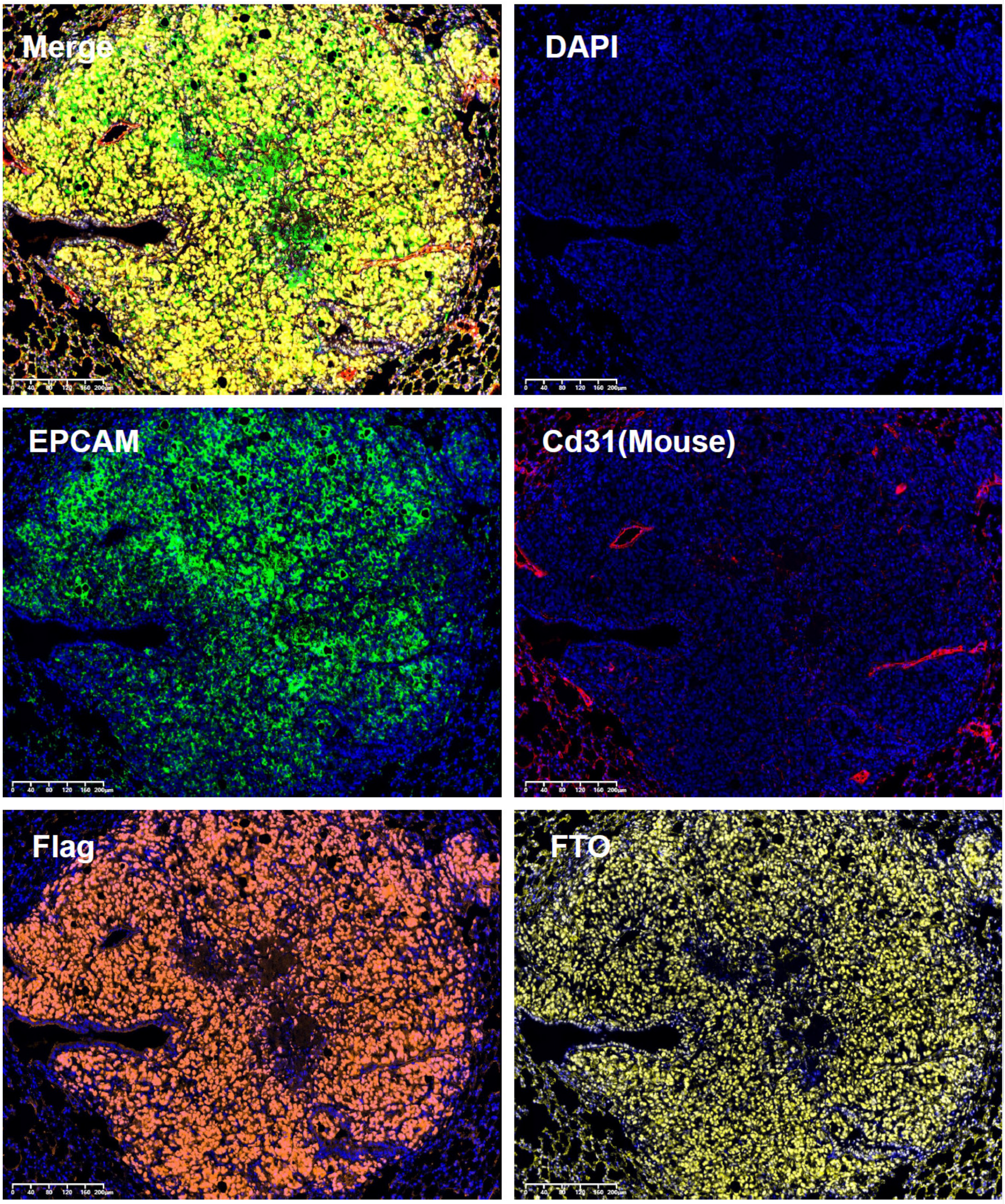

FTO^{Flag}-OE -ZOOM-4

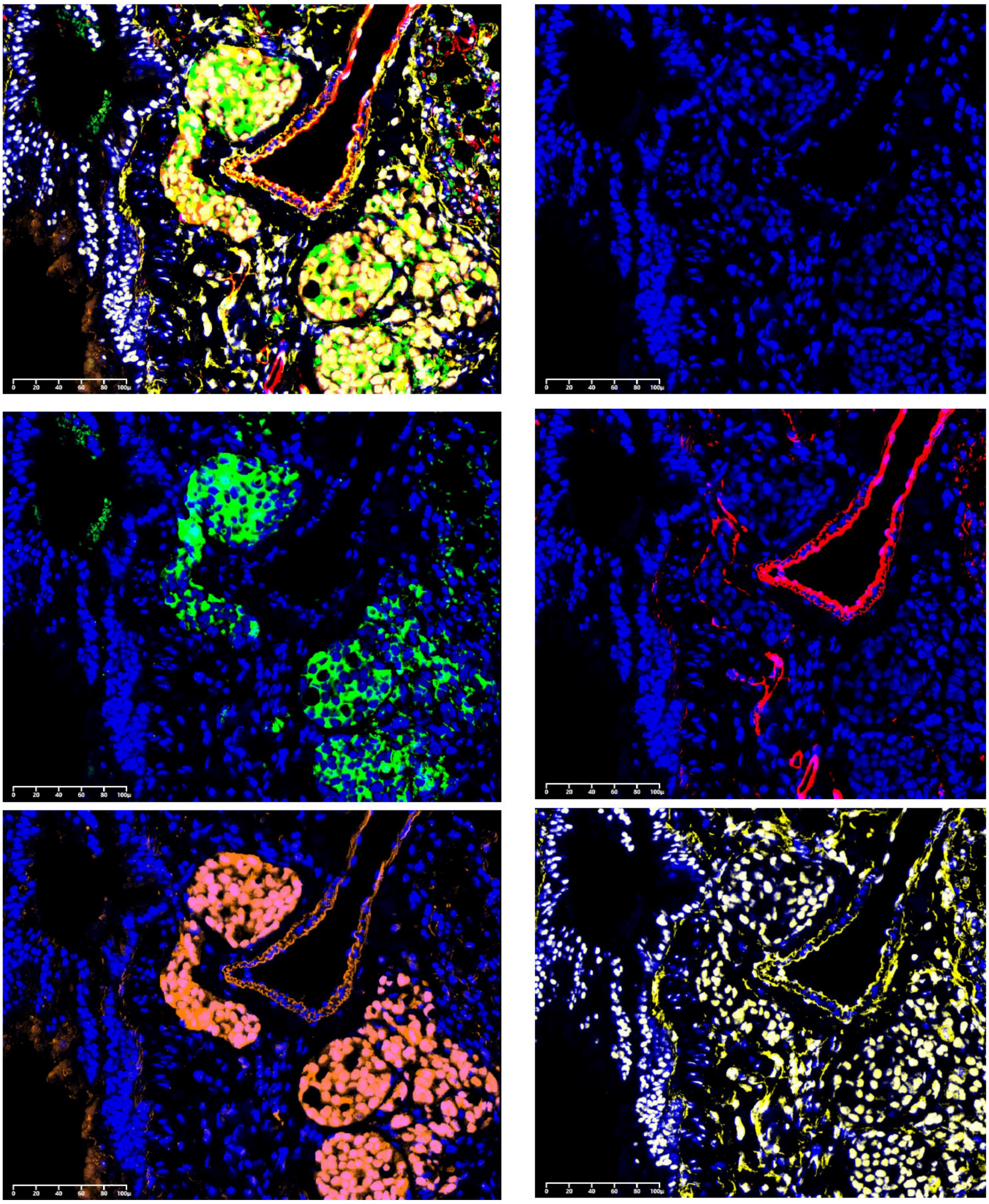

High-resolution image for Figure. S10H.

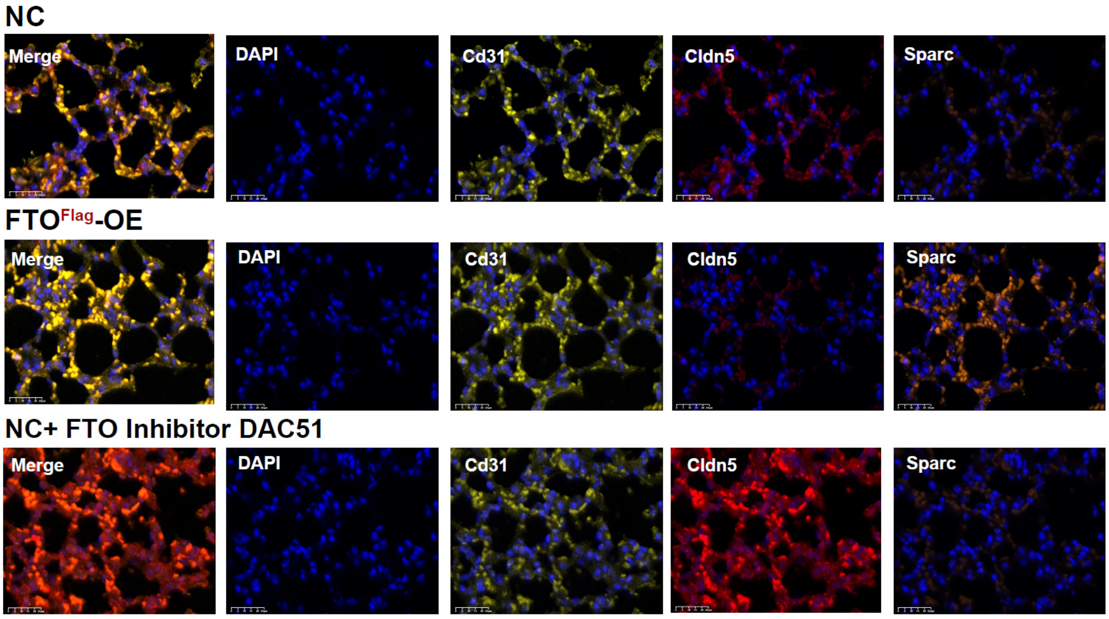

High-resolution image for Figure. S14I.

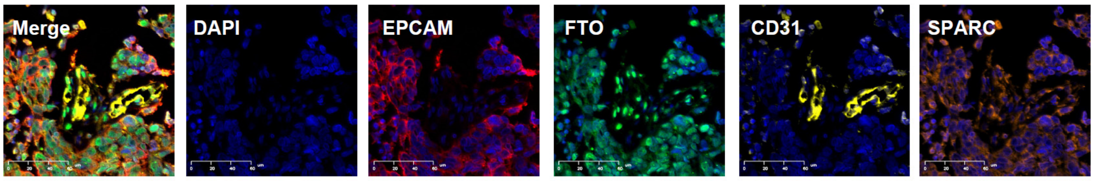

High-resolution image for Figure. S14E.
